# Socioeconomic inequalities in functional ageing trajectories in England and Canada: A comparative longitudinal cohort study

**DOI:** 10.1371/journal.pmed.1004833

**Published:** 2026-08-13

**Authors:** Stephanie Schrempft, Shelby Vereecke, Mayssam Nehme, Kim L. Schmidt, Idris Guessous, Michael S. Kobor, Paola Zaninotto, Silvia Stringhini

**Affiliations:** 1 Division of Primary Care Medicine, Geneva University Hospitals, Geneva, Switzerland; 2 School of Population and Public Health, Faculty of Medicine, University of British Columbia, Vancouver, Canada; 3 Faculty of Medicine, University of Geneva, Geneva, Switzerland; 4 Edwin S.H. Leong Centre for Healthy Aging, Faculty of Medicine, University of British Columbia, Vancouver, Canada; 5 Department of Epidemiology and Public Health, University College London, London, United Kingdom; University of Bern Faculty of Natural Sciences: Universitat Bern Philosophisch-naturwissenschaftliche Fakultat, SWITZERLAND

## Abstract

**Background:**

Cross-country comparisons can provide valuable insights into the societal and policy contexts that shape socioeconomic inequalities in ageing. However, longitudinal comparative research in this area is scarce. We therefore examined associations between socioeconomic conditions and functional ageing trajectories in a longitudinal comparison between England and Canada.

**Methods and findings:**

In this longitudinal cohort study, data were drawn from the English Longitudinal Study of Ageing (ELSA, 2012–2023, *N* = 8511, 55% women, aged 50–85 years at baseline) and the Canadian Longitudinal Study on Aging (CLSA, 2012–2021, *N* = 22,605, 49% women, aged 50–85 years at baseline). Associations between total household wealth and physical functioning (gait speed, grip strength, chair stands, lung function, self-rated hearing, self-rated eyesight, mobility difficulties, and difficulties performing activities of daily living) were examined within each cohort using mixed-effects models, with additional adjustment for chronic conditions, weight status, health behaviours, and social connections. In each cohort, less wealth was associated with a poorer level of functioning across outcomes, and a greater decline in mobility and the ability to perform activities of daily living with age. In ELSA, wealthier individuals experienced a steeper decline in gait speed compared to their less wealthy counterparts, although their level remained higher throughout the follow-up period. In CLSA, wealthier individuals had a steeper decline in chair rise performance and self-rated eyesight compared to less wealthy individuals. Wealth inequalities in the level of functioning were generally greater in ELSA than in CLSA, especially for gait speed (years of functioning lost at age 60 = 15, 95% CI [12.2, 17.8] versus 9, 95% CI [8.6, 9.4]) and difficulties with basic activities of daily living (20, 95% CI [19.5, 20.4] versus 9, 95% CI [8.6, 9.4]). There were some significant wealth by sex interaction effects, such that women with the least wealth had more difficulties with mobility, activities of daily living, and hearing than men with the least wealth, but better lung function. Associations between wealth and functional outcomes were attenuated but persisted after adjustment for chronic conditions, weight status, health behaviours, and social connections. Limitations include the focus on socioeconomic conditions in adulthood and the inability to examine the contribution of other important factors to the observed associations, such as access to private healthcare.

**Conclusions:**

Socioeconomic inequalities in functional ageing are apparent in England and Canada, are larger in England than in Canada, and are not fully explained by common risk factors. These findings suggest that multilevel approaches, combining individual-level interventions with structural measures, are required to address socioeconomic inequalities and cross-country differences in functional ageing. Variation in wealth associations by sex suggests that approaches to reducing socioeconomic inequalities in functional health should account for potential sex differences.

## Author summary

**Why was this study done?:**

- Wealth gradients in functional health and disability are well-documented, but cross-country comparisons of functional ageing trajectories remain limited.
- Comparing countries can help identify the structural factors and processes contributing to social inequalities in functional health and ageing.
- An intersectional approach, which examines the influence of sex and race together with wealth, can provide additional insights into the processes contributing to social inequalities.

**What did the researchers do and find?:**

- Using data from the English Longitudinal Study of Ageing (ELSA, 2012–2023, *N* = 8511) and the Canadian Longitudinal Study on Aging (CLSA, 2012–2021, *N* = 22,605), we found that less wealth was associated with a poorer level of functioning across outcomes, and a greater decline in mobility and the ability to perform activities of daily living with age within each cohort.
- Wealth inequalities in the level of functioning were generally larger in ELSA than in CLSA, especially for gait speed and difficulties with basic activities of daily living.
- Associations between wealth and functional outcomes persisted after accounting for chronic conditions, weight status, health behaviours, and social connections; while women with the least wealth had more difficulties with mobility, activities of daily living, and hearing than men with the least wealth, but better lung function.

**What do these findings mean?:**

- Policies that support healthy ageing should prioritise maintaining functional ability and independence, particularly among socioeconomically disadvantaged populations, while recognising the different functional health needs of women and men.
- Socioeconomic inequalities in functional ageing are not fully explained by health conditions or behavioural risk factors, suggesting that both individual-level interventions and broader structural measures are needed to reduce inequalities.
- We could not examine all potential contributing factors, such as access to all health and social care services, and we focussed on socioeconomic conditions in adulthood rather than experiences across the life course.

## Abstract 3

In a longitudinal cohort study, Stephanie Schrempft and colleagues find that socioeconomic inequalities in functional ageing are evident among older adults in Canada and England, although the magnitude of these inequalities differs between the two countries, despite their similar universal healthcare systems and levels of economic development.

## Introduction

As the global population ages, there is a need to reduce the risk of disease and disability burden [1,2]. Physical functioning declines with age due to age-related physiological changes. However, research has shown that there is substantial variation in the rate of physiological decline among individuals of the same age [3,4]. Identifying the determinants of diverging ageing trajectories can therefore inform policymakers on how to extend healthy lifespan. Functional outcomes, such as mobility and ability to perform activities of daily living, are important indicators of healthy ageing [5,6] because they reflect the degree to which an individual is independent, and they are strongly associated with hospitalisation [7], nursing home placement [8], and quality of life [6].

Socioeconomic conditions are among the strongest predictors of health outcomes, including disease risk, mortality risk, and functional decline [9–11]. Functional disability is more prevalent in lower-income than higher-income countries [12]; and both neighbourhood-level and individual-level markers of socioeconomic disadvantage, such as living in a deprived area or having low wealth, substantially increase the risk of poor physical functioning [13,14]. There is evidence that recent cohorts in some higher-income countries are ageing better, but this is apparent only among the wealthiest groups, leading to a widening of functional health inequalities [15,16]. Potential contributing factors include exposure to healthier environments and easier access to high-quality healthcare services among wealthier individuals [17,18], as well as non-material factors, such as education, health behaviours, and coping resources [19,20].

Although socioeconomic inequalities in health are apparent across time and place, their magnitude is highly variable, particularly for inequalities in mortality [21–24]. For example, Nordic countries and continental Europe, which have relatively small resource inequalities, have substantially smaller inequalities in mortality for preventable causes compared to Central & Eastern Europe, which have large resource inequalities [22–24]. However, Southern European countries, which also have large resource inequalities, have smaller inequalities in mortality for preventable causes compared to Northern European countries [22–24]—a finding that has been attributed to differences in cultural norms and health behaviours [19,20]. Outside of Europe, health and mortality inequalities are greater in the United States (US) than in European countries and Canada, which have been partly attributed to differences in public healthcare and social welfare policy [18,25]. Taken together, these findings indicate that socioeconomic inequalities in health strongly depend on the context and highlight the importance of understanding when and why they occur.

Cross-country variation in functional health inequalities has been reported in some previous studies [10,26]; for example, socioeconomic inequalities in walking speed were greater in the US than in Europe [10]. However, there is a dearth of cross-country research examining the relationship between socioeconomic conditions and functional ageing trajectories. Whether socioeconomic inequalities in trajectories of functional ageing vary between countries provides insight into how diverse institutional, economic, and social contexts shape the ageing process, and is highly relevant to policies aimed at reducing health disparities [22,27]. By comparing countries with universal, predominantly tax-funded healthcare systems, similar levels of economic development, and comparable income inequality, observed differences in socioeconomic gradients in functional ageing cannot be attributed to differential access to universal healthcare or to broad differences in national wealth. This isolates variation in other structural and policy factors as explanatory candidates. As wealthy developed nations, England and Canada share many similarities in living standards and styles. Both countries are considered to be ‘liberal’ welfare states, and both have single-payer universal healthcare systems. The countries also differ in important ways. For example, Canada is geographically larger than England and has a federal governance structure, while England has a unitary governance structure and is very densely populated. These factors can impact the organisation and delivery of health care and social welfare programs, which may contribute to cross-country differences in health inequalities. Intersectionality has been suggested as a promising way to further advance health inequalities research because it reflects the fact that people embody multiple social characteristics (such as gender, race, and socioeconomic position) simultaneously [28]. However, few studies have incorporated an intersectionality approach in the context of ageing.

The present study, therefore, has two specific aims. First, to examine whether and how socioeconomic conditions are associated with functional ageing trajectories in two countries with universal healthcare systems, similar levels of economic development, and comparable income inequality: England and Canada. Second, to assess whether sex, race, chronic conditions, weight status, health behaviours, and social connections mediate or modify the relationship between socioeconomic conditions and functional ageing trajectories, and whether these patterns differ between the two countries.

## Methods

This is a longitudinal comparative cohort study and is reported as per the Strengthening the Reporting of Observational Studies in Epidemiology (STROBE) guideline (S1 Checklist). The study objectives and primary analytical approach were specified a priori. Specific model parameterisations (including the inclusion of higher-order age terms and their interactions) were informed by assessment of model fit.

### Participants

This study used data from the English Longitudinal Study of Ageing (ELSA) and the Canadian Longitudinal Study on Aging (CLSA). ELSA is a panel study of people aged 50 years or older living in England who were recruited from the annual Health Survey for England [29]. Participants gave written informed consent, and the study protocol has been approved by the National Research Ethics Service. We used data from waves 6 (2012–2013), 8 (2016–2017), 9 (2018–2019), and 10 (2021–2023; 11 years of follow-up) to allow for a closer comparison with CLSA in terms of timing (2012–2021; 9 years of follow-up). Lung function and chair stands were only assessed at waves 2 (2004–2005), 4 (2008–2009), and 6, so we used this data for these outcomes. There were 8511 participants aged 50–85 years with data on wealth and primary covariates (age, sex, and race) at baseline (wave 6). All participants had information on at least one functional outcome (maximum analytical sample for any single outcome = 8510; minimum analytical sample = 5525; see S1 Table for *N* of each analytical sample). For each analytical sample, 70–80% had follow-up outcome data (on one or more time points; see S1 Table). In ELSA, mortality data are not available for onward sharing after wave 6, limiting direct assessment of mortality-related attrition in this cohort. Of the maximum analytic sample, 2167 participants (25%) were missing information on one or more of the secondary covariates at baseline (primarily due to missing BMI data; see S2 Table for descriptives of all covariates and functional outcomes at each time point). CLSA is a panel study of people aged 45–85 years when recruited and living within 25–50 km of a data collection site [30]. CLSA comprises two cohorts - the Comprehensive cohort (*n* = 30,097) and the Tracking cohort (*n* = 21,241), described in detail elsewhere [30]. The study protocol was approved by the research ethics board at each research site with the coordination of the McMaster Research Ethics Board. The present study was approved by the University of British Columbia Behavioural Research Ethics Board (UBC BREB, approval number H23-02848). We used data from each of the three data collection waves of the Comprehensive Cohort: baseline (2012–2015), first follow-up (2015–2018), and second follow-up (2018–2021). We included participants aged 50 years and above to be consistent with ELSA. There were 22,605 participants aged 50–85 years with data on wealth and primary covariates (age, sex, and race) at baseline. All participants had information on at least one functional outcome (maximum analytical sample for any single outcome = 22,604; minimum analytical sample = 14,402; see S1 Table for *N* of each analytical sample). For each analytical sample, 82–96% had follow-up outcome data (on one or more time points; see S1 Table). Among those without any follow-up data (*N* = 2695; 10% of the total maximum sample), 1,076 had died, and the remaining 1,619 were lost to follow-up for other reasons. Of the maximum analytic sample, 634 participants (3%) were missing information on one or more of the secondary covariates at baseline (see S3 Table for descriptives of all covariates and functional outcomes at each time point).

### Measures

See S4 Table for an overview and comparison of the study measures in the two cohorts, which are described below.

#### Socioeconomic factors.

Socioeconomic factors of interest were assessed at baseline and included as time invariant factors. Total household wealth was our primary socioeconomic measure because it better captures the material resources available to older adults, as well as their permanent socioeconomic position, than other socioeconomic indicators do [31,32]. In ELSA, total non-pension household wealth was measured as net total wealth of a benefit unit (a single adult or a married or cohabiting couple and any dependent children), comprising the sum of savings and investments after financial debt has been subtracted. To improve comparability across cohorts, the continuous wealth measure in ELSA was categorised using cohort-specific quantiles to match the distribution of the CLSA wealth categories, thereby capturing relative socioeconomic position within each cohort. The mean wealth at baseline (2012–2013) in the four quantiles was £22,164, £138,045, £340,233, and £1,447,431, for the lowest to highest, respectively. In CLSA, wealth was the total value of savings and investments (of a single adult or a couple), categorised as less than $50,000, $50,000 to less than $100,000, $100,000 to less than $1 million, and $1 million or more. As the measure of wealth in ELSA is more comprehensive than that in CLSA, we included education level as the socioeconomic indicator in Supplementary analyses. In each cohort, education level was categorised as less than secondary school, secondary school, or post-secondary education. For each socioeconomic indicator, the highest category was the reference group (0).

#### Functional health outcomes.

We included both objective and subjective measures of functional health. Objective measures included gait speed, grip strength, chair stands, and lung function, collected by nurses either during home visits (ELSA) or at designated data collection sites (CLSA). Subjective measures included sensory function, ability to perform basic and instrumental activities of daily living, and mobility, obtained through computer-assisted personal interviews conducted by trained interviewers in participants’ homes in both cohorts.

Gait speed (m/s) was assessed with two 8-foot walking tests (one 4-metre test in CLSA) from a standing start. Individuals who had health conditions or disabilities that prevented walking were not eligible for the test. In ELSA, the test was carried out among participants aged 60 years or older, so, for comparison purposes, we also restricted the analysis to this age range in CLSA. Grip strength (kg) was assessed using a hand dynamometer. We took the maximum value across three trials using the dominant hand. For the chair stands, participants were asked to stand up and sit down on a firm chair without using their arms as quickly as they could for five rises. We used the average time taken for one chair rise (total time to complete the 5 chair stands (in seconds)/5). Forced expiratory volume (FEV, litres) was assessed by spirometry and used as a measure of lung function. We took the highest reading across three successful measures.

Sensory function was assessed by asking participants to rate their hearing and eyesight on a 5-point scale (1 = excellent, 5 = poor or non-existent). Self-reported difficulties in activities of daily living were assessed using a list of 6 basic activities (ADL), and 7 instrumental activities (IADL). ADL included dressing, walking across a room, bathing or showering, eating, getting in or out of bed, and using the toilet. IADL included using a map to get around in a strange place, preparing a hot meal, shopping for groceries, making telephone calls, taking medications, doing work around the house or garden, and managing money. An ADL score (range 0–6) and an IADL score (range 0–7) were calculated by summing the number of self-reported limitations and square root transformed due to the skewed distribution. In ELSA, self-reported mobility impairment was additionally available and included. This was measured as any difficulty performing 10 activities involving leg mobility and arm function (e.g., walking 100 yards, reaching or extending your arms above shoulder level). The maximum score was 10, with higher scores indicating more mobility impairment. In CLSA, timed up and go test [33] data were additionally available and included as performance time in seconds.

#### Covariates.

Primary covariates included sex (self-reported as male or female; information on gender was not available) and race (white or nonwhite). Race was categorised as white or nonwhite using participants’ self-reported ethnic group to facilitate harmonisation across cohorts and because the number of participants in individual racial/ethnic minority groups was insufficient to permit stable estimates. Height was also included as a primary covariate for gait speed and grip strength analyses, consistent with the literature [34,35]. Additional covariates included the number of chronic health conditions (0, 1, 2, 3, or more), based on self-reports of physician diagnosis (e.g., ‘has a doctor ever told you that you have heart disease?’ See S4 Table for the list of included conditions), weight status (obesity, overweight, healthy weight, and underweight), smoking (current smoker, former smoker, and nonsmoker), frequency of alcohol consumption (not in the past 12 months, less than once a month, once or twice a month, at least once a week, and almost every day), and marital status (married or in a partnership, other). A social isolation index was created by assigning one point if the respondent had less than monthly contact with each of the children, other family members, and friends, and if they did not participate in social clubs or groups [36]. Social isolation was positively skewed, so we defined the top quintile (a score ≥2) as being isolated, as done previously [36]. Loneliness was assessed using the 3-item University of California, Los Angeles (UCLA-3) Loneliness Scale [37] in ELSA and using an item from the 10-item Center for Epidemiologic Studies Depression Scale (CES-D-10) [38] in CLSA (‘In the past week, how often have you feel lonely?’). Responses in each cohort were categorised so that those who reported feeling lonely some of the time were identified as being lonely (versus not lonely). Time-varying covariates (assessed at each wave) were the number of chronic conditions, weight status, smoking, alcohol consumption, marital status, social isolation, and loneliness.

### Statistical analysis

Mixed-effects models, with a random intercept and a random linear slope, were used to calculate the fixed slope for the sample, as well as participants’ personal slopes (change in functioning per year of age) across the years of follow-up. To allow for potential nonlinear age effects, we included a quadratic age term in the fixed slope estimation in each model. We did not include an interaction term between quadratic age and wealth or the covariates as this worsened model fit (as indicated by higher Bayesian information criterion (BIC) values) across outcomes, and most of these interaction terms were not statistically supported.

Mixed-effects models do not require an equal number of observations from all participants and enable use of data for those with at least one valid outcome measure, thereby avoiding the need to restrict analyses to participants with complete or near-complete follow-up. All respondents contribute to the intercept term, while those with at least two valid outcome measures contribute to the slope term. Participants therefore contribute data only while they are observed, and once they are no longer present in the study because of death or drop out, they no longer contribute further observations. To account for potential selection bias due to nonrandom attrition, logistic regression was used to estimate the probability of remaining under observation at follow-up ((having follow-up data in the last 2 waves in ELSA, and in the last wave in CLSA) as a function of age, sex, race, education level, and chronic conditions). Participants who died or were otherwise lost to follow-up were considered not observed at follow-up. Inverse probability weights were defined as the inverse of this predicted probability and were applied in all models.

For the mixed-effects models, we aligned the direction of all functional outcomes so that higher values consistently reflected poorer functioning. For measures where higher values originally indicated better function (gait speed, grip strength, and lung function), we reversed the direction by multiplying by −1. This transformation does not affect model fit or statistical inference but ensures consistent interpretation across outcomes. Statistical analyses were conducted using Stata version 16 (StataCorp LLC, College Station, TX, USA) and R version 4.1.0 (R Foundation for Statistical Computing, Vienna, Austria).

#### Main analyses.

To examine whether the fixed (average) slope varied by wealth status, each model included age (in years, centred at 65 years) and wealth, as well as an interaction term between wealth and age (wealth × age). A significant interaction effect indicates that the average rate of change in functioning varies by wealth status. Covariates in the first set of models were birth cohort (year of birth), sex, race, and height (the latter for gait speed and grip strength analyses only). A second set of models further adjusted for chronic conditions, weight status, smoking, and alcohol consumption, and a third set further adjusted for marital status, social isolation, and loneliness. Missing data on covariates were imputed through multivariate imputation by chained equations (20 imputed data sets) [39].

We computed the number of years of functioning lost (YFL) from the minimally-adjusted mixed-effects model predictions of each functional outcome, with confidence intervals determined through 5,000 bootstrap samples. We computed the YFL associated with having the least wealth by predicting the chronological age of those with the most wealth equivalent to the level of functioning at age 60 (or 75) of those with the least wealth. To test whether associations between wealth and functioning varied by sex or race, we re-ran the mixed-effects models additionally including the interaction terms wealth × sex, wealth × race, wealth × sex × age, and wealth × race × age.

#### Supplementary analyses.

Analyses were performed separately within each cohort. In Supplementary analyses, we performed a pooled analysis combining data from ELSA and CLSA to further test whether there were cohort differences in the associations between wealth and the level of and mean change in functioning. We included a dummy variable for country, and an interaction between this variable and all other explanatory variables. Second, we repeated the models (within each cohort) using education level as the indicator of socioeconomic disadvantage. As different socioeconomic factors represent distinct constructs that may have different roles in development and ageing [40–42], wealth and education level were examined in separate models. Household wealth and education were moderately correlated (r = 0.20 (CLSA) and 0.32 (ELSA), p < 0.001), indicating that the constructs are not redundant measures of socioeconomic position. In an additional supplementary analysis conducted in response to peer review, we examined disease categories as covariates (respiratory, muscoskeletal, neurological, mental, cardiovascular, diabetes, and cancer; see S4 Table for conditions included in each category) to assess the robustness of the primary findings.

No artificial intelligence tools were used in the preparation of the submitted manuscript. The authors retain full responsibility for the content of the manuscript.

## Results

Table 1 includes characteristics of the two cohorts at baseline. ELSA participants were aged 67 years on average, 55% were female, and 4% had nonwhite race; while CLSA participants were aged 64 years on average, 49% were female, and 3% had nonwhite race. Compared to ELSA participants, CLSA participants had a higher education level. A greater proportion of CLSA participants had diagnosed health conditions than ELSA participants did, but functional health outcomes were generally better among CLSA participants than ELSA participants.

**Table 1:** Baseline characteristics of participants in the two cohorts (statistics are mean (SD) or % (n)).

	Total	ELSA, 2012/13	CLSA, 2012/15	*p*-value
	*N* = 31,116	*N* = 8,511	*N* = 22,605	
Age (years)	64.8 (9.1)	66.7 (8.6)	64.1 (9.2)	<0.001
Sex				<0.001
Male	49.5 (15,394)	44.8 (3,816)	51.2 (11,578)	
Female	50.5 (15,722)	55.2 (4,695)	48.8 (11,027)	
Race				0.007
White	96.8 (30,123)	96.4 (8,202)	97.0 (21,921)	
Nonwhite	3.2 (993)	3.6 (309)	3.0 (684)	
Marital/partner status				0.23
Married/living with a partner	69.2 (21,539)	69.7 (5,936)	69.0 (15,603)	
Other	30.8 (9,571)	30.3 (2,575)	31.0 (6,996)	
Highest level of education				<0.001
Post-secondary degree/diploma	66.4 (20,634)	35.7 (3,033)	78.0 (17,601)	
Secondary school graduation	20.0 (6,229)	28.7 (2,437)	16.8 (3,792)	
Less than secondary school graduation	13.5 (4,206)	35.7 (3,032)	5.2 (1,174)	
Total household wealth^1^				1.00
Highest	11.6 (3,596)	11.5 (983)	11.6 (2,613)	
Higher	51.3 (15,977)	51.3 (4,364)	51.4 (11,613)	
Lower	15.5 (4,828)	15.6 (1,327)	15.5 (3,501)	
Lowest	21.6 (6,715)	21.6 (1,837)	21.6 (4,878)	
Chronic health conditions				<0.001
None	26.5 (8,228)	37.4 (3,185)	22.3 (5,043)	
One	30.8 (9,581)	30.5 (2,598)	30.9 (6,983)	
Two	24.4 (7,578)	21.0 (1,790)	25.6 (5,788)	
Three or more	18.3 (5,690)	11.0 (938)	21.1 (4,752)	
Weight status				<0.001
Healthy weight	27.5 (8,157)	25.5 (1,790)	28.2 (6,367)	
Underweight	0.7 (213)	0.9 (60)	0.7 (153)	
Overweight	41.2 (12,208)	41.4 (2,908)	41.2 (9,300)	
Obesity	30.5 (9,035)	32.3 (2,266)	30.0 (6,769)	
Smoking status				<0.001
Never smoked	44.0 (13,657)	37.6 (3,188)	46.3 (10,469)	
Former smoker	46.5 (14,437)	49.7 (4,212)	45.2 (10,225)	
Current smoker	9.6 (2,979)	12.6 (1,069)	8.4 (1,910)	
Frequency of alcohol consumption				<0.001
Not in the past 12 months	11.8 (3,469)	13.2 (973)	11.3 (2,496)	
Less than once a month	12.9 (3,801)	16.4 (1,208)	11.7 (2,593)	
Once or twice a month	15.1 (4,467)	11.6 (857)	16.3 (3,610)	
At least once a week	41.6 (12,267)	37.9 (2,793)	42.8 (9,474)	
Almost every day	18.6 (5,488)	20.8 (1,536)	17.9 (3,952)	
Lonely				<0.001
No	75.9 (22,678)	79.0 (5,811)	74.9 (16,867)	
Yes	24.1 (7,207)	21.0 (1,544)	25.1 (5,663)	
Socially isolated				<0.001
No	65.7 (19,742)	40.6 (3,045)	74.1 (16,697)	
Yes	34.3 (10,289)	59.4 (4,450)	25.9 (5,839)	
Gait speed, metres/second	1.0 (0.2)	0.9 (0.3)	1.0 (0.2)	<0.001
Max grip strength, kilograms	34.2 (11.7)	30.9 (11.1)	35.3 (11.6)	<0.001
Average time for 1 chair rise, log seconds^2^	0.9 (0.3)	0.8 (0.3)	1.0 (0.3)	<0.001
Forced expiratory volume, litres^2^	2.7 (0.8)	2.3 (0.8)	2.7 (0.8)	<0.001
Eyesight rating, 1 = excellent, 5 = poor	2.3 (0.9)	2.5 (1.0)	2.2 (0.9)	<0.001
Hearing rating, 1 = excellent, 5 = poor	2.4 (1.0)	2.6 (1.1)	2.4 (1.0)	<0.001
Number of ADL difficulties, square root	0.2 (0.4)	0.2 (0.5)	0.1 (0.4)	<0.001
Number of IADL difficulties, square root	0.1 (0.4)	0.2 (0.6)	0.1 (0.3)	<0.001
Number of mobility difficulties	1.9 (2.6)	1.9 (2.6)		
Up and go time, log s	2.2 (0.2)	–	2.2 (0.2)	–

Abbreviations: ELSA = English Longitudinal Study of Ageing; CLSA = Canadian Longitudinal Study on Aging; ADL = basic activities of daily living; IADL = instrumental activities of daily living.

^1^From lowest to highest, wealth was categorised as less than $50,000, $50,000 to less than $100,000, $100,000 to less than $1 million, and $1 million or more in CLSA. In ELSA, wealth was categorised using cohort-specific quantiles to match the distribution of the CLSA wealth categories. The mean wealth at baseline (2012–2013) in the four quantiles was £22,164, £138,045, £340,233, and £1,447,431, for the lowest to highest, respectively.

^2^Baseline was wave 2 (2004/05) in ELSA.

**Fig 1:**
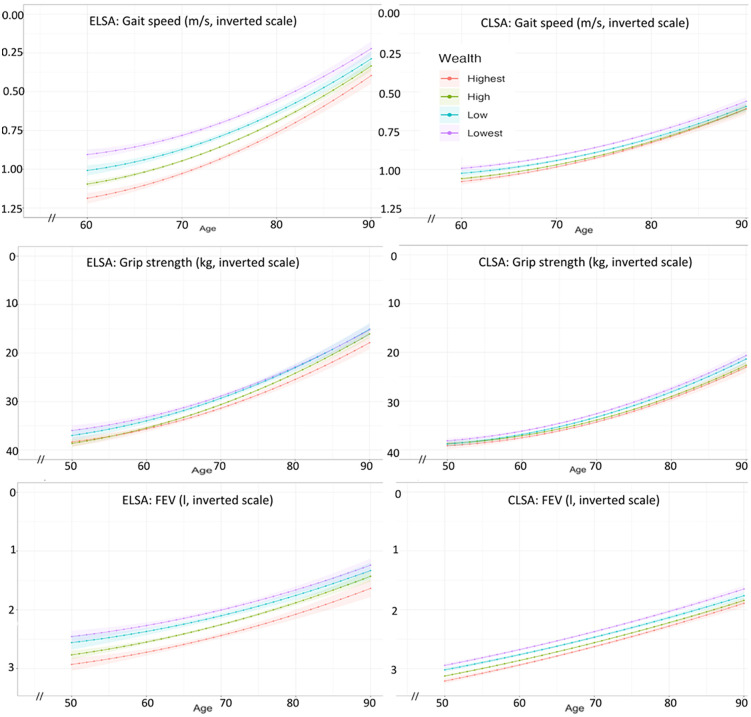
Associations between wealth and functional ageing trajectories for gait speed, grip strength, and lung function in ELSA and CLSA. Lines represent predicted trajectories estimated from the mixed-effects models. Note that the y axes have been inverted for consistency of presentation across outcomes. Mixed-effects models included age, age^2^, birth year, as well as sex, race, height (gait speed and grip strength only), wealth, and their interaction with linear age as covariates. Abbreviations: ELSA = English Longitudinal Study of Ageing; CLSA = Canadian Longitudinal Study on Aging; FEV = Forced Expiratory Volume.

**Fig 2:**
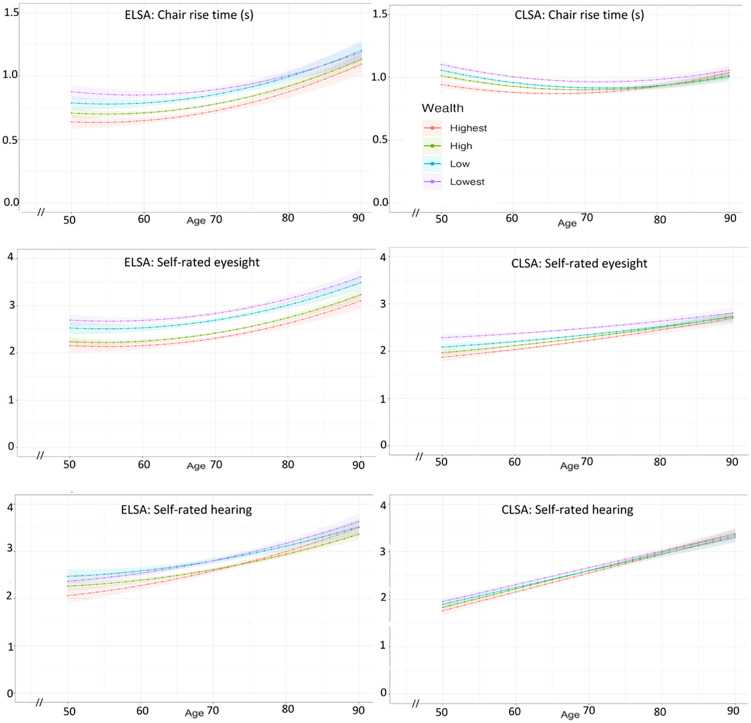
Associations between wealth and functional ageing trajectories for chair rise test performance, self-rated eyesight, and self-rated hearing in ELSA and CLSA. Lines represent predicted trajectories estimated from the mixed-effects models. Higher values indicate poorer functioning. Mixed-effects models included age, age^2^, birth year, as well as sex, race, wealth, and their interaction with linear age as covariates. Abbreviations: ELSA = English Longitudinal Study of Ageing; CLSA = Canadian Longitudinal Study on Aging.

**Fig 3:**
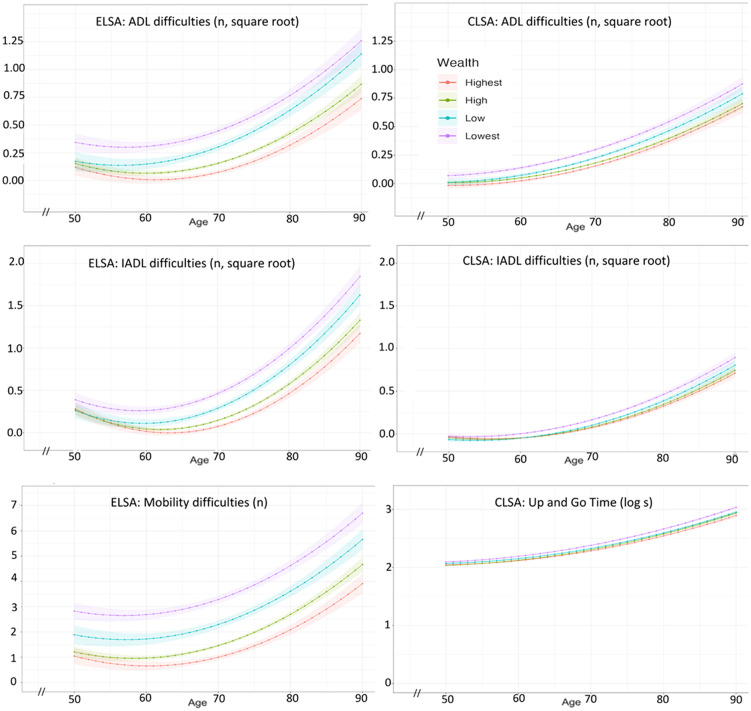
Associations between wealth and functional ageing trajectories for basic and instrumental activities of daily living (ADL and IADL) and mobility in ELSA and CLSA. Lines represent predicted trajectories estimated from the mixed-effects models. Higher values indicate poorer functioning. Mixed-effects models included age, age^2^, birth year, as well as sex, race, wealth, and their interaction with linear age as covariates. Abbreviations: ELSA = English Longitudinal Study of Ageing; CLSA = Canadian Longitudinal Study on Aging; ADL = basic activities of daily living; IADL = instrumental activities of daily living.

Figs 1–3 show the predicted values of functioning across age by wealth category, estimated from the mixed-effects models. The model estimates are shown in S5 Table and S6 Table. In each cohort, less wealth was associated with a poorer level of functioning across outcomes, including slower gait speed, weaker grip strength, slower chair rise time, poorer lung function, poorer eyesight, poorer hearing, more difficulties in ADL and IADL, more mobility difficulties (ELSA), and slower up and go time (CLSA) at baseline (intercept). There was also evidence that the relationship between age and several of the functional outcomes varied by wealth. Compared with having the most wealth, having the least wealth was associated with a greater deterioration in the ability to perform ADL (ELSA: b = 0.007, 95% CI [0.003, 0.012], *p* = 0.003; CLSA: b = 0.003, 95% CI [0.001, 0.005], *p* = 0.006) and IADL (ELSA: b = 0.014, 95% CI [0.009, 0.019], *p* < 0.001; CLSA: b = 0.004, 95% CI [0.003, 0.006], *p* < 0.001) with age after adjustment for primary covariates. Having the least wealth was also associated with a greater decline in mobility in ELSA (b = 0.025, 95% CI [0.006, 0.045], *p* = 0.011) and a greater decline in timed up and go performance in CLSA (b = 0.002, 95% CI [0.001, 0.004], *p* = 0.001). In ELSA, having the most wealth was associated with a greater decline in gait speed than having the least wealth (b = -0.004, 95% CI [-0.007, -0.001], *p* = 0.021), although the level of gait speed did not reach that of those with the least wealth. In CLSA, having the most wealth was associated with a greater decline in chair rise performance (b = -0.003, 95% CI [-0.005, -0.002], *p* < 0.001) and self-rated eyesight (b = -0.008, 95% CI [-0.012, -0.003], *p* = 0.003) than having the least wealth. The pattern of results was similar when using education level as the socioeconomic indicator (see S7 Table).

These associations were attenuated but generally persisted after additional adjustment for the number of chronic conditions, weight status, health behaviours (smoking and alcohol consumption; model 2), and social connections (marital status, social isolation, and loneliness; model 3). For the level of functioning, adjustment attenuated the estimates (lowest versus highest wealth categories) by 0% (grip strength) to 22% (ADL difficulties) in ELSA (median: 15%); and by 13% (grip strength) to 38% (IADL difficulties) in CLSA (median: 25%). For the change in functioning, adjustment attenuated the estimates (lowest versus highest wealth categories) by 7% (IADL difficulties) to 25% (gait speed) in ELSA (but strengthened the association for ADL and mobility difficulties); and by 0% (chair rise and timed up and go test) to 38% (self-rated eyesight) in CLSA (median: 15.5%). See S5 and S6 Tables for all estimates across the set of models. When adjusting for the type of chronic condition (instead of the number), the pattern of results was unchanged (see S8 Table).

**Fig 4:**
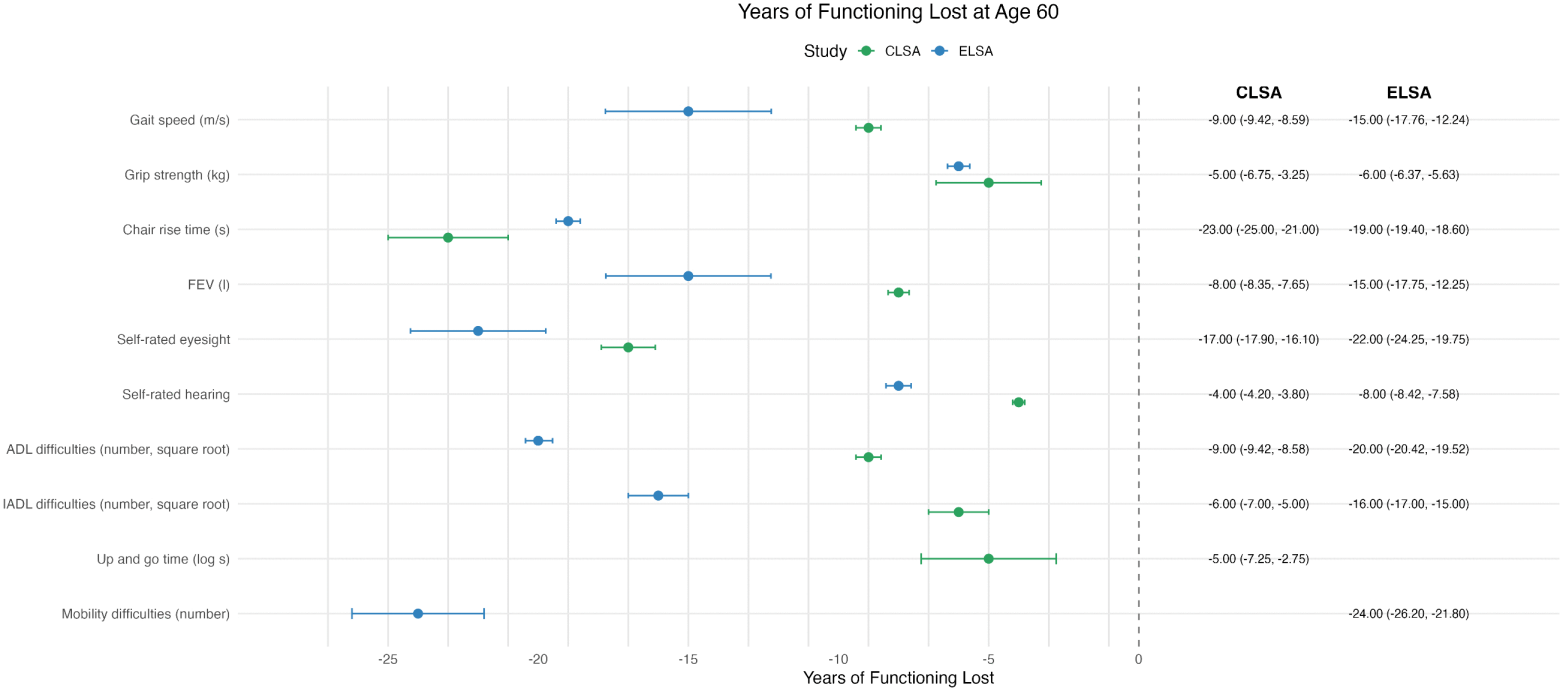
Years of functioning lost (and 95% confidence intervals) associated with having the least wealth at age 60 for each functional outcome in ELSA (*N* range = 5,525–8,510) and CLSA (*N* range = 14,402–22,604). Abbreviations: ELSA = English Longitudinal Study of Ageing; CLSA = Canadian Longitudinal Study on Aging; FEV = Forced Expiratory Volume; ADL = basic activities of daily living; IADL = instrumental activities of daily living.

**Fig 5:**
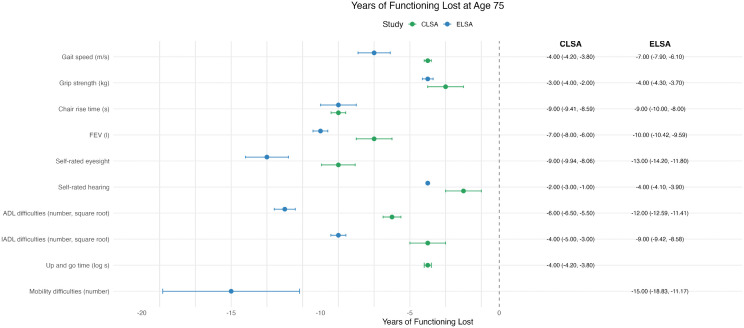
Years of functioning lost (and 95% confidence intervals) associated with having the least wealth at age 75 years for each functional outcome in ELSA (*N* range = 5,525–8,510) and CLSA (*N* range = 14,402–22,604). Abbreviations: ELSA = English Longitudinal Study of Ageing; CLSA = Canadian Longitudinal Study on Aging; FEV = Forced Expiratory Volume; ADL = basic activities of daily living; IADL = instrumental activities of daily living.

Figs 4 and 5 show the YFL by age 60 (and age 75) associated with low wealth. At age 60, cohort differences were particularly evident for gait speed and difficulties performing ADL and IADL. In ELSA, on average, a 60-year-old with the least wealth had the same gait speed as a 75-year-old with the most wealth (YFL = 15, 95% CI [12.2, 17.8]), the same difficulties with ADL as an 80-year-old with the most wealth (YFL = 20, 95% CI [19.5, 20.4]), and the same difficulties with IADL as a 76-year-old with the most wealth (YFL = 16, 95% CI [15.0, 17.0]). In CLSA, on average, the corresponding differences were YFL = 9 (95% CI [8.6, 9.4]) for gait speed, YFL = 9 (95% CI [8.6, 9.4]) for ADL, and YFL = 6 (95% CI [5.0, 7.0]) for IADL. At age 75, differences between cohorts were smaller but still notable. In ELSA, on average, the difference between 75-year-olds with the least versus the most wealth was YFL = 12 for ADL (95% CI [11.4, 12.6]), while in CLSA it was YFL = 6 (95% CI [5.5, 6.5]).

The results of the pooled analyses (see S9 Table) confirmed that wealth inequalities in the level of functioning were generally larger in ELSA than in CLSA. CLSA participants with the least wealth had a better level of functioning than ELSA participants with the least wealth, including faster gait speed, stronger grip strength, higher FEV, better self-rated eyesight and hearing, and fewer difficulties performing ADL and IADL. The results also confirmed that wealth inequalities in functioning trajectories differed in ELSA and CLSA. CLSA participants with the least wealth had less decline in ADL and IADL with age than ELSA participants with the least wealth.

The association between wealth and some functional outcomes varied by sex within each cohort. In CLSA, there was evidence that women with the least wealth had more ADL difficulties (b = 0.089, 95% CI [0.055, 0.124], *p* < 0.001), more IADL difficulties (b = 0.050, 95% CI [0.024, 0.076], *p* < 0.001), and poorer timed up and go performance (b = 0.051, 95% CI [0.028, 0.073], *p* < 0.001) than men with the least wealth. In ELSA, there was some evidence that women with the least wealth had poorer hearing than men with the least wealth (b = 0.342, 95% CI [0.173, 0.512], *p* < 0.001). Conversely, women with the least wealth had better lung function than men with the least wealth in each cohort (ELSA: b = -0.210, 95% CI [-0.334, -0.086], *p* = 0.001; CLSA: b = -0.100, 95% CI [-0.163, -0.038], *p* = 0.002), as well as stronger grip strength in CLSA (b = -2.053, 95% CI [-2.828, -1.277], *p* < 0.001). There was little evidence that associations between wealth and functional decline varied by sex or race. See S10 Table for all interaction effects.

## Discussion

In this longitudinal cross-country comparison between England and Canada, we observed socioeconomic inequalities in the level of functioning in both countries across multiple outcomes. Socioeconomic inequalities in the level of functioning were generally larger in England than in Canada, especially for gait speed, ADL, and IADL. Moreover, we observed socioeconomic inequalities in functional ageing trajectories in both countries, such that less wealth was associated with greater decline in mobility and in the ability to perform ADL and IADL with age. Wealth inequalities in functional health and ageing were attenuated but persisted after adjustment for common risk factors, including health behaviours, weight status, chronic conditions, and social connections. There was some evidence that associations between wealth and functioning were modified by sex, such that women with the least wealth experienced greater difficulties with ADL, IADL, poorer mobility (timed up and go performance), and worse hearing than men with the least wealth, but better lung function.

Socioeconomic inequalities in functional ageing trajectories have previously been reported, with several studies finding that socioeconomic disadvantage is associated with greater functional decline with age, as indicated by more difficulties with mobility, ADL, and IADL [43,44]. While we also observed an association between socioeconomic disadvantage and greater decline in mobility, ADL, and IADL (in both cohorts), having more wealth was associated with a faster rate of decline in other functional outcomes, including gait speed, chair rise performance, and self-rated eyesight. Several other studies have also found that more advantaged individuals experience a faster rate of decline in functional outcomes such as gait speed [45] and cognitive function [46]. These findings can be explained in the context of the age-as-leveller perspective according to which social inequalities diminish in later life due to universal health decline and/or selective mortality [47]. In this sense, wealthier individuals have a higher level of functioning than their less wealthy counterparts, but they experience a ‘catch up’ when age-related physiological decline takes over. Nevertheless, we observed that the level of functioning in wealthier individuals did not reach that of those with the least wealth across most functional outcomes, which is more in line with the view that socioeconomic disadvantages accumulated in life continue to impact individuals’ health and well-being as they age [48]. Research suggests that the widening and narrowing of socioeconomic inequalities in health with age are not mutually exclusive, and depend on the measure of health used, the ages of the populations studied [49], and the focus on individual-level change versus change at the aggregate level [50]. Our study used an outcome-wide approach, and we chose to focus on the mean (fixed) effects rather than individual slopes. Further insights into socioeconomic impacts on functional health could be gained using an aggregate functional ageing measure similar to the updated Pace of Ageing measure [4].

The associations between wealth and functioning were attenuated but persisted across most outcomes when adjusting for chronic health conditions, weight status, smoking, alcohol consumption, social isolation, and loneliness. These findings are consistent with previous research showing that behavioural and social network factors, while important, only partially mediate socioeconomic inequalities in health and ageing [51–53]. Similarly, although chronic conditions are associated with poorer functioning, they do not fully explain socioeconomic inequalities [54,55]. This pattern is consistent with the notion that inequalities in health and ageing are the result of broader structural and cumulative processes, not only differences in individual risk factors or diagnosed morbidity [56,57]. Structural factors that are likely relevant and warrant further investigation include access to timely, high-quality, and continuous healthcare, rehabilitation, and assistive support [58], as well as specific occupational and environmental exposures [17], which may differentially affect functional outcomes. Both England and Canada permit supplementary private insurance and access to privately funded healthcare services. These factors may contribute to socioeconomic inequalities in functional health, as wealthier individuals may be able to obtain more timely and comprehensive healthcare services than those relying solely on the public system. Psychosocial factors beyond social isolation and loneliness, such as chronic stress exposure and perceived control [59,60], represent additional pathways linking structural conditions to functioning and should be examined in further research.

Socioeconomic inequalities in the level of functioning were apparent in both cohorts but differed in magnitude. As shown by the YFL analyses, differences between cohorts were particularly notable for gait speed and difficulties performing ADL and IADL. Gait speed and ability to perform ADL and IADL are key indicators of overall physiological decline and advanced ageing as both rely on multiple body systems. Slower gait speed and ADL and IADL difficulties predict a shorter lifespan [61,62], and reflect accelerated biological ageing, as well as cognitive decline [4,63]. The finding that disparities in markers of overall health status were larger in England than Canada could be attributed to various factors, including differences in healthcare access and quality, social welfare, including housing access and quality, as well as overall quality of life, and health behaviours. Life expectancy and life disparity are similar in England and Canada. However, the World Health Organisation ranks Canada among the top 10 of 191 countries and third among the 11 countries included in the Commonwealth Fund Report for health-adjusted life expectancy (at 72.3 versus 71.4 life-years in the United Kingdom (UK), and 69.1 life-years in the US) [64]. Analyses from the Global Burden of Disease Study also showed that Canada was in the top 10% of the 195 countries compared on the Healthcare Access and Quality Index, ranking above the UK [65]. Although Canada has a high rate of common chronic diseases (including hypertension, heart disease, diabetes, and lung conditions) [66], research indicates that Canadians with cardiovascular risk factors are more likely to be aware of their conditions, are more likely to be receiving treatment, and exhibit higher control rates compared with peers in the US or the UK [67]. Canada also scores higher than the UK on the Organisation for Economic Co-operation and Development (OECD) Better Life Index, which includes measures of material living conditions, environmental quality, social relationships, and quality of life [68]. A greater proportion of individuals’ disposable income is spent on housing costs in the UK than in other English-speaking countries, such as Canada [69], and, while the UK has one of the biggest social housing stocks in the world, waiting lists are proportionately longer than elsewhere in the anglosphere [69]. Moreover, English Housing Survey data indicate that a significant proportion of social houses have damp problems and category 1 hazards [70]. The UK and Canada have similar levels of income inequality overall, but Canada has a much higher degree of social mobility, meaning that it is easier for individuals to move between income brackets than in the UK [71].

There was some evidence that sex moderated associations between wealth and the level of functioning, such that women with less wealth experienced greater difficulties with ADL and IADL, poorer mobility (timed up and go performance), and worse hearing than men with less wealth, but better lung function. Taken together, these findings are consistent with previous research showing that socioeconomic gradients in health and functioning vary by sex, with the direction and magnitude of inequalities differing across health domains. In line with our findings, previous studies have shown that, while mortality rates are lower among women, women report greater functional limitations than men [72], and these differences are partly explained by socioeconomic factors [73]. Moreover, there is evidence that socioeconomic inequalities in respiratory outcomes are stronger among men, independent of smoking status [74]. Collectively, this pattern aligns with the well-established finding that women tend to live longer but experience a greater burden of disability, and that addressing socioeconomic inequalities can alleviate some of this burden. However, there was little evidence that sex moderated the association between wealth and functional ageing trajectories. While there is limited research examining how sex and socioeconomic factors interact to shape ageing trajectories, a recent study found that sex differences in physiological ageing are attenuated among highly educated women [75], while another found that early-life socioeconomic disadvantage was more strongly associated with cognitive decline in women than in men [76]. Interaction effects between sex and exposure to adversity depend both on the exposure (type and timing) and the specific health outcome considered [77], which likely explains the discrepancy between these and our study findings. Our finding that sex moderated the association between wealth and the level of functioning, but not functional ageing trajectories, could reflect cumulative disadvantages that are already established in later life. For example, women—especially those in low-income households—often have the greatest caregiving responsibilities, which limits their time and resources to access healthcare and maintain their own health [78,79]. In addition, because women—particularly single mothers and older women—are overrepresented in low-income households [80], they may be more likely to live in substandard housing and neighbourhoods [81], which could contribute to difficulties with ADL, IADL, and mobility. In contrast, among men, poorer lung function in lower-wealth groups may reflect greater cumulative exposure to occupational hazards—such as dust, chemicals, and physically demanding work—particularly in mid-life [82,83]. These exposures, which are more common in lower socioeconomic positions, may contribute to long-term respiratory impairment.

Compared with white individuals, nonwhite individuals performed worse on several of the functional measures, including slower gait speed, lower lung function (ELSA), weaker grip strength, poorer eyesight, and slower timed up and go performance (CLSA). However, race did not modify associations between wealth and functional ageing. This finding should be interpreted with caution, as nonwhite individuals are underrepresented in both cohorts, limiting statistical power to detect effect modification. The racial composition of older adults in England and Canada also reflects earlier birth cohorts, although both countries have become considerably more diverse in recent decades. Consequently, caution is warranted when generalising these findings to future cohorts of older adults, particularly with respect to the intersection of socioeconomic conditions and race. Earlier research in the US found that, compared to white adults, black adults experience a growing disadvantage in functional health over time until the oldest ages [84].

Study strengths include the use of data from two large population-based cohorts, the longitudinal design, assessment of multiple objective and subjective measures of functioning, adjustment for key covariates, and evaluation of whether associations between wealth and functional outcomes differed by sex and race. Gait speed was measured with a standard walking test in each cohort and expressed in standardised units (metres per second). However, we acknowledge that, while the tests in each cohort used a static start, they differed in terms of the number of tests (two 8-foot tests in ELSA, one 4-metre test in CLSA). These methodological differences may influence the comparability of the results between cohorts. While we chose an outcome-wide approach, further insights into ageing trajectories across cohorts will be gained using validated aggregate longitudinal measures of ageing, such as the recently updated Pace of Ageing measure. We did not consider all potential interactions, such as interactions between sex and race in associations between socioeconomic conditions and functional ageing trajectories, which could be the focus of future studies. An additional limitation is the underrepresentation of nonwhite participants in both cohorts, which limited our ability to examine racial and ethnic heterogeneity, and may reduce the generalisability of our findings to increasingly diverse older populations. Our analysis compared two liberal welfare states, which has the advantage of isolating subtler sources of variation that would be harder to detect in broader cross-regime comparisons. Comparing cohorts in other countries with different welfare models will provide insights into the role of broader institutional contexts in diverging ageing trajectories. However, such comparisons remain challenging due to the limited availability of harmonised longitudinal datasets with sufficient follow-up and comparable socioeconomic measures across these settings. Although we examined the contribution of important factors related to the ageing process, we could not examine all potential factors contributing to the observed associations. Access to general practitioner (GP) services, dentists, other health and personal care, as well as time on waiting lists (and move to private healthcare) have been introduced in ELSA in the latest wave (11) and should be examined in future research. Future research should also examine the contribution of specific occupational and environmental exposures (which likely vary by functional outcome) to provide additional insights beyond the contributing factors examined in this study. Although wealth is the most precise measure of socioeconomic resources in older adulthood, we did not have the exact same measures of wealth in each cohort for more accurate comparison. However, when using education as the socioeconomic indicator (which was measured in the same way in each cohort), the direction of the associations was similar to that for wealth, and we could still see that associations were generally larger in ELSA than in CLSA. We are therefore assured that our conclusions are not driven by differences in measurement between the two cohorts. Although we used inverse probability weighting to account for nonrandom attrition (mortality and drop-out), socioeconomic gradients in functional decline may be conservatively estimated: socioeconomically disadvantaged individuals have higher mortality and exit the analytic sample earlier, which may attenuate observed inequalities among those remaining under observation. We focussed on measures of socioeconomic conditions in adulthood, but a life course approach would provide further insights, as childhood socioeconomic conditions have been associated with functional ageing trajectories beyond socioeconomic conditions in adulthood.

Wealth inequalities in functioning were observed in both countries across multiple outcomes, underscoring the need for ageing policy to prioritise functional ability and independence alongside survival and disease incidence. The persistence in wealth inequalities after adjustment for chronic health conditions, weight status, and behavioural risk factors further highlights the importance of addressing upstream determinants, alongside individual-level factors. Plausible policy levers include strengthening income security through more adequate public pensions and minimum income guarantees, reducing financial barriers to services that are not comprehensively covered within universal health systems—particularly rehabilitation, assistive technologies, and long-term care, improving housing quality standards, and enforcing occupational health protections to limit physical and mental strain across working life. Inequalities in functional health and ageing were observed for both wealth and education, supporting a life-course approach to addressing social inequalities. Early-life investments in education and childhood conditions, alongside mid-life interventions targeting job security and working conditions, and improved access to high-quality healthcare across the life course are ways in which functional inequalities can be reduced. As functional inequalities were generally larger in England than in Canada, strengthening social protection policies may be especially relevant in this context. For clinical and health-system decision-making, the results support more proactive identification and management of functional decline in socioeconomically disadvantaged populations. This could include routine screening for functional and sensory limitations in lower-wealth patients, prioritised access to preventive and rehabilitative services, and integration of social risk factors into care planning. The observed sex differences further indicate the need for targeted strategies, such as focussing on functional limitations in disadvantaged women and respiratory health in disadvantaged men. Taken together, the findings suggest that reducing inequalities in functional health and ageing will require a combination of universal high-quality healthcare and targeted interventions for disadvantaged groups, implemented across the life course.

In the context of population ageing, examining the determinants of healthy ageing trajectories is a research priority. In this longitudinal cross-country study, socioeconomic factors were associated with both the level of functioning as well as functional ageing trajectories in both cohorts, highlighting the importance of research into the underlying mechanisms as well as policies aimed at reducing social inequalities. Future studies should continue to apply an intersectional lens to better understand socioeconomic disparities in ageing.

## Supporting information

S1 Checklist Strengthening the Reporting of Observational Studies in Epidemiology (STROBE) guideline checklist. The STROBE checklist is best used in conjunction with this article (freely available on the websites of PLoS Medicine at http://www.plosmedicine.org/, Annals of Internal Medicine at http://www.annals.org/, and Epidemiology at http://www.epidem.com/). Information on the STROBE Initiative is available at www.strobe-statement.org.
(DOCX)

S1 Table Size of each analytical sample in the two cohorts. (DOCX)

S2 Table Descriptives of model covariates and functional outcomes at each time point in ELSA. (DOCX)

S3 Table Descriptives of model covariates and functional outcomes at each time point in CLSA. (DOCX)

S4 Table Comparison of the study measures in the two cohorts. (DOCX)

S5 Table Associations between wealth and the level of and change in functional health outcomes in ELSA. (DOCX)

S6 Table Associations between wealth and the level of and change in functional health outcomes in CLSA. (DOCX)

S7 Table Associations between education and the level of and change in functional health outcomes in the two cohorts. (DOCX)

S8 Table Associations between wealth and the level of and change in functional health outcomes when adjusting for disease categories. (DOCX)

S9 Table Associations between wealth and the level of and change in functional health outcomes in the pooled analysis. (DOCX)

S10 Table Effects of sex and race on the association between wealth and functional health outcomes in the two cohorts. (DOCX)

## Abbreviations

ADL: activities of daily living
BIC: Bayesian information criterion
CES-D-10: Center for Epidemiologic Studies Depression Scale
CLSA: Canadian Longitudinal Study on Aging
ELSA: English Longitudinal Study of Ageing
FEV: forced expiratory volume
GP: general practitioner
IADL: instrumental activities of daily living
OECD: Organisation for Economic Co-operation and Development
YFL: years of functioning lost.
