# Supplementary material for: Socioeconomic inequalities in functional ageing trajectories in England and Canada: A comparative longitudinal cohort study
Source: PLoS Med. 2026 Aug 13;23(8):e1004833. doi: 10.1371/journal.pmed.1004833 (PMC13472442; doi:10.1371/journal.pmed.1004833)
Supplement: Supplementary file 1 [file pmed.1004833.s001.docx]

STROBE Statement—checklist of items that should be included in reports of observational studies

|  | Item No. | Recommendation | Page  No. | Relevant text from manuscript |
| --- | --- | --- | --- | --- |
| **Title and abstract** | 1 | (*a*) Indicate the study’s design with a commonly used term in the title or the abstract | p. 1 (Title) | Socioeconomic inequalities in functional ageing trajectories in England and Canada: a comparative longitudinal cohort study |
|  |  | (*b*) Provide in the abstract an informative and balanced summary of what was done and what was found | p. 2 (Abstract) | See abstract |
| Introduction | | | |  |
| Background/rationale | 2 | Explain the scientific background and rationale for the investigation being reported | p. 6 (Introduction, paragraph 4) | Cross-country variation in functional health inequalities has been reported in some previous studies [10,26]; for example, socioeconomic inequalities in walking speed were greater in the United States than in Europe [10]. However, there is a dearth of cross-country research examining the relationship between socioeconomic conditions and functional ageing trajectories. Whether socioeconomic inequalities in trajectories of functional ageing vary between countries provides insight into how diverse institutional, economic, and social contexts shape the ageing process, and is highly relevant to policies aimed at reducing health disparities [22,27]. By comparing countries that are matched on key potential confounders, such as universal, predominantly tax-funded healthcare access, similar levels of economic development, and comparable income inequality, observed differences in socioeconomic gradients in functional ageing cannot be attributed to differential healthcare access or wealth. This isolates variation in other structural and policy factors as explanatory candidates. As wealthy developed nations, England and Canada share many similarities in living standards and styles. Both countries are considered to be ‘liberal’ welfare states, and both have single-payer universal healthcare systems. The countries also differ in important ways. For example, Canada is geographically larger than England and has a federal governance structure, while England has a unitary governance structure and is very densely populated. These factors can impact the organisation and delivery of health care and social welfare programs, which may contribute to cross-country differences in health inequalities. Intersectionality has been suggested as a promising way to further advance health inequalities research because it reflects the fact that people embody multiple social characteristics (such as gender, race, and socioeconomic position) simultaneously [28]. However, few studies have incorporated an intersectionality approach in the context of ageing. |
| Objectives | 3 | State specific objectives, including any prespecified hypotheses | p. 6 (Introduction, paragraph 5) | The present study therefore has two specific aims. First, to examine whether and how socioeconomic conditions are associated with functional ageing trajectories in two countries with universal healthcare systems, similar levels of economic development, and comparable income inequality: England (ELSA) and Canada (CLSA). Second, to assess whether sex, race, chronic conditions, weight status, health behaviours, and social connections explain or modify the relationship between socioeconomic conditions and functional aging trajectories, and whether these patterns differ between the two countries. |
| Methods | | | |  |
| Study design | 4 | Present key elements of study design early in the paper | p. 7 (Methods, paragraph 1) | This is a longitudinal comparative cohort study and is reported as per the Strengthening the Reporting of Observational Studies in Epidemiology (STROBE) guideline (S1 Checklist). The study objectives and primary analytical approach were specified a priori. Specific model parameterisations (including the inclusion of higher-order age terms and their interactions) were informed by assessment of model fit. |
| Setting | 5 | Describe the setting, locations, and relevant dates, including periods of recruitment, exposure, follow-up, and data collection | pp. 7-8 (Participants, paragraphs 1 and 2) | The English Longitudinal Study of Ageing (ELSA) is a panel study of people aged 50 years or older living in England (29). Participants were recruited from the annual Health Survey for England (29). Participants gave written informed consent, and the study protocol has been approved by the National Research Ethics Service. We used data from waves 6 (2012-2013), 8 (2016-2017), 9 (2018-2019), and 10 (2021-2023) to allow for a closer comparison with CLSA in terms of timing (2012-2021). Lung function and chair stands were only assessed at waves 2 (2004-2005), 4 (2008-2009), and 6, so we used this data for these outcomes. There were 8511 participants aged 50 to 85 years with data on wealth and primary covariates (age, sex, and race) at baseline (wave 6). All participants had information on at least one functional outcome (maximum analytical sample for any single outcome = 8510; minimum analytical sample = 5525; see S1 Table for N of each analytical sample). For each analytical sample, 70-80% had follow-up outcome data (on one or more time points; see S1 Table). In ELSA, mortality data is not available for onward sharing after wave 6, limiting direct assessment of mortality-related attrition in this cohort. Of the maximum analytic sample, 2167 participants (25%) were missing information on one or more of the secondary covariates at baseline (primarily due to missing BMI data; see S2 Table for descriptives of all covariates and functional outcomes at each time point).  The Canadian Longitudinal Study on Aging (CLSA) is a panel study of people aged 45 to 85 years when recruited and living within 25-50km of a data collection site (30). The CLSA comprises two cohorts - the Comprehensive cohort (n=30,097), and the Tracking cohort (n=21,241), described in detail elsewhere (Raina et al, 2019). The study protocol was approved by the research ethics board at each research site with the coordination of the McMaster Research Ethics Board. The present study was approved by the University of British Columbia Behavioural Research Ethics Board (UBC BREB, approval number H23-02848). We used data from each of the three data collection waves of the Comprehensive Cohort: baseline (2012-2015), first follow-up (2015-2018), and second follow-up (2018-2021). We included participants aged 50 years and above to be consistent with ELSA. There were 22,605 participants aged 50 to 85 years with data on wealth and primary covariates (age, sex, and race) at baseline. All participants had information on at least one functional outcome (maximum analytical sample for any single outcome = 22,604; minimum analytical sample = 14,402; see S1 Table for N of each analytical sample). For each analytical sample, 82-96% had follow-up outcome data (on one or more time points; see S1 Table). Among those without any follow-up data (N=2695; 10% of the total maximum sample), 1076 had died and the remaining 1619 were lost to follow-up for other reasons. Of the maximum analytic sample, 634 participants (3%) were missing information on one or more of the secondary covariates at baseline (see S3 Table for descriptives of all covariates and functional outcomes at each time point). |
| Participants | 6 | (*a*) *Cohort study*—Give the eligibility criteria, and the sources and methods of selection of participants. Describe methods of follow-up  *Case-control study*—Give the eligibility criteria, and the sources and methods of case ascertainment and control selection. Give the rationale for the choice of cases and controls  *Cross-sectional study*—Give the eligibility criteria, and the sources and methods of selection of participants | pp. 7-8 (Participants, paragraphs 1 and 2)  pp. 8-10 (Measures, paragraphs 1 to 6) | Eligibility criteria are presented within the Participants section: There were 8511 participants aged 50 to 85 years with data on wealth and primary covariates (age, sex, and race) at baseline (wave 6). All participants had information on at least one functional outcome (maximum analytical sample for any single outcome = 8510; minimum analytical sample = 5525; see S1 Table for N of each analytical sample).  We included participants aged 50 years and above to be consistent with ELSA. There were 22,605 participants aged 50 to 85 years with data on wealth and primary covariates (age, sex, and race) at baseline. All participants had information on at least one functional outcome (maximum analytical sample for any single outcome = 22,604; minimum analytical sample = 14,402; see S1 Table for N of each analytical sample).  For both cohorts, the methods of follow up for each measure are described within the Measures section. The methods of data collection for the functional outcomes are described as: Objective measures such as physical performance included gait speed, grip strength, chair stands, and lung function were collected by nurses either during home visits (ELSA) or at designated data collection sites (CLSA). Subjective measures such as sensory function, activities and instrumental activities of daily living and mobility, were obtained through computer-assisted personal interviews (CAPI) conducted by trained interviewers in participants’ homes in both cohorts. |
|  |  | (*b*) *Cohort study*—For matched studies, give matching criteria and number of exposed and unexposed  *Case-control study*—For matched studies, give matching criteria and the number of controls per case |  |  |
| Variables | 7 | Clearly define all outcomes, exposures, predictors, potential confounders, and effect modifiers. Give diagnostic criteria, if applicable | pp. 8-10 (Measures, paragraphs 1 to 6) | See Measures section. |
| Data sources/ measurement | 8* | For each variable of interest, give sources of data and details of methods of assessment (measurement). Describe comparability of assessment methods if there is more than one group | pp. 7-8 (Participants, paragraphs 1 and 2)  pp. 8-10 (Measures, paragraphs 1 to 6) | Details of data sources (ELSA, CLSA) are described in full in the Participants section.  In the Measures section, the methods of collection are described for each cohort. Comparability of assessment between cohorts was considered for each measure, and an overview is presented in S4 Table. |
| Bias | 9 | Describe any efforts to address potential sources of bias | p. 11 (Statistical analysis, paragraph 2)  p. 12 (Statistical analysis, paragraph 1) | Mixed-effects models do not require an equal number of observations from all participants and enable use of data for those with at least one valid outcome measure. All respondents contribute to the intercept term, while those with at least two valid outcome measures contribute to the slope term. Participants therefore contribute data only while they are observed, and once they are no longer present in the study because of death or drop out, they no longer contribute further observations. To account for potential selection bias due to non-random attrition, logistic regression was used to estimate the probability of remaining under observation at follow-up ((having follow-up data in the last 2 waves in ELSA, and in the last wave in CLSA) as a function of age, sex, race, education level, and chronic conditions). Participants who died or were otherwise lost to follow-up were considered not observed at follow-up. Inverse probability weights were defined as the inverse of this predicted probability and were applied in all models.  Missing data on covariates were imputed through multivariate imputation by chained equations (20 imputed data sets) (39). |
| Study size | 10 | Explain how the study size was arrived at | pp. 7-8 (Participants, paragraphs 1 and 2) | Details of the study samples are described in full in the Participants section: There were 8511 participants aged 50 to 85 years with data on wealth and primary covariates (age, sex, and race) at baseline (wave 6). All participants had information on at least one functional outcome (maximum analytical sample for any single outcome = 8510; minimum analytical sample = 5525; see S1 Table for N of each analytical sample). For each analytical sample, 70-80% had follow-up outcome data (on one or more time points; see S1 Table). In ELSA, mortality data is not available for onward sharing after wave 6, limiting direct assessment of mortality-related attrition in this cohort. Of the maximum analytic sample, 2167 participants (25%) were missing information on one or more of the secondary covariates at baseline (primarily due to missing BMI data; see S2 Table for descriptives of all covariates and functional outcomes at each time point).  There were 22,605 participants aged 50 to 85 years with data on wealth and primary covariates (age, sex, and race) at baseline. All participants had information on at least one functional outcome (maximum analytical sample for any single outcome = 22,604; minimum analytical sample = 14,402; see S1 Table for N of each analytical sample). For each analytical sample, 82-96% had follow-up outcome data (on one or more time points; see S1 Table). Among those without any follow-up data (N=2695; 10% of the total maximum sample), 1076 had died and the remaining 1619 were lost to follow-up for other reasons. Of the maximum analytic sample, 634 participants (3%) were missing information on one or more of the secondary covariates at baseline (see S3 Table for descriptives of all covariates and functional outcomes at each time point). |

Continued on next page

| Quantitative variables | 11 | Explain how quantitative variables were handled in the analyses. If applicable, describe which groupings were chosen and why | pp. 8-10 (Measures, paragraphs 1 to 6) | As described in the Measures section, wealth was treated as a categorical variable in both cohorts for comparability. All functional outcomes were analysed as continuous measures; outcomes with skewed distributions were square-root transformed (e.g., ADL, IADL). Categorical covariates included sex, race, number of chronic conditions, weight status, smoking, alcohol consumption, social isolation, loneliness and marital status. Height was analysed as a continuous measure. |
| --- | --- | --- | --- | --- |
| Statistical methods | 12 | (*a*) Describe all statistical methods, including those used to control for confounding | p. 12 (Statistical analysis, paragraph 4) | All statistical methods are described in the Statistical Analysis section. Covariate adjustment is described as: To examine whether the fixed (average) slope varied by wealth status, each model included age (in years, centred at 65 years) and wealth, as well as an interaction term between wealth and age (wealth × age). A significant interaction effect indicates that the average rate of change in functioning varies by wealth status. Covariates in the first set of models were birth cohort (year of birth), sex, race, and height (the latter for gait speed and grip strength analyses only). A second set of models further adjusted for chronic conditions, weight status, smoking, and alcohol consumption, and a third set further adjusted for marital status, social isolation, and loneliness. Missing data on covariates were imputed through multivariate imputation by chained equations (20 imputed data sets) (39). |
|  |  | (*b*) Describe any methods used to examine subgroups and interactions | p. 12 (Statistical analysis, paragraph 5) | See Statistical Analysis section for full description. Interaction assessment is described as: To test whether associations between wealth and functioning varied by sex or race, we re-ran the mixed-effects models additionally including the interaction terms wealth × sex, wealth × race, wealth × sex × age, and wealth × race × age. |
|  |  | (*c*) Explain how missing data were addressed | p. 11 (Statistical analysis, paragraph 2)  p. 12 (Statistical analysis, paragraph 4) | Treatment of missing values is described in the Statistical Analysis section: Mixed-effects models do not require an equal number of observations from all participants and enable use of data for those with at least one valid outcome measure. All respondents contribute to the intercept term, while those with at least two valid outcome measures contribute to the slope term. Participants therefore contribute data only while they are observed, and once they are no longer present in the study because of death or drop out, they no longer contribute further observations. To account for potential selection bias due to non-random attrition, logistic regression was used to estimate the probability of remaining under observation at follow-up ((having follow-up data in the last 2 waves in ELSA, and in the last wave in CLSA) as a function of age, sex, race, education level, and chronic conditions). Participants who died or were otherwise lost to follow-up were considered not observed at follow-up. Inverse probability weights were defined as the inverse of this predicted probability and were applied in all models.  Missing data on covariates were imputed through multivariate imputation by chained equations (20 imputed data sets) (39). |
|  |  | (*d*) *Cohort study*—If applicable, explain how loss to follow-up was addressed  *Case-control study*—If applicable, explain how matching of cases and controls was addressed  *Cross-sectional study*—If applicable, describe analytical methods taking account of sampling strategy | pp. 11-12 (Statistical analysis, paragraphs 2 and 4) | Inverse probability weighting and multiple imputation to handling non-response are described in the Statistical Analysis section. |
|  |  | (*e*) Describe any sensitivity analyses | pp. 12-13 (Statistical analysis, paragraph 6) | Described within the Statistical Analysis section as: In supplementary analyses, we performed a pooled analysis combining data from ELSA and CLSA to further test whether there were cohort differences in the effect of wealth on the level of and mean change in functioning. We included a dummy variable for country, and an interaction between this variable and all other explanatory variables. Second, we repeated the models (within each cohort) using education level as the indicator of socioeconomic disadvantage. As different socioeconomic factors represent distinct constructs that may have different roles in development and ageing (40–42), wealth and education level were examined in separate models. Household wealth and education were moderately correlated (r=0.20 (CLSA) and 0.32 (ELSA), p<0.001), indicating that the constructs are not redundant measures of socioeconomic position. In an additional supplementary analysis conducted in response to peer review, we examined disease categories as covariates (respiratory, muscoskeletal, neurological, mental, cardiovascular, diabetes, cancer; see S4 Table for conditions included in each category), rather than the total number of chronic conditions to assess the robustness of the primary findings. |
| Results | | | | |
| Participants | 13* | (a) Report numbers of individuals at each stage of study—eg numbers potentially eligible, examined for eligibility, confirmed eligible, included in the study, completing follow-up, and analysed | pp. 7-8 (Participants, paragraphs 1 and 2) | This information is included in the Participants section: There were 8511 participants aged 50 to 85 years with data on wealth and primary covariates (age, sex, and race) at baseline (wave 6). All participants had information on at least one functional outcome (maximum analytical sample for any single outcome = 8510; minimum analytical sample = 5525; see S1 Table for N of each analytical sample). For each analytical sample, 70-80% had follow-up outcome data (on one or more time points; see S1 Table). In ELSA, mortality data is not available for onward sharing after wave 6, limiting direct assessment of mortality-related attrition in this cohort. Of the maximum analytic sample, 2167 participants (25%) were missing information on one or more of the secondary covariates at baseline (primarily due to missing BMI data; see S2 Table for descriptives of all covariates and functional outcomes at each time point).  There were 22,605 participants aged 50 to 85 years with data on wealth and primary covariates (age, sex, and race) at baseline. All participants had information on at least one functional outcome (maximum analytical sample for any single outcome = 22,604; minimum analytical sample = 14,402; see S1 Table for N of each analytical sample). For each analytical sample, 82-96% had follow-up outcome data (on one or more time points; see S1 Table). Among those without any follow-up data (N=2695; 10% of the total maximum sample), 1076 had died and the remaining 1619 were lost to follow-up for other reasons. Of the maximum analytic sample, 634 participants (3%) were missing information on one or more of the secondary covariates at baseline (see S3 Table for descriptives of all covariates and functional outcomes at each time point). |
|  |  | (b) Give reasons for non-participation at each stage | pp. 7-8 (Participants, paragraphs 1 and 2) | In ELSA, mortality data is not available for onward sharing after wave 6, limiting direct assessment of mortality-related attrition in this cohort.  Among those without any follow-up data (N=2695; 10% of the total maximum sample), 1076 had died and the remaining 1619 were lost to follow-up for other reasons. |
|  |  | (c) Consider use of a flow diagram |  |  |
| Descriptive data | 14* | (a) Give characteristics of study participants (eg demographic, clinical, social) and information on exposures and potential confounders | p. 13 (Results, paragraph 1) | Table 1 describes baseline characteristics of both cohorts. Referenced in Results. |
|  |  | (b) Indicate number of participants with missing data for each variable of interest | pp. 7-8 (Participants, paragraphs 1 and 2) | There were 8511 participants aged 50 to 85 years with data on wealth and primary covariates (age, sex, and race) at baseline (wave 6). All participants had information on at least one functional outcome (maximum analytical sample for any single outcome = 8510; minimum analytical sample = 5525; see S1 Table for N of each analytical sample). For each analytical sample, 70-80% had follow-up outcome data (on one or more time points; see S1 Table). Of the maximum analytic sample, 2167 participants (25%) were missing information on one or more of the secondary covariates at baseline (primarily due to missing BMI data; see S2 Table for descriptives of all covariates and functional outcomes at each time point).  There were 22,605 participants aged 50 to 85 years with data on wealth and primary covariates (age, sex, and race) at baseline. All participants had information on at least one functional outcome (maximum analytical sample for any single outcome = 22,604; minimum analytical sample = 14,402; see S1 Table for N of each analytical sample). For each analytical sample, 82-96% had follow-up outcome data (on one or more time points; see S1 Table). Among those without any follow-up data (N=2695; 10% of the total maximum sample), 1076 had died and the remaining 1619 were lost to follow-up for other reasons. Of the maximum analytic sample, 634 participants (3%) were missing information on one or more of the secondary covariates at baseline (see S3 Table for descriptives of all covariates and functional outcomes at each time point). |
|  |  | (c) *Cohort study*—Summarise follow-up time (eg, average and total amount) | p. 7 (Participants, paragraph 1) | We used data from waves 6 (2012-2013), 8 (2016-2017), 9 (2018-2019), and 10 (2021-2023; 11 years of follow-up) to allow for a closer comparison with CLSA in terms of timing (2012-2021; 9 years of follow-up). |
| Outcome data | 15* | *Cohort study*—Report numbers of outcome events or summary measures over time | pp. 7-8 (Participants, paragraphs 1 and 2) | The analytic samples for both cohorts are listed in the Participants section and S1 Table. |
|  |  | *Case-control study—*Report numbers in each exposure category, or summary measures of exposure |  |  |
|  |  | *Cross-sectional study—*Report numbers of outcome events or summary measures |  |  |
| Main results | 16 | (*a*) Give unadjusted estimates and, if applicable, confounder-adjusted estimates and their precision (eg, 95% confidence interval). Make clear which confounders were adjusted for and why they were included | pp. 13-14 (Results, paragraphs 2 and 3) | Model estimates are presented in S5 Table and S6 Table and referenced in the Results section. |
|  |  | (*b*) Report category boundaries when continuous variables were categorized |  |  |
|  |  | (*c*) If relevant, consider translating estimates of relative risk into absolute risk for a meaningful time period |  |  |

Continued on next page

| Other analyses | 17 | Report other analyses done—eg analyses of subgroups and interactions, and sensitivity analyses | pp. 14-15 (Results, paragraphs 2 to 5) | Results of additional analyses are presented in S7-10 Tables. Referenced within Results. |
| --- | --- | --- | --- | --- |
| Discussion | | | | |
| Key results | 18 | Summarise key results with reference to study objectives | p. 15 (Discussion, paragraph 1) | In this longitudinal cross-country comparison between England and Canada, we observed socioeconomic inequalities in the level of functioning in both countries across multiple outcomes. Socioeconomic inequalities in the level of functioning were generally larger in England than in Canada, especially for gait speed, ADL, and IADL. Moreover, we observed socioeconomic inequalities in functional ageing trajectories in both countries, such that less wealth was associated with greater decline in mobility and in the ability to perform ADL and IADL with age. Wealth inequalities in functional health and ageing were attenuated but persisted after adjustment for common risk factors including health behaviours, weight status, chronic conditions, and social connections. There was some evidence that associations between wealth and functioning were modified by sex, such that women with the least wealth experienced greater difficulties with ADL, IADL, poorer mobility (timed up and go performance), and worse hearing than men with the least wealth, but better lung function. |
| Limitations | 19 | Discuss limitations of the study, taking into account sources of potential bias or imprecision. Discuss both direction and magnitude of any potential bias | pp. 19-20 (Discussion, paragraph 7) | Gait speed was measured with a standard walking test in each cohort and expressed in standardized units (metres per second). However, we acknowledge that, while the tests in each cohort used a static start, they differed in terms of the number of tests (two 8-foot tests in ELSA, one 4-metre test in CLSA). These methodological differences may influence the comparability of the results between cohorts. While we chose an outcome-wide approach, further insights into ageing trajectories across cohorts will be gained using validated aggregate longitudinal measures of ageing, such as the recently updated Pace of Ageing measure. We did not consider all potential interactions, such as interactions between sex and race in associations between socioeconomic conditions and functional ageing trajectories, which could be the focus of future studies. An additional limitation is the underrepresentation of non-white participants in both cohorts, which limited our ability to examine racial and ethnic heterogeneity, and may reduce the generalisability of our findings to increasingly diverse older populations. Our analysis compared two liberal welfare states, which has the advantage of isolating subtler sources of variation that would be harder to detect in broader cross-regime comparisons. Comparing cohorts in other countries with different welfare models will provide insights into the role of broader institutional contexts in diverging ageing trajectories. However, such comparisons remain challenging, due to the limited availability of harmonised longitudinal datasets with sufficient follow-up and comparable socioeconomic measures across these settings. Although we examined the contribution of important factors related to the ageing process, we could not examine all potential factors contributing to the observed associations. Access to general practitioner (GP) services, dentists, other health and personal care, as well as time on waiting lists (and move to private healthcare) have been introduced in ELSA in the latest wave (11) and should be examined in future research. Future research should also examine the contribution of specific occupational and environmental exposures (which likely vary by functional outcome) to provide additional insights beyond the contributing factors examined in this study. Although wealth is the most precise measure of socioeconomic resources in older adulthood, we did not have the exact same measures of wealth in each cohort for more accurate comparison. However, when using education as the socioeconomic indicator (which was measured in the same way in each cohort), the direction of the associations was similar to that for wealth, and we could still see that associations were generally larger in ELSA than in CLSA. We are therefore assured that our conclusions are not driven by differences in measurement between the two cohorts. Although we used inverse probability weighting to account for non-random attrition (mortality and drop-out), socioeconomic gradients in functional decline may be conservatively estimated: socioeconomically disadvantaged individuals have higher mortality and exit the analytic sample earlier, which may attenuate observed inequalities among those remaining under observation. We focused on measures of socioeconomic conditions in adulthood, but a life course approach would provide further insights, as childhood socioeconomic conditions have been associated with functional ageing trajectories beyond socioeconomic conditions in adulthood. |
| Interpretation | 20 | Give a cautious overall interpretation of results considering objectives, limitations, multiplicity of analyses, results from similar studies, and other relevant evidence | pp. 20-21 (Discussion, paragraphs 8 and 9) | Interpretations of results are described within the policy implications and concluding paragraph of the Discussion section. |
| Generalisability | 21 | Discuss the generalisability (external validity) of the study results | pp. 19-20 (Discussion, paragraphs 6 and 7) | The generalisability of results is discussed on page 19 and within the Strengths and limitations paragraph. |
| Other information | |  | | |
| Funding | 22 | Give the source of funding and the role of the funders for the present study and, if applicable, for the original study on which the present article is based | pp. 21-22 (Acknowledgements and Funding) | Funding sources of the present study and the original study are provided in the Acknowledgements and Funding sections. |

*Give information separately for cases and controls in case-control studies and, if applicable, for exposed and unexposed groups in cohort and cross-sectional studies.

**Note:** An Explanation and Elaboration article discusses each checklist item and gives methodological background and published examples of transparent reporting. The STROBE checklist is best used in conjunction with this article (freely available on the Web sites of PLoS Medicine at http://www.plosmedicine.org/, Annals of Internal Medicine at http://www.annals.org/, and Epidemiology at http://www.epidem.com/). Information on the STROBE Initiative is available at www.strobe-statement.org.
