# Supplementary material for: Socioeconomic inequalities in functional ageing trajectories in England and Canada: A comparative longitudinal cohort study
Source: PLoS Med. 2026 Aug 13;23(8):e1004833. doi: 10.1371/journal.pmed.1004833 (PMC13472442; doi:10.1371/journal.pmed.1004833)
Supplement: Supplementary file 2 [file pmed.1004833.s002.docx]

**S1 Table. Size of each analytical sample in the two cohorts**

| **ELSA** | **Baseline N^1^** | **Follow-up N^2^** |
| --- | --- | --- |
| Gait speed^3^ | 5525 | 4311 |
| Grip strength | 7164 | 5215 |
| Chair rise^4^ | 6146 | 4274 |
| FEV^4^ | 6733 | 4718 |
| Eyesight | 8507 | 6777 |
| Hearing | 8508 | 6778 |
| ADL difficulties | 8509 | 6780 |
| IADL difficulties | 8509 | 6780 |
| Mobility difficulties | 8510 | 6780 |
|  |  |  |
| **CLSA** |  |  |
| Gait speed^3^ | 14,402 | 12,651 |
| Grip strength | 21,044 | 18,785 |
| Chair rise | 21,853 | 19,158 |
| FEV | 17,413 | 14,261 |
| Eyesight | 22,596 | 21,307 |
| Hearing | 22,584 | 21,299 |
| ADL difficulties | 22,604 | 21,667 |
| IADL difficulties | 22,604 | 21,667 |
| Up and go time | 22,479 | 20,033 |

ELSA=English Longitudinal Study of Ageing; CLSA=Canadian Longitudinal Study on Aging; FEV=Forced Expiratory Volume; ADL=basic activities of daily living; IADL=instrumental activities of daily living.

^1^Participants with information on wealth, primary covariates (age, sex, and race), and at least one functional outcome at baseline, and therefore contributed to the model intercept term

^2^Participants with information on wealth, primary covariates (age, sex, and race), and functional outcomes on at least one additional time point, and therefore contributed to the model slope term

^3^Participants were aged 50 to 85 years for all outcomes apart from gait speed, which included participants aged 60 to 85 years

^4^Baseline was wave 2 for these outcomes
