# Supplementary material for: Socioeconomic inequalities in functional ageing trajectories in England and Canada: A comparative longitudinal cohort study
Source: PLoS Med. 2026 Aug 13;23(8):e1004833. doi: 10.1371/journal.pmed.1004833 (PMC13472442; doi:10.1371/journal.pmed.1004833)
Supplement: Supplementary file 3 [file pmed.1004833.s003.docx]

**S2 Table. Descriptives of model covariates and functional outcomes at each time point in ELSA**

|  | **Wave 6 (2012/13)** | **Wave 8 (2016/17)** | **Wave 9 (2018/19)** | **Wave 10 (2021/23)** |
| --- | --- | --- | --- | --- |
|  | N=8,511 | N=6,552 | N=5,855 | N=4,000 |
| Age (years) | 66.7 (8.6) | 70.3 (8.2) | 71.9 (8.0) | 73.6 (7.3) |
| Sex |  |  |  |  |
| Male | 44.8 (3,816) | 44.4 (2,912) | 43.9 (2,568) | 44.8 (1,791) |
| Female | 55.2 (4,695) | 55.6 (3,640) | 56.1 (3,287) | 55.2 (2,209) |
| Race |  |  |  |  |
| White | 96.4 (8,202) | 96.7 (6,333) | 96.7 (5,661) | 97.2 (3,889) |
| Other | 3.6 (309) | 3.3 (219) | 3.3 (194) | 2.8 (111) |
| Marital/partner status |  |  |  |  |
| Married/living with a partner | 65.1 (5,542) | 63.1 (4,134) | 61.8 (3,616) | 62.2 (2,489) |
| Other | 34.9 (2,969) | 36.9 (2,418) | 38.2 (2,239) | 37.8 (1,511) |
| Chronic health conditions |  |  |  |  |
| None | 37.4 (3,185) | 29.1 (1,906) | 30.5 (1,786) | 14.2 (568) |
| One | 30.5 (2,598) | 32.7 (2,143) | 32.0 (1,875) | 27.6 (1,102) |
| Two | 21.0 (1,790) | 24.3 (1,595) | 24.0 (1,404) | 29.4 (1,175) |
| Three or more | 11.0 (938) | 13.9 (908) | 13.5 (790) | 28.9 (1,155) |
| Weight status |  |  |  |  |
| Healthy weight | 25.5 (1,790) | 27.7 (1,525) | 29.2 (1,411) | 30.4 (708) |
| Underweight | 0.9 (60) | 1.1 (61) | 1.3 (61) | 2.1 (48) |
| Overweight | 41.4 (2,908) | 40.2 (2,219) | 41.2 (1,988) | 40.6 (945) |
| Obesity | 32.3 (2,266) | 31.0 (1,710) | 28.3 (1,368) | 27.0 (629) |
| Smoking status |  |  |  |  |
| Never smoked | 37.6 (3,188) | 37.0 (2,411) | 37.2 (2,176) | 38.0 (1,518) |
| Former smoker | 49.7 (4,212) | 54.0 (3,516) | 54.5 (3,185) | 56.1 (2,238) |
| Current smoker | 12.6 (1,069) | 9.0 (589) | 8.3 (483) | 5.9 (235) |
| Frequency of alcohol consumption |  |  |  |  |
| Not in the past 12 months | 13.2 (973) | 14.4 (822) | 15.1 (786) | 15.7 (554) |
| Less than once a month | 16.4 (1,208) | 17.2 (986) | 16.9 (878) | 16.5 (581) |
| Once or twice a month | 11.6 (857) | 12.3 (703) | 12.0 (626) | 10.2 (359) |
| At least once a week | 37.9 (2,793) | 35.9 (2,058) | 36.4 (1,893) | 37.0 (1,305) |
| Almost every day | 20.8 (1,536) | 20.2 (1,157) | 19.5 (1,013) | 20.7 (730) |
| Lonely |  |  |  |  |
| No | 79.0 (5,811) | 81.7 (4,628) | 81.4 (4,159) | 74.4 (2,602) |
| Yes | 21.0 (1,544) | 18.3 (1,037) | 18.6 (948) | 25.6 (897) |
| Socially isolated |  |  |  |  |
| No | 40.6 (3,045) | 41.2 (2,386) | 43.1 (2,259) | 43.7 (1,568) |
| Yes | 59.4 (4,450) | 58.8 (3,411) | 56.9 (2,986) | 56.3 (2,020) |
| Gait speed, metres/second | 0.9 (0.3) | 0.9 (0.3) | 0.9 (0.3) | 0.8 (0.3) |
| Max grip strength, kilograms | 30.9 (11.1) | 30.3 (11.0) | 29.0 (10.2) | - |
| Eyesight rating, 1=excellent, 5=poor | 2.5 (1.0) | 2.5 (1.0) | 2.6 (1.0) | 2.6 (1.0) |
| Hearing rating, 1=excellent, 5=poor | 2.6 (1.1) | 2.7 (1.1) | 2.8 (1.1) | 2.8 (1.1) |
| Number of ADL difficulties, square root | 0.2 (0.5) | 0.2 (0.6) | 0.3 (0.6) | 0.3 (0.6) |
| Number of IADL difficulties, square root | 0.2 (0.6) | 0.3 (0.6) | 0.3 (0.6) | 0.3 (0.6) |
| Number of mobility difficulties | 1.9 (2.6) | 2.0 (2.6) | 2.0 (2.6) | 2.1 (2.6) |
|  | **Wave 2 (2004/05)** | **Wave 4 (2008/09)** | **Wave 6 (2012/13)** |  |
|  | N=7049 | N=5395 | N=4689 |  |
| Total time for 5 chair rises, seconds | 2.4 (0.3) | 2.4 (0.3) | 2.4 (0.3) |  |
| Forced expiratory volume, litres | 2.3 (0.8) | 2.3 (0.8) | 2.2 (0.8) |  |

ELSA=English Longitudinal Study of Ageing; CLSA=Canadian Longitudinal Study on Aging; ADL=basic activities of daily living; IADL=instrumental activities of daily living.
