# Supplementary material for: Socioeconomic inequalities in functional ageing trajectories in England and Canada: A comparative longitudinal cohort study
Source: PLoS Med. 2026 Aug 13;23(8):e1004833. doi: 10.1371/journal.pmed.1004833 (PMC13472442; doi:10.1371/journal.pmed.1004833)
Supplement: Supplementary file 4 [file pmed.1004833.s004.docx]

**S3 Table. Descriptives of model covariates and functional outcomes at each time point in CLSA**

|  | **Baseline (2012/15)** | **Follow-up 1 (2015/18)** | **Follow-up 2 (2018/21)** |
| --- | --- | --- | --- |
|  | N=22,605 | N=21,607 | N=19,910 |
| Age (years) | 64.1 (9.2) | 67.0 (9.1) | 69.5 (8.9) |
| Sex |  |  |  |
| Male | 51.2 (11,578) | 51.1 (11,041) | 50.7 (10,095) |
| Female | 48.8 (11,027) | 48.9 (10,566) | 49.3 (9,815) |
| Race |  |  |  |
| White | 97.0 (21,921) | 97.1 (20,969) | 97.2 (19,344) |
| Other | 3.0 (684) | 2.9 (638) | 2.8 (566) |
| Marital/partner status |  |  |  |
| Married/living with a partner | 69.0 (15,603) | 67.9 (14,663) | 67.4 (13,366) |
| Other | 31.0 (6,996) | 32.1 (6,936) | 32.6 (6,474) |
| Chronic health conditions |  |  |  |
| None | 22.3 (5,043) | 18.1 (3,793) | 15.6 (3,036) |
| One | 30.9 (6,983) | 29.2 (6,116) | 28.4 (5,545) |
| Two | 25.6 (5,788) | 26.8 (5,606) | 27.5 (5,360) |
| Three or more | 21.1 (4,752) | 25.8 (5,397) | 28.6 (5,573) |
| Weight status |  |  |  |
| Healthy weight | 28.2 (6,367) | 28.6 (5,984) | 31.0 (6,045) |
| Underweight | 0.7 (153) | 0.8 (161) | 0.9 (172) |
| Overweight | 41.2 (9,300) | 40.9 (8,553) | 40.1 (7,804) |
| Obesity | 30.0 (6,769) | 29.8 (6,234) | 28.0 (5,458) |
| Smoking status |  |  |  |
| Never smoked | 46.3 (10,469) | 48.3 (10,262) | 51.6 (10,087) |
| Former smoker | 45.2 (10,225) | 44.6 (9,471) | 42.6 (8,341) |
| Current smoker | 8.4 (1,910) | 7.1 (1,503) | 5.8 (1,131) |
| Frequency of alcohol consumption |  |  |  |
| Not in the past 12 months | 11.3 (2,496) | 10.0 (2,117) | 14.9 (2,971) |
| Less than once a month | 11.7 (2,593) | 12.0 (2,531) | 11.1 (2,213) |
| Once or twice a month | 16.3 (3,610) | 16.6 (3,497) | 15.8 (3,143) |
| At least once a week | 42.8 (9,474) | 42.9 (9,047) | 40.3 (8,017) |
| Almost every day | 17.9 (3,952) | 18.4 (3,876) | 17.8 (3,531) |
| Lonely |  |  |  |
| No | 74.9 (16,867) | 76.6 (15,964) | 76.3 (14,818) |
| Yes | 25.1 (5,663) | 23.4 (4,867) | 23.7 (4,595) |
| Socially isolated |  |  |  |
| No | 74.1 (16,697) | 81.8 (14,655) | 65.2 (12,521) |
| Yes | 25.9 (5,839) | 18.2 (3,266) | 34.8 (6,688) |
| Gait speed, metres/second | 1.0 (0.2) | 1.0 (0.2) | 0.9 (0.2) |
| Max grip strength, kilograms | 35.3 (11.6) | 34.3 (11.6) | 33.3 (11.2) |
| Average time for 1 chair rise, log seconds | 1.0 (0.3) | 0.9 (0.3) | 0.9 (0.3) |
| Forced expiratory volume, litres | 2.7 (0.8) | 2.6 (0.7) | 2.6 (0.7) |
| Eyesight rating, 1=excellent, 5=poor | 2.2 (0.9) | 2.4 (0.9) | 2.3 (0.9) |
| Hearing rating, 1=excellent, 5=poor | 2.4 (1.0) | 2.6 (0.9) | 2.5 (0.9) |
| Number of ADL difficulties, square root | 0.1 (0.4) | 0.2 (0.4) | 0.2 (0.4) |
| Number of IADL difficulties, square root | 0.1 (0.3) | 0.1 (0.3) | 0.1 (0.4) |
| Up and go time, log seconds | 2.2 (0.2) | 2.3 (0.2) | 2.3 (0.2) |

ELSA=English Longitudinal Study of Ageing; CLSA=Canadian Longitudinal Study on Aging; ADL=basic activities of daily living; IADL=instrumental activities of daily living.
