# Supplementary material for: Socioeconomic inequalities in functional ageing trajectories in England and Canada: A comparative longitudinal cohort study
Source: PLoS Med. 2026 Aug 13;23(8):e1004833. doi: 10.1371/journal.pmed.1004833 (PMC13472442; doi:10.1371/journal.pmed.1004833)
Supplement: Supplementary file 5 [file pmed.1004833.s005.docx]

**S4 Table. Comparison of the study measures in the two cohorts**

| **Construct** | **ELSA measure** | **CLSA measure** | **Comparability/harmonisation** |
| --- | --- | --- | --- |
| **Socioeconomic factors** |  |  |  |
| Household wealth | Total non-pension household wealth measured as net total wealth of a benefit unit (a single adult or a married or cohabiting couple and any dependent children), comprising the sum of savings and investments after financial debt has been subtracted | Total value of savings and investments (of a single adult or a couple), categorised as less than $50,000, $50,000 to less than $100,000, $100,000 to less than $1 million, and $1 million or more | To improve comparability, wealth in ELSA was categorised using cohort-specific quantiles to match the distribution of the CLSA wealth categories, thereby capturing relative socioeconomic position within each cohort |
|  |  |  |  |
| Education level | Highest education level categorised as: less than o-level or equivalent, o-level or equivalent, higher than a-level | Highest education level categorised as: less than secondary school graduation, secondary school graduation (no post-secondary education), some post-secondary education, post-secondary degree/diploma | Harmonised categories as follows: less than secondary school, secondary school, or post-secondary education |
|  |  |  |  |
| **Functional outcomes** |  |  |  |
| Gait speed | Two timed 8-foot walking test from a standing start | One timed 4-metre walking test from a standing start | Gait speed was m/s, with ELSA test performance taken as the average of the two tests |
|  |  |  |  |
| Grip strength | Assessed across three trials using a hand dynamometer | Assessed across three trials using a hand dynamometer | Grip strength was maximum value in kg across the three trials using the dominant hand |
|  |  |  |  |
| Chair rise performance | Time to complete 5 chair rises | Time to complete 5 chair rises | Average time in seconds for one chair rise was time to complete 5 rises divided by 5 |
|  |  |  |  |
| Lung function | Forced expiratory volume (FEV, litres), assessed by spirometry | Forced expiratory volume (FEV, litres), assessed by spirometry | Highest technically satisfactory reading across three successful measures |
|  |  |  |  |
| Eyesight | Self-rated eyesight, ranging from 1=excellent to 5=poor | Self-rated eyesight, ranging from 1=excellent to 5=poor | Identical measures |
|  |  |  |  |
| Hearing | Self-rated hearing, ranging from 1=excellent to 5=poor | Self-rated hearing, ranging from 1=excellent to 5=poor | Identical measures |
|  |  |  |  |
| Basic activities of daily living (ADL) | Katz ADL scale, self-report  6 ADL tasks, scored as independent (0) and dependent (1) | OARS ADL scale, self-report  7 ADL tasks, scored as 0 (able) and 1 (unable) | Sum of self-reported limitations on the same 6 tasks |
|  |  |  |  |
|  |  |  |  |
| Instrumental activities of daily living (IADL) | Lawton IADL scale, self-report  7 IADL tasks, scored as independent (0) and dependent (1) | OARS IADL scale, self-report  7 IADL tasks, scored as 0 (able) and 1 (unable) | Sum of self-reported limitations on the same 7 tasks |
|  |  |  |  |
| Mobility | Sum of difficulties doing the following: walking 100 yards, sitting 2 hours, getting up from chair, climbing stairs, stooping, kneeling or crouching, reaching arms, pulling or pushing large objects, lifting weight over 20 pounds, picking up coin from table | NA | NA |
|  |  |  |  |
| Up and go test | NA | Timed test performance whereby the participant starts in a seated position, stands up upon the signal, walks a distance of 3 metres at a normal pace, turns around, walks back, and sits down | NA |
| **Covariates** |  |  |  |
| Smoking | Self-reported smoking status (current smoker, former smoker, non-smoker) | Self-reported smoking status (current smoker, former smoker, non-smoker) | Identical measures |
|  |  |  |  |
| Alcohol consumption | Self-reported frequency of alcohol consumption, categorised as: almost every day, at least once a week, once or twice a month, less than once a month, not in the past 12 months | Self-reported frequency of alcohol consumption, categorised as: almost every day (incl. 6 times a week), 4-5 times a week, 2-3 times a week, once a week, 2-3 times a month, about once a month, less than once a month, never | Harmonised categories as follows: almost every day (almost every day (incl. 6 times a week)), at least once a week (4-5 times a week, 2-3 times a week, once a week), once or twice a month (2-3 times a month, about once a month), less than once a month (less than once a month), not in the past 12 months (never) |
|  |  |  |  |
| Chronic health conditions | Self-reported physician diagnosis of the following conditions: chronic lung disease such as chronic bronchitis or emphysema, asthma, arthritis (including osteoarthritis or rheumatism), osteoporosis, cancer or a malignant tumour (excluding minor skin cancers), parkinson's disease, any emotional, nervous or psychiatric problems, alzheimer's disease, dementia, senility or another serious memory impairment, high blood pressure or hypertension, angina, a heart attack (including myocardial infarction or coronary thrombosis), congestive heart failure, diabetes or high blood sugar, a stroke (cerebral vascular disease), valvular heart disease, and ischaemic heart disease | Self-reported physician diagnosis of the following conditions: chronic lung disease such as chronic bronchitis or emphysema, asthma, arthritis (including osteoarthritis or rheumatism), osteoporosis, cancer or a malignant tumour (excluding minor skin cancers), parkinson's disease, any emotional, nervous or psychiatric problems, alzheimer's disease, dementia, senility or another serious memory impairment, high blood pressure or hypertension, angina, a heart attack (including myocardial infarction or coronary thrombosis), congestive heart failure, diabetes or high blood sugar, a stroke (cerebral vascular disease), valvular heart disease, and ischaemic heart disease | The number of conditions were summed and categorised as none, one, two, or three or more. In supplementary analyses, the chronic health condition categories were respiratory (chronic lung disease or asthma), muscoskeletal (arthritis or osteoporosis), neurological (parkinson's disease, alzheimer's disease, dementia, senility or another serious memory impairment, or stroke), mental (any emotional, nervous or psychiatric problems), cardiovascular (high blood pressure or hypertension, angina, a heart attack, congestive heart failure, valvular heart disease, or ischaemic heart disease), diabetes, and cancer. |
|  |  |  |  |
| Weight status | Height and weight measured by research nurses (waves 2 to 6) and self-reported (waves 8 to 10), and used to calculate BMI (weight (kg) / height (m)^2^) | Height and weight measured by trained research assistants, and used to calculate BMI (weight (kg) / height (m)^2^) | Defined using WHO classification for adults: underweight (<18.5), healthy weight (18.5–24.9), overweight (25.0–29.9), and obesity (≥30) |
|  |  |  |  |
| Marital status | Married, cohabiting, neither | Single (never married or never lived with a partner), married/living with a partner in a common-law relationship, widowed, divorced, separated | Harmonised categories as follows: married or cohabiting (married/living with a partner in a common-law relationship), not married or cohabiting (single (never married or never lived with a partner), widowed, divorced, separated) |
|  |  |  |  |
| Social isolation | How often the respondent meets up with each of children, other family members, and friends on average, and participation in social clubs and groups | Last get together with each of children, other family members, and friends, and participation in social clubs and groups | A social isolation index was created by assigning one point if the respondent had less than monthly contact with each of children, other family members, and friends, and if they did not participate in social clubs or groups. The top quintile (a score ≥2) was defined as being isolated |
|  |  |  |  |
| Loneliness | UCLA-3 loneliness scale (e.g. ‘how often do you feel that you lack companioship’), each item scored on a 3-point scale (hardly ever, some of the time, often) | Item from the CES-D 10 scale (‘in the past week, how often have you feel lonely?’), scored on a 4-point scale (rarely or never, some of the time, occasionally, all of the time) | Responses were categorised so that those who reported feeling lonely some of the time were identified as being lonely (versus not lonely) |

ELSA=English Longitudinal Study of Ageing; CLSA=Canadian Longitudinal Study on Aging; OARS ADL scale = Older Americans Resources and Services Activities of Daily Living Scale; UCLA-3 = short form (3 items) of the University of California, Los Angeles loneliness scale; CESD-10 = 10-item Center for Epidemiologic Studies Depression Scale; WHO=World Health Organisation; NA=not applicable.
