# Supplementary material for: Socioeconomic inequalities in functional ageing trajectories in England and Canada: A comparative longitudinal cohort study
Source: PLoS Med. 2026 Aug 13;23(8):e1004833. doi: 10.1371/journal.pmed.1004833 (PMC13472442; doi:10.1371/journal.pmed.1004833)
Supplement: Supplementary file 6 [file pmed.1004833.s006.docx]

**S5 Table. Associations between wealth and the level of and change in functional health outcomes in ELSA**

|  | **Model 1** | | | **Model 2** | | | **Model 3** | | |
| --- | --- | --- | --- | --- | --- | --- | --- | --- | --- |
|  | b | 95% CI | p | b | 95% CI | p | b | 95% CI | p |
| **Gait speed, m/s^1^** |  |  |  |  |  |  |  |  |  |
| Age | 0.010 | [-0.012,0.032] | 0.358 | 0.010 | [-0.012,0.031] | 0.394 | 0.010 | [-0.012,0.032] | 0.375 |
| Age^2^ | 0.001 | [0.000,0.001] | <0.001 | <0.001 | [0.000,0.001] | <0.001 | 0.000 | [0.000,0.001] | <0.001 |
| Ref: Highest wealth |  |  |  |  |  | <0.001 |  |  | <0.001 |
| Higher wealth | 0.087 | [0.062,0.111] | <0.001 | 0.072 | [0.047,0.096] | <0.001 | 0.070 | [0.045,0.094] | <0.001 |
| Lower wealth | 0.167 | [0.136,0.199] | <0.001 | 0.135 | [0.103,0.166] | <0.001 | 0.130 | [0.098,0.162] | <0.001 |
| Lowest wealth | 0.264 | [0.233,0.295] | <0.001 | 0.216 | [0.185,0.248] | <0.001 | 0.208 | [0.176,0.241] | <0.001 |
| Ref: Highest wealth x Age |  |  |  |  |  |  |  |  |  |
| Higher wealth x Age | -0.001 | [-0.004,0.002] | 0.452 | -0.001 | [-0.003,0.002] | 0.632 | -0.001 | [-0.003,0.002] | 0.671 |
| Lower wealth x Age | -0.002 | [-0.005,0.001] | 0.130 | -0.002 | [-0.005,0.001] | 0.307 | -0.001 | [-0.005,0.002] | 0.350 |
| Lowest wealth x Age | -0.004 | [-0.007,-0.001] | 0.021 | -0.003 | [-0.006,0.000] | 0.080 | -0.003 | [-0.006,0.001] | 0.116 |
| Observations | 14,714 |  |  |  |  |  |  |  |  |
| **Grip strength, kg^1^** |  |  |  |  |  |  |  |  |  |
| Age | -0.143 | [-0.635,0.349] | 0.57 | -0.259 | [-0.751,0.233] | 0.303 | -0.250 | [-0.743,0.243] | 0.321 |
| Age^2^ | 0.008 | [0.007,0.010] | <0.001 | 0.008 | [0.006,0.010] | <0.001 | 0.007 | [0.006,0.009] | <0.001 |
| Ref: Highest wealth |  |  |  |  |  |  |  |  |  |
| Higher wealth | 0.489 | [-0.038,1.015] | 0.069 | 0.529 | [-0.003,1.061] | 0.051 | 0.530 | [-0.004,1.063] | 0.052 |
| Lower wealth | 1.874 | [1.199,2.549] | <0.001 | 1.898 | [1.208,2.588] | <0.001 | 1.906 | [1.210,2.601] | <0.001 |
| Lowest wealth | 2.477 | [1.826,3.127] | <0.001 | 2.456 | [1.773,3.139] | <0.001 | 2.470 | [1.769,3.171] | <0.001 |
| Ref: Highest wealth x Age |  |  |  |  |  |  |  |  |  |
| Higher wealth x Age | 0.053 | [0.000,0.105] | 0.049 | 0.047 | [-0.005,0.100] | 0.079 | 0.047 | [-0.005,0.100] | 0.079 |
| Lower wealth x Age | 0.036 | [-0.028,0.100] | 0.267 | 0.023 | [-0.043,0.088] | 0.497 | 0.021 | [-0.045,0.087] | 0.534 |
| Lowest wealth x Age | 0.008 | [-0.055,0.070] | 0.815 | -0.005 | [-0.071,0.061] | 0.884 | -0.008 | [-0.077,0.060] | 0.809 |
| Observations | 16,106 |  |  |  |  |  |  |  |  |
| **Chair rise time, log s** |  |  |  |  |  |  |  |  |  |
| Age | 0.007 | [0.004,0.010] | <0.001 | 0.010 | [0.005,0.015] | <0.001 | 0.011 | [0.006,0.017] | <0.001 |
| Age^2^ | 0.000 | [0.000,0.000] | <0.001 | 0.000 | [0.000,0.000] | <0.001 | 0.000 | [0.000,0.000] | <0.001 |
| Ref: Highest wealth |  |  |  |  |  |  |  |  |  |
| Higher wealth | 0.057 | [0.034,0.080] | <0.001 | 0.049 | [0.025,0.072] | <0.001 | 0.048 | [0.025,0.071] | <0.001 |
| Lower wealth | 0.134 | [0.105,0.163] | <0.001 | 0.117 | [0.087,0.146] | <0.001 | 0.116 | [0.086,0.146] | <0.001 |
| Lowest wealth | 0.184 | [0.154,0.214] | <0.001 | 0.161 | [0.129,0.192] | <0.001 | 0.159 | [0.127,0.191] | <0.001 |
| Ref: Highest wealth x Age |  |  |  |  |  |  |  |  |  |
| Higher wealth x Age | -0.001 | [-0.004,0.002] | 0.604 | -0.001 | [-0.004,0.002] | 0.544 | -0.001 | [-0.004,0.002] | 0.482 |
| Lower wealth x Age | -0.001 | [-0.005,0.003] | 0.585 | -0.001 | [-0.005,0.003] | 0.582 | -0.001 | [-0.005,0.002] | 0.460 |
| Lowest wealth x Age | -0.004 | [-0.007,0.000] | 0.054 | -0.004 | [-0.008,0.000] | 0.041 | -0.005 | [-0.009,-0.001] | 0.022 |
| Observations | 13,252 |  |  |  |  |  |  |  |  |
| **FEV, litres^1^** |  |  |  |  |  |  |  |  |  |
| Age | 0.033 | [0.027,0.039] | <0.001 | 0.034 | [0.025,0.043] | <0.001 | 0.032 | [0.022,0.042] | <0.001 |
| Age^2^ | 0.000 | [0.000,0.001] | <0.001 | 0.000 | [0.000,0.001] | <0.001 | 0.000 | [0.000,0.001] | <0.001 |
| Ref: Highest wealth |  |  |  |  |  | <0.001 |  |  | <0.001 |
| Higher wealth | 0.179 | [0.130,0.229] | <0.001 | 0.155 | [0.104,0.205] | <0.001 | 0.153 | [0.102,0.203] | <0.001 |
| Lower wealth | 0.345 | [0.281,0.410] | <0.001 | 0.301 | [0.234,0.368] | <0.001 | 0.296 | [0.228,0.363] | <0.001 |
| Lowest wealth | 0.444 | [0.384,0.504] | <0.001 | 0.381 | [0.315,0.447] | <0.001 | 0.371 | [0.303,0.439] | <0.001 |
| Ref: Highest wealth x Age |  |  |  |  |  |  |  |  |  |
| Higher wealth x Age | 0.001 | [-0.005,0.007] | 0.701 | 0.001 | [-0.005,0.006] | 0.856 | 0.001 | [-0.005,0.006] | 0.813 |
| Lower wealth x Age | -0.002 | [-0.009,0.006] | 0.653 | -0.003 | [-0.010,0.005] | 0.484 | -0.002 | [-0.010,0.005] | 0.543 |
| Lowest wealth x Age | -0.002 | [-0.008,0.004] | 0.548 | -0.003 | [-0.010,0.003] | 0.334 | -0.003 | [-0.010,0.004] | 0.441 |
| Observations | 14,400 |  |  |  |  |  |  |  |  |
| **Self-rated eyesight** |  |  |  |  |  |  |  |  |  |
| Age | 0.015 | [0.008,0.022] | <0.001 | 0.008 | [-0.002,0.019] | 0.124 | 0.008 | [-0.003,0.019] | 0.144 |
| Age^2^ | 0.001 | [0.001,0.001] | <0.001 | 0.001 | [0.001,0.001] | <0.001 | 0.001 | [0.001,0.001] | <0.001 |
| Ref: Highest wealth |  |  |  |  |  |  |  |  |  |
| Higher wealth | 0.101 | [0.039,0.163] | 0.001 | 0.075 | [0.013,0.138] | 0.018 | 0.075 | [0.012,0.137] | 0.019 |
| Lower wealth | 0.380 | [0.304,0.457] | <0.001 | 0.324 | [0.246,0.402] | <0.001 | 0.324 | [0.246,0.402] | <0.001 |
| Lowest wealth | 0.530 | [0.458,0.602] | <0.001 | 0.448 | [0.371,0.525] | <0.001 | 0.449 | [0.371,0.528] | <0.001 |
| Ref: Highest wealth x Age |  |  |  |  |  |  |  |  |  |
| Higher wealth x Age | 0.001 | [-0.006,0.008] | 0.731 | 0.002 | [-0.005,0.009] | 0.591 | 0.002 | [-0.005,0.009] | 0.597 |
| Lower wealth x Age | 0.000 | [-0.008,0.008] | 0.944 | 0.002 | [-0.007,0.010] | 0.705 | 0.002 | [-0.007,0.010] | 0.713 |
| Lowest wealth x Age | -0.001 | [-0.009,0.007] | 0.819 | 0.001 | [-0.007,0.010] | 0.737 | 0.001 | [-0.007,0.010] | 0.759 |
| Observations | 24,899 |  |  |  |  |  |  |  |  |
| **Self-rated hearing** |  |  |  |  |  |  |  |  |  |
| Age | 0.028 | [0.021,0.035] | <0.001 | 0.024 | [0.013,0.035] | <0.001 | 0.025 | [0.013,0.036] | <0.001 |
| Age^2^ | 0.000 | [0.000,0.001] | <0.001 | 0.001 | [0.000,0.001] | <0.001 | 0.001 | [0.000,0.001] | <0.001 |
| Ref: Highest wealth |  |  |  |  |  |  |  |  |  |
| Higher wealth | 0.071 | [-0.002,0.145] | 0.058 | 0.067 | [-0.008,0.141] | 0.079 | 0.067 | [-0.007,0.142] | 0.077 |
| Lower wealth | 0.261 | [0.170,0.353] | <0.001 | 0.247 | [0.153,0.341] | <0.001 | 0.249 | [0.155,0.343] | <0.001 |
| Lowest wealth | 0.239 | [0.153,0.324] | <0.001 | 0.217 | [0.127,0.308] | <0.001 | 0.222 | [0.129,0.315] | <0.001 |
| Ref: Highest wealth x Age |  |  |  |  |  |  |  |  |  |
| Higher wealth x Age | -0.009 | [-0.016,-0.001] | 0.019 | -0.008 | [-0.016,-0.001] | 0.025 | -0.008 | [-0.015,-0.001] | 0.028 |
| Lower wealth x Age | -0.010 | [-0.018,-0.001] | 0.028 | -0.009 | [-0.017,0.000] | 0.060 | -0.008 | [-0.017,0.001] | 0.072 |
| Lowest wealth x Age | -0.004 | [-0.013,0.004] | 0.290 | -0.003 | [-0.012,0.006] | 0.519 | -0.002 | [-0.012,0.007] | 0.591 |
| Observations | 24,901 |  |  |  |  |  |  |  |  |
| **ADL difficulties, number square root** |  |  |  |  |  |  |  |  |  |
| Age | 0.008 | [0.005,0.012] | <0.001 | 0.007 | [0.000,0.013] | 0.049 | 0.007 | [0.000,0.013] | 0.048 |
| Age^2^ | 0.001 | [0.001,0.001] | <0.001 | 0.001 | [0.001,0.001] | <0.001 | 0.001 | [0.001,0.001] | <0.001 |
| Ref: Highest wealth |  |  |  |  |  |  |  |  |  |
| Higher wealth | 0.070 | [0.042,0.097] | <0.001 | 0.050 | [0.023,0.077] | <0.001 | 0.048 | [0.022,0.075] | <0.001 |
| Lower wealth | 0.184 | [0.141,0.228] | <0.001 | 0.140 | [0.097,0.182] | <0.001 | 0.136 | [0.094,0.179] | <0.001 |
| Lowest wealth | 0.336 | [0.295,0.378] | <0.001 | 0.267 | [0.224,0.310] | <0.001 | 0.261 | [0.217,0.305] | <0.001 |
| Ref: Highest wealth x Age |  |  |  |  |  |  |  |  |  |
| Higher wealth x Age | 0.002 | [-0.001,0.006] | 0.201 | 0.003 | [-0.001,0.006] | 0.153 | 0.003 | [-0.001,0.007] | 0.136 |
| Lower wealth x Age | 0.009 | [0.004,0.014] | 0.001 | 0.009 | [0.004,0.014] | <0.001 | 0.010 | [0.005,0.015] | <0.001 |
| Lowest wealth x Age | 0.007 | [0.003,0.012] | 0.003 | 0.009 | [0.004,0.014] | 0.001 | 0.009 | [0.004,0.015] | <0.001 |
| Observations | 24,904 |  |  |  |  |  |  |  |  |
| **IADL difficulties, number square root** |  |  |  |  |  |  |  |  |  |
| Age | 0.006 | [0.002,0.010] | 0.003 | 0.008 | [0.001,0.015] | 0.021 | 0.008 | [0.001,0.015] | 0.029 |
| Age^2^ | 0.002 | [0.001,0.002] | <0.001 | 0.002 | [0.002,0.002] | <0.001 | 0.002 | [0.002,0.002] | <0.001 |
| Ref: Highest wealth |  |  |  |  |  |  |  |  |  |
| Higher wealth | 0.050 | [0.019,0.082] | 0.002 | 0.038 | [0.008,0.069] | 0.013 | 0.036 | [0.006,0.067] | 0.019 |
| Lower wealth | 0.155 | [0.109,0.201] | <0.001 | 0.122 | [0.077,0.168] | <0.001 | 0.117 | [0.072,0.163] | <0.001 |
| Lowest wealth | 0.318 | [0.272,0.364] | <0.001 | 0.268 | [0.221,0.315] | <0.001 | 0.259 | [0.211,0.307] | <0.001 |
| Ref: Highest wealth x Age |  |  |  |  |  |  |  |  |  |
| Higher wealth x Age | 0.004 | [0.000,0.008] | 0.046 | 0.004 | [0.000,0.008] | 0.042 | 0.004 | [0.000,0.008] | 0.072 |
| Lower wealth x Age | 0.012 | [0.007,0.017] | <0.001 | 0.012 | [0.007,0.018] | <0.001 | 0.011 | [0.006,0.017] | <0.001 |
| Lowest wealth x Age | 0.014 | [0.009,0.019] | <0.001 | 0.015 | [0.009,0.020] | <0.001 | 0.013 | [0.007,0.019] | <0.001 |
| Observations | 24,904 |  |  |  |  |  |  |  |  |
| **Mobility difficulties, number** |  |  |  |  |  |  |  |  |  |
| Age | 0.034 | [0.020,0.049] | <0.001 | 0.013 | [-0.013,0.039] | 0.322 | 0.013 | [-0.013,0.039] | 0.324 |
| Age^2^ | 0.004 | [0.003,0.004] | <0.001 | 0.004 | [0.004,0.005] | 0.000 | 0.004 | [0.004,0.005] | <0.001 |
| Ref: Highest wealth |  |  |  |  |  |  |  |  |  |
| Higher wealth | 0.386 | [0.241,0.531] | <0.001 | 0.297 | [0.158,0.436] | 0.000 | 0.290 | [0.151,0.429] | <0.001 |
| Lower wealth | 1.181 | [0.962,1.400] | <0.001 | 0.978 | [0.765,1.192] | 0.000 | 0.963 | [0.748,1.177] | <0.001 |
| Lowest wealth | 2.156 | [1.950,2.362] | <0.001 | 1.865 | [1.657,2.074] | 0.000 | 1.837 | [1.625,2.050] | <0.001 |
| Ref: Highest wealth x Age |  |  |  |  |  |  |  |  |  |
| Higher wealth x Age | 0.015 | [0.000,0.030] | 0.053 | 0.018 | [0.003,0.032] | 0.022 | 0.017 | [0.002,0.033] | 0.023 |
| Lower wealth x Age | 0.023 | [0.003,0.042] | 0.026 | 0.028 | [0.008,0.048] | 0.006 | 0.028 | [0.008,0.048] | 0.007 |
| Lowest wealth x Age | 0.025 | [0.006,0.045] | 0.011 | 0.031 | [0.011,0.052] | 0.003 | 0.031 | [0.010,0.053] | 0.004 |
| Observations | 24,905 |  |  |  |  |  |  |  |  |

ELSA=English Longitudinal Study of Ageing; CLSA=Canadian Longitudinal Study on Aging; FEV=Forced Expiratory Volume; ADL=basic activities of daily living; IADL=instrumental activities of daily living. ^1^For measures where higher values originally indicated better function (gait speed, grip strength, and lung function), we reversed the direction by multiplying by −1. This transformation does not affect model fit or statistical inference but ensures consistent interpretation across outcomes. Model 1 included age^2^, birth year, sex, race, and height (gait speed and grip strength analyses only) as covariates; model 2 additionally included smoking, alcohol consumption, weight status, and chronic conditions; model 3 additionally included marital status, social isolation, and loneliness. For wealth associations, the highest category was the reference group.
