# Supplementary material for: Socioeconomic inequalities in functional ageing trajectories in England and Canada: A comparative longitudinal cohort study
Source: PLoS Med. 2026 Aug 13;23(8):e1004833. doi: 10.1371/journal.pmed.1004833 (PMC13472442; doi:10.1371/journal.pmed.1004833)
Supplement: Supplementary file 7 [file pmed.1004833.s007.docx]

**S6 Table. Associations between wealth and the level of and change in functional health outcomes in CLSA**

|  | **Model 1** | | | **Model 2** | | | **Model 3** | | |
| --- | --- | --- | --- | --- | --- | --- | --- | --- | --- |
|  | b | 95% CI | p | b | 95% CI | p | b | 95% CI | p |
| **Gait speed, m/s^1^** |  |  |  |  |  |  |  |  |  |
| Age | -0.005 | [-0.018,0.009] | 0.496 | -0.006 | [-0.020,0.008] | 0.386 | -0.006 | [-0.020,0.007] | 0.368 |
| Age^2^ | 0.000 | [0.000,0.000] | <0.001 | 0.000 | [0.000,0.000] | <0.001 | 0.000 | [0.000,0.000] | <0.001 |
| Ref: Highest wealth |  |  |  |  |  |  |  |  |  |
| Higher wealth | 0.016 | [0.003,0.030] | 0.017 | 0.010 | [-0.003,0.023] | 0.145 | 0.009 | [-0.004,0.022] | 0.171 |
| Lower wealth | 0.047 | [0.030,0.065] | <0.001 | 0.037 | [0.019,0.054] | <0.001 | 0.036 | [0.019,0.053] | <0.001 |
| Lowest wealth | 0.079 | [0.064,0.095] | <0.001 | 0.061 | [0.045,0.077] | <0.001 | 0.059 | [0.043,0.075] | <0.001 |
| Ref: Highest wealth x Age |  |  |  |  |  |  |  |  |  |
| Higher wealth x Age | -0.001 | [-0.002,0.001] | 0.478 | 0.000 | [-0.002,0.001] | 0.627 | 0.000 | [-0.002,0.001] | 0.596 |
| Lower wealth x Age | -0.001 | [-0.003,0.001] | 0.214 | -0.001 | [-0.003,0.001] | 0.341 | -0.001 | [-0.003,0.001] | 0.298 |
| Lowest wealth x Age | -0.001 | [-0.003,0.001] | 0.208 | -0.001 | [-0.003,0.001] | 0.376 | -0.001 | [-0.003,0.001] | 0.305 |
| Observations | 33,896 |  |  |  |  |  |  |  |  |
| **Grip strength, kg^1^** |  |  |  |  |  |  |  |  |  |
| Age | -0.442 | [-0.778,-0.106] | 0.010 | -0.492 | [-0.828,-0.156] | 0.004 | -0.474 | [-0.810,-0.138] | 0.006 |
| Age^2^ | 0.008 | [0.007,0.009] | <0.001 | 0.008 | [0.007,0.009] | <0.001 | 0.008 | [0.007,0.009] | <0.001 |
| Ref: Highest wealth |  |  |  |  |  |  |  |  |  |
| Higher wealth | 0.403 | [0.057,0.749] | 0.023 | 0.393 | [0.049,0.737] | 0.025 | 0.364 | [0.020,0.708] | 0.038 |
| Lower wealth | 0.838 | [0.418,1.258] | <0.001 | 0.818 | [0.399,1.236] | <0.001 | 0.771 | [0.352,1.190] | <0.001 |
| Lowest wealth | 1.526 | [1.121,1.930] | <0.001 | 1.424 | [1.016,1.833] | <0.001 | 1.328 | [0.915,1.741] | <0.001 |
| Ref: Highest wealth x Age |  |  |  |  |  |  |  |  |  |
| Higher wealth x Age | -0.001 | [-0.042,0.039] | 0.951 | -0.003 | [-0.043,0.038] | 0.893 | -0.001 | [-0.042,0.039] | 0.957 |
| Lower wealth x Age | 0.032 | [-0.014,0.079] | 0.173 | 0.030 | [-0.017,0.076] | 0.212 | 0.032 | [-0.015,0.079] | 0.178 |
| Lowest wealth x Age | 0.034 | [-0.012,0.080] | 0.152 | 0.028 | [-0.019,0.075] | 0.239 | 0.032 | [-0.015,0.079] | 0.177 |
| Observations | 49,543 |  |  |  |  |  |  |  |  |
| **Chair rise time, log s** |  |  |  |  |  |  |  |  |  |
| Age | 0.000 | [-0.002,0.001] | 0.586 | -0.001 | [-0.003,0.001] | 0.510 | -0.001 | [-0.003,0.002] | 0.555 |
| Age^2^ | 0.000 | [0.000,0.000] | <0.001 | 0.000 | [0.000,0.000] | <0.001 | 0.000 | [0.000,0.000] | <0.001 |
| Ref: Highest wealth |  |  |  |  |  |  |  |  |  |
| Higher wealth | 0.037 | [0.025,0.048] | <0.001 | 0.031 | [0.020,0.042] | <0.001 | 0.030 | [0.019,0.041] | <0.001 |
| Lower wealth | 0.060 | [0.046,0.074] | <0.001 | 0.049 | [0.035,0.063] | <0.001 | 0.048 | [0.034,0.062] | <0.001 |
| Lowest wealth | 0.107 | [0.094,0.121] | <0.001 | 0.089 | [0.075,0.103] | <0.001 | 0.087 | [0.074,0.101] | <0.001 |
| Ref: Highest wealth x Age |  |  |  |  |  |  |  |  |  |
| Higher wealth x Age | -0.002 | [-0.004,-0.001] | 0.003 | -0.002 | [-0.004,-0.001] | 0.006 | -0.002 | [-0.004,-0.001] | 0.008 |
| Lower wealth x Age | -0.004 | [-0.005,-0.002] | <0.001 | -0.003 | [-0.005,-0.001] | <0.001 | -0.003 | [-0.005,-0.001] | <0.001 |
| Lowest wealth x Age | -0.003 | [-0.005,-0.002] | <0.001 | -0.003 | [-0.005,-0.001] | 0.001 | -0.003 | [-0.005,-0.001] | 0.003 |
| Observations | 51,252 |  |  |  |  |  |  |  |  |
| **FEV, litres^1^** |  |  |  |  |  |  |  |  |  |
| Age | 0.036 | [0.034,0.039] | <0.001 | 0.035 | [0.032,0.038] | <0.001 | 0.035 | [0.032,0.038] | <0.001 |
| Age^2^ | 0.000 | [0.000,0.000] | <0.001 | 0.000 | [0.000,0.000] | <0.001 | 0.000 | [0.000,0.000] | <0.001 |
| Ref: Highest wealth |  |  |  |  |  |  |  |  |  |
| Higher wealth | 0.072 | [0.045,0.099] | <0.001 | 0.059 | [0.032,0.086] | <0.001 | 0.059 | [0.032,0.086] | <0.001 |
| Lower wealth | 0.167 | [0.135,0.200] | <0.001 | 0.144 | [0.112,0.177] | <0.001 | 0.144 | [0.112,0.177] | <0.001 |
| Lowest wealth | 0.258 | [0.226,0.289] | <0.001 | 0.223 | [0.192,0.255] | <0.001 | 0.223 | [0.191,0.254] | <0.001 |
| Ref: Highest wealth x Age |  |  |  |  |  |  |  |  |  |
| Higher wealth x Age | -0.001 | [-0.003,0.001] | 0.456 | -0.001 | [-0.003,0.002] | 0.511 | -0.001 | [-0.003,0.002] | 0.475 |
| Lower wealth x Age | -0.002 | [-0.004,0.001] | 0.283 | -0.001 | [-0.004,0.002] | 0.396 | -0.001 | [-0.004,0.001] | 0.349 |
| Lowest wealth x Age | -0.001 | [-0.003,0.002] | 0.678 | 0.000 | [-0.003,0.003] | 0.999 | 0.000 | [-0.003,0.003] | 0.886 |
| Observations | 38,097 |  |  |  |  |  |  |  |  |
| **Self-rated eyesight** |  |  |  |  |  |  |  |  |  |
| Age | 0.018 | [0.014,0.023] | <0.001 | 0.019 | [0.012,0.026] | <0.001 | 0.020 | [0.013,0.027] | <0.001 |
| Age^2^ | 0.000 | [0.000,0.000] | 0.046 | 0.000 | [0.000,0.000] | 0.103 | 0.000 | [0.000,0.000] | 0.071 |
| Ref: Highest wealth |  |  |  |  |  |  |  |  |  |
| Higher wealth | 0.075 | [0.041,0.109] | <0.001 | 0.066 | [0.032,0.101] | <0.001 | 0.063 | [0.029,0.098] | <0.001 |
| Lower wealth | 0.143 | [0.102,0.185] | <0.001 | 0.127 | [0.085,0.169] | <0.001 | 0.121 | [0.079,0.163] | <0.001 |
| Lowest wealth | 0.300 | [0.260,0.339] | <0.001 | 0.266 | [0.225,0.308] | <0.001 | 0.256 | [0.214,0.298] | <0.001 |
| Ref: Highest wealth x Age |  |  |  |  |  |  |  |  |  |
| Higher wealth x Age | -0.001 | [-0.006,0.003] | 0.543 | -0.001 | [-0.005,0.003] | 0.63 | -0.001 | [-0.005,0.003] | 0.667 |
| Lower wealth x Age | -0.005 | [-0.010,0.000] | 0.065 | -0.004 | [-0.009,0.001] | 0.125 | -0.004 | [-0.009,0.001] | 0.159 |
| Lowest wealth x Age | -0.008 | [-0.012,-0.003] | 0.003 | -0.006 | [-0.011,-0.001] | 0.019 | -0.005 | [-0.011,0.000] | 0.038 |
| Observations | 63,276 |  |  |  |  |  |  |  |  |
| **Self-rated hearing** |  |  |  |  |  |  |  |  |  |
| Age | 0.037 | [0.032,0.042] | <0.001 | 0.040 | [0.032,0.047] | <0.001 | 0.041 | [0.034,0.049] | <0.001 |
| Age^2^ | 0.000 | [0.000,0.000] | 0.952 | 0.000 | [0.000,0.000] | 0.915 | 0.000 | [0.000,0.000] | 0.726 |
| Ref: Highest wealth |  |  |  |  |  |  |  |  |  |
| Higher wealth | 0.058 | [0.018,0.098] | 0.004 | 0.047 | [0.007,0.087] | 0.021 | 0.046 | [0.006,0.086] | 0.023 |
| Lower wealth | 0.071 | [0.023,0.120] | 0.004 | 0.053 | [0.004,0.102] | 0.034 | 0.051 | [0.001,0.100] | 0.044 |
| Lowest wealth | 0.134 | [0.087,0.180] | <0.001 | 0.105 | [0.057,0.152] | <0.001 | 0.101 | [0.051,0.150] | <0.001 |
| Ref: Highest wealth x Age |  |  |  |  |  |  |  |  |  |
| Higher wealth x Age | -0.001 | [-0.006,0.004] | 0.683 | -0.001 | [-0.005,0.004] | 0.825 | -0.001 | [-0.005,0.004] | 0.762 |
| Lower wealth x Age | -0.004 | [-0.010,0.001] | 0.113 | -0.004 | [-0.009,0.002] | 0.192 | -0.004 | [-0.009,0.002] | 0.187 |
| Lowest wealth x Age | -0.004 | [-0.009,0.001] | 0.142 | -0.003 | [-0.008,0.003] | 0.332 | -0.003 | [-0.009,0.003] | 0.328 |
| Observations | 63,229 |  |  |  |  |  |  |  |  |
| **ADL difficulties, number square root** |  |  |  |  |  |  |  |  |  |
| Age | 0.011 | [0.009,0.013] | <0.001 | 0.012 | [0.009,0.016] | <0.001 | 0.012 | [0.009,0.015] | <0.001 |
| Age^2^ | 0.000 | [0.000,0.001] | <0.001 | 0.000 | [0.000,0.000] | <0.001 | 0.000 | [0.000,0.000] | <0.001 |
| Ref: Highest wealth |  |  |  |  |  |  |  |  |  |
| Higher wealth | 0.026 | [0.014,0.038] | <0.001 | 0.017 | [0.005,0.029] | 0.005 | 0.016 | [0.004,0.027] | 0.010 |
| Lower wealth | 0.060 | [0.043,0.076] | <0.001 | 0.044 | [0.028,0.060] | <0.001 | 0.041 | [0.025,0.057] | <0.001 |
| Lowest wealth | 0.129 | [0.112,0.146] | <0.001 | 0.100 | [0.082,0.118] | <0.001 | 0.096 | [0.078,0.113] | <0.001 |
| Ref: Highest wealth x Age |  |  |  |  |  |  |  |  |  |
| Higher wealth x Age | 0.000 | [-0.002,0.002] | 0.929 | 0.000 | [-0.002,0.002] | 0.972 | 0.000 | [-0.002,0.002] | 0.970 |
| Lower wealth x Age | 0.002 | [0.000,0.004] | 0.041 | 0.002 | [0.000,0.004] | 0.059 | 0.002 | [0.000,0.004] | 0.062 |
| Lowest wealth x Age | 0.003 | [0.001,0.005] | 0.006 | 0.003 | [0.000,0.005] | 0.019 | 0.002 | [0.000,0.005] | 0.025 |
| Observations | 64,095 |  |  |  |  |  |  |  |  |
| **IADL difficulties, number square root** |  |  |  |  |  |  |  |  |  |
| Age | 0.011 | [0.009,0.012] | <0.001 | 0.014 | [0.010,0.017] | <0.001 | 0.013 | [0.010,0.017] | <0.001 |
| Age^2^ | 0.001 | [0.001,0.001] | <0.001 | 0.001 | [0.001,0.001] | <0.001 | 0.001 | [0.001,0.001] | <0.001 |
| Ref: Highest wealth |  |  |  |  |  |  |  |  |  |
| Higher wealth | 0.005 | [-0.003,0.013] | 0.244 | -0.002 | [-0.010,0.006] | 0.680 | -0.003 | [-0.011,0.006] | 0.536 |
| Lower wealth | 0.014 | [0.002,0.025] | 0.019 | 0.002 | [-0.010,0.014] | 0.799 | 0.000 | [-0.012,0.012] | 0.999 |
| Lowest wealth | 0.071 | [0.059,0.084] | <0.001 | 0.047 | [0.034,0.061] | <0.001 | 0.044 | [0.031,0.058] | <0.001 |
| Ref: Highest wealth x Age |  |  |  |  |  |  |  |  |  |
| Higher wealth x Age | 0.001 | [0.000,0.002] | 0.105 | 0.001 | [-0.001,0.002] | 0.246 | 0.001 | [-0.001,0.002] | 0.316 |
| Lower wealth x Age | 0.003 | [0.002,0.005] | <0.001 | 0.003 | [0.001,0.004] | 0.002 | 0.002 | [0.001,0.004] | 0.005 |
| Lowest wealth x Age | 0.004 | [0.003,0.006] | <0.001 | 0.003 | [0.002,0.005] | <0.001 | 0.003 | [0.001,0.005] | 0.001 |
| Observations | 64,095 |  |  |  |  |  |  |  |  |
| **Up and go time, log s** |  |  |  |  |  |  |  |  |  |
| Age | 0.016 | [0.015,0.018] | <0.001 | 0.016 | [0.014,0.017] | <0.001 | 0.015 | [0.014,0.017] | <0.001 |
| Age^2^ | 0.000 | [0.000,0.000] | <0.001 | 0.000 | [0.000,0.000] | <0.001 | 0.000 | [0.000,0.000] | <0.001 |
| Ref: Highest wealth |  |  |  |  |  |  |  |  |  |
| Higher wealth | 0.013 | [0.004,0.022] | 0.005 | 0.007 | [-0.002,0.016] | 0.122 | 0.006 | [-0.003,0.014] | 0.199 |
| Lower wealth | 0.038 | [0.026,0.050] | <0.001 | 0.028 | [0.016,0.039] | <0.001 | 0.026 | [0.015,0.037] | <0.001 |
| Lowest wealth | 0.085 | [0.074,0.096] | <0.001 | 0.067 | [0.056,0.078] | <0.001 | 0.063 | [0.052,0.074] | <0.001 |
| Ref: Highest wealth x Age |  |  |  |  |  |  |  |  |  |
| Higher wealth x Age | 0.001 | [0.000,0.002] | 0.032 | 0.001 | [0.000,0.002] | 0.033 | 0.001 | [0.000,0.002] | 0.043 |
| Lower wealth x Age | 0.001 | [-0.001,0.002] | 0.224 | 0.001 | [-0.001,0.002] | 0.225 | 0.001 | [-0.001,0.002] | 0.307 |
| Lowest wealth x Age | 0.002 | [0.001,0.004] | 0.001 | 0.002 | [0.001,0.004] | 0.002 | 0.002 | [0.001,0.003] | 0.006 |
| Observations | 53,498 |  |  |  |  |  |  |  |  |

ELSA=English Longitudinal Study of Ageing; CLSA=Canadian Longitudinal Study on Aging; FEV=Forced Expiratory Volume; ADL=basic activities of daily living; IADL=instrumental activities of daily living. ^1^For measures where higher values originally indicated better function (gait speed, grip strength, and lung function), we reversed the direction by multiplying by −1. This transformation does not affect model fit or statistical inference but ensures consistent interpretation across outcomes. Model 1 included age^2^, birth year, sex, race, and height (gait speed and grip strength analyses only) as covariates; model 2 additionally included smoking, alcohol consumption, weight status, and chronic conditions; model 3 additionally included marital status, social isolation, and loneliness. For wealth associations, the highest category was the reference group.
