# Supplementary material for: Socioeconomic inequalities in functional ageing trajectories in England and Canada: A comparative longitudinal cohort study
Source: PLoS Med. 2026 Aug 13;23(8):e1004833. doi: 10.1371/journal.pmed.1004833 (PMC13472442; doi:10.1371/journal.pmed.1004833)
Supplement: Supplementary file 8 [file pmed.1004833.s008.docx]

**S7 Table. Associations between education and the level of and change in functional health outcomes in the two cohorts**

|  | **ELSA** | | | **CLSA** | | |
| --- | --- | --- | --- | --- | --- | --- |
|  | b | 95% CI | p | b | 95% CI | p |
| **Gait speed, m/s^1^** |  |  |  |  |  |  |
| Age | 0.012 | [-0.010,0.034] | 0.298 | -0.008 | [-0.022,0.006] | 0.248 |
| Age^2^ | 0.001 | [0.000,0.001] | <0.001 | 0.000 | [0.000,0.000] | <0.001 |
| Ref: Tertiary |  |  |  |  |  |  |
| Secondary | 0.050 | [0.030,0.070] | <0.001 | 0.022 | [0.010,0.033] | <0.001 |
| Primary | 0.148 | [0.127,0.169] | <0.001 | 0.073 | [0.050,0.097] | <0.001 |
| Ref: Tertiary x Age |  |  |  |  |  |  |
| Secondary x Age | -0.001 | [-0.003,0.001] | 0.385 | 0.000 | [-0.001,0.002] | 0.536 |
| Primary x Age | -0.003 | [-0.005,-0.001] | 0.001 | -0.002 | [-0.004,0.000] | 0.095 |
| Observations | 14,711 |  |  | 33,818 |  |  |
| **Grip strength, kg^1^** |  |  |  |  |  |  |
| Age | 0.541 | [-2.584,3.667] | 0.734 | -0.394 | [-0.723,-0.065] | 0.019 |
| Age^2^ | 0.006 | [-0.025,0.037] | 0.706 | 0.008 | [0.007,0.009] | <0.001 |
| Ref: Tertiary |  |  |  |  |  |  |
| Secondary | 0.995 | [0.346,1.643] | 0.003 | 0.336 | [0.046,0.626] | 0.023 |
| Primary | 1.785 | [0.999,2.571] | <0.001 | 0.590 | [-0.036,1.215] | 0.065 |
| Ref: Tertiary x Age |  |  |  |  |  |  |
| Secondary x Age | 0.009 | [-0.069,0.088] | 0.818 | 0.030 | [-0.002,0.062] | 0.069 |
| Primary x Age | -0.052 | [-0.273,0.169] | 0.643 | 0.018 | [-0.040,0.075] | 0.545 |
| Observations | 11,977 |  |  | 49,464 |  |  |
| **Chair rise time, log s** |  |  |  |  |  |  |
| Age | 0.005 | [0.003,0.008] | <0.001 | -0.003 | [-0.004,-0.002] | <0.001 |
| Age^2^ | 0.000 | [0.000,0.000] | <0.001 | 0.000 | [0.000,0.000] | <0.001 |
| Ref: Tertiary |  |  |  |  |  |  |
| Secondary | 0.039 | [0.017,0.061] | <0.001 | 0.029 | [0.019,0.039] | <0.001 |
| Primary | 0.077 | [0.056,0.097] | <0.001 | 0.079 | [0.058,0.101] | <0.001 |
| Ref: Tertiary x Age |  |  |  |  |  |  |
| Secondary x Age | 0.001 | [-0.002,0.003] | 0.687 | -0.000 | [-0.002,0.001] | 0.427 |
| Primary x Age | 0.001 | [-0.002,0.003] | 0.497 | -0.002 | [-0.005,0.000] | 0.051 |
| Observations | 13,247 |  |  | 51,177 |  |  |
| **FEV, litres^1^** |  |  |  |  |  |  |
| Age | 0.032 | [0.027,0.037] | <0.001 | 0.035 | [0.034,0.037] | <0.001 |
| Age^2^ | 0.000 | [0.000,0.001] | <0.001 | 0.000 | [0.000,0.000] | <0.001 |
| Ref: Tertiary |  |  |  |  |  |  |
| Secondary | 0.105 | [0.058,0.153] | <0.001 | 0.075 | [0.052,0.098] | <0.001 |
| Primary | 0.265 | [0.221,0.309] | <0.001 | 0.228 | [0.178,0.278] | <0.001 |
| Ref: Tertiary x Age |  |  |  |  |  |  |
| Secondary x Age | 0.003 | [-0.002,0.008] | 0.262 | 0.001 | [-0.001,0.003] | 0.322 |
| Primary x Age | -0.002 | [-0.007,0.002] | 0.339 | -0.004 | [-0.008,-0.000] | 0.046 |
| Observations | 14,392 |  |  | 38,039 |  |  |
| **Self-rated eyesight** |  |  |  |  |  |  |
| Age | 0.018 | [0.014,0.023] | <0.001 | 0.016 | [0.013,0.020] | <0.001 |
| Age^2^ | 0.001 | [0.001,0.001] | <0.001 | 0.000 | [0.000,0.000] | 0.017 |
| Ref: Tertiary |  |  |  |  |  |  |
| Secondary | 0.147 | [0.100,0.195] | <0.001 | 0.074 | [0.044,0.103] | <0.001 |
| Primary | 0.369 | [0.317,0.422] | <0.001 | 0.283 | [0.221,0.346] | <0.001 |
| Ref: Tertiary x Age |  |  |  |  |  |  |
| Secondary x Age | -0.004 | [-0.009,0.001] | 0.145 | -0.002 | [-0.006,0.001] | 0.172 |
| Primary x Age | -0.009 | [-0.014,-0.004] | 0.001 | -0.007 | [-0.013,-0.001] | 0.032 |
| Observations | 24,878 |  |  | 63,177 |  |  |
| **Self-rated hearing** |  |  |  |  |  |  |
| Age | 0.023 | [0.018,0.028] | <0.001 | 0.036 | [0.033,0.039] | <0.001 |
| Age^2^ | 0.001 | [0.000,0.001] | <0.001 | 0.000 | [-0.000,0.000] | 0.802 |
| Ref: Tertiary |  |  |  |  |  |  |
| Secondary | 0.076 | [0.020,0.133] | 0.008 | 0.045 | [0.010,0.079] | 0.011 |
| Primary | 0.234 | [0.171,0.296] | <0.001 | 0.172 | [0.097,0.247] | <0.001 |
| Ref: Tertiary x Age |  |  |  |  |  |  |
| Secondary x Age | -0.002 | [-0.008,0.004] | 0.470 | -0.002 | [-0.006,0.002] | 0.355 |
| Primary x Age | -0.005 | [-0.011,0.001] | 0.086 | -0.009 | [-0.016,-0.002] | 0.017 |
| Observations | 24,880 |  |  | 63,130 |  |  |
| **ADL difficulties, number square root** |  |  |  |  |  |  |
| Age | 0.011 | [0.008,0.014] | <0.001 | 0.012 | [0.011,0.013] | <0.001 |
| Age^2^ | 0.001 | [0.001,0.001] | <0.001 | 0.000 | [0.000,0.001] | <0.001 |
| Ref: Tertiary |  |  |  |  |  |  |
| Secondary | 0.060 | [0.031,0.089] | <0.001 | 0.031 | [0.017,0.044] | <0.001 |
| Primary | 0.156 | [0.123,0.190] | <0.001 | 0.070 | [0.038,0.101] | <0.001 |
| Ref: Tertiary x Age |  |  |  |  |  |  |
| Secondary x Age | 0.003 | [-0.000,0.006] | 0.090 | -0.001 | [-0.003,0.001] | 0.276 |
| Primary x Age | 0.000 | [-0.003,0.003] | 0.967 | -0.002 | [-0.005,0.001] | 0.209 |
| Observations | 24,883 |  |  | 63,993 |  |  |
| **IADL difficulties, number square root** |  |  |  |  |  |  |
| Age | 0.011 | [0.008,0.013] | <0.001 | 0.012 | [0.011,0.013] | <0.001 |
| Age^2^ | 0.002 | [0.001,0.002] | <0.001 | 0.001 | [0.001,0.001] | <0.001 |
| Ref: Tertiary |  |  |  |  |  |  |
| Secondary | 0.058 | [0.028,0.088] | <0.001 | 0.013 | [0.004,0.023] | 0.006 |
| Primary | 0.162 | [0.126,0.198] | <0.001 | 0.039 | [0.016,0.061] | 0.001 |
| Ref: Tertiary x Age |  |  |  |  |  |  |
| Secondary x Age | 0.003 | [-0.000,0.007] | 0.053 | 0.001 | [0.000,0.003] | 0.034 |
| Primary x Age | 0.004 | [0.001,0.008] | 0.024 | 0.001 | [-0.002,0.003] | 0.602 |
| Observations | 24,883 |  |  | 63,993 |  |  |
| **Mobility difficulties, number** |  |  |  |  |  |  |
| Age | 0.046 | [0.036,0.056] | <0.001 |  |  |  |
| Age^2^ | 0.004 | [0.003,0.004] | <0.001 |  |  |  |
| Ref: Tertiary |  |  |  |  |  |  |
| Secondary | 0.405 | [0.263,0.547] | <0.001 | - | - | - |
| Primary | 1.095 | [0.931,1.258] | <0.001 | - | - | - |
| Ref: Tertiary x Age |  |  |  |  |  |  |
| Secondary x Age | 0.008 | [-0.005,0.021] | 0.220 | - | - | - |
| Primary x Age | 0.002 | [-0.012,0.016] | 0.775 | - | - | - |
| Observations | 24,884 |  |  | - |  |  |
| **Up and go, log s** |  |  |  |  |  |  |
| Age |  |  |  | 0.018 | [0.017,0.018] | <0.001 |
| Age^2^ |  |  |  | 0.000 | [0.000,0.000] | <0.001 |
| Ref: Tertiary |  |  |  |  |  |  |
| Secondary | - | - | - | 0.032 | [0.024,0.040] | <0.001 |
| Primary | - | - | - | 0.078 | [0.056,0.100] | <0.001 |
| Ref: Tertiary x Age |  |  |  |  |  |  |
| Secondary x Age | - | - | - | 0.000 | [-0.001,0.001] | 0.966 |
| Primary x Age | - | - | - | -0.000 | [-0.002,0.002] | 0.994 |
| Observations | - |  |  | 53,417 |  |  |

ELSA=English Longitudinal Study of Ageing; CLSA=Canadian Longitudinal Study on Aging; FEV=Forced Expiratory Volume; ADL=basic activities of daily living; IADL=instrumental activities of daily living. ^1^For measures where higher values originally indicated better function (gait speed, grip strength, and lung function), we reversed the direction by multiplying by −1. This transformation does not affect model fit or statistical inference but ensures consistent interpretation across outcomes. Models included age^2^, birth year, sex, race, and height (gait speed and grip strength analyses only) as covariates. For education associations, tertiary level was the reference group.
