# Supplementary material for: Socioeconomic inequalities in functional ageing trajectories in England and Canada: A comparative longitudinal cohort study
Source: PLoS Med. 2026 Aug 13;23(8):e1004833. doi: 10.1371/journal.pmed.1004833 (PMC13472442; doi:10.1371/journal.pmed.1004833)
Supplement: Supplementary file 9 [file pmed.1004833.s009.docx]

**S8 Table. Associations between wealth and the level of and change in functional health outcomes when adjusting for disease categories**

|  | **ELSA** | | | **CLSA** | | |
| --- | --- | --- | --- | --- | --- | --- |
|  | b | 95% CI | p | b | 95% CI | p |
| **Gait speed, m/s^1^** |  |  |  |  |  |  |
| Age | 0.009 | [-0.012,0.031] | 0.394 | -0.005 | [-0.019,0.008] | 0.464 |
| Age^2^ | 0.000 | [0.000,0.001] | <0.001 | 0.000 | [0.000,0.000] | <0.001 |
| Ref: Highest wealth |  |  |  |  |  |  |
| Higher wealth | 0.073 | [0.049,0.097] | <0.001 | 0.010 | [-0.003,0.023] | 0.142 |
| Lower wealth | 0.134 | [0.102,0.165] | <0.001 | 0.036 | [0.019,0.053] | <0.001 |
| Lowest wealth | 0.213 | [0.182,0.245] | <0.001 | 0.059 | [0.044,0.075] | <0.001 |
| Ref: Highest wealth x Age |  |  |  |  |  |  |
| Higher wealth x Age | -0.001 | [-0.003,0.002] | 0.555 | 0.000 | [-0.002,0.001] | 0.583 |
| Lower wealth x Age | -0.002 | [-0.005,0.001] | 0.311 | -0.001 | [-0.003,0.001] | 0.316 |
| Lowest wealth x Age | -0.003 | [-0.006,0.000] | 0.078 | -0.001 | [-0.003,0.001] | 0.348 |
| **Grip strength, kg^1^** |  |  |  |  |  |  |
| Age | -0.240 | [-0.729,0.250] | 0.337 | -0.483 | [-0.818,-0.147] | 0.005 |
| Age^2^ | 0.008 | [0.006,0.010] | <0.001 | 0.008 | [0.007,0.009] | <0.001 |
| Ref: Highest wealth |  |  |  |  |  |  |
| Higher wealth | 0.520 | [-0.012,1.052] | 0.055 | 0.401 | [0.057,0.745] | 0.022 |
| Lower wealth | 1.872 | [1.181,2.562] | <0.001 | 0.826 | [0.408,1.245] | <0.001 |
| Lowest wealth | 2.472 | [1.787,3.156] | <0.001 | 1.436 | [1.026,1.845] | <0.001 |
| Ref: Highest wealth x Age |  |  |  |  |  |  |
| Higher wealth x Age | 0.050 | [-0.003,0.103] | 0.062 | -0.001 | [-0.042,0.039] | 0.946 |
| Lower wealth x Age | 0.027 | [-0.039,0.093] | 0.423 | 0.031 | [-0.015,0.078] | 0.190 |
| Lowest wealth x Age | 0.002 | [-0.064,0.068] | 0.949 | 0.030 | [-0.017,0.077] | 0.207 |
| **Chair rise time, log s** |  |  |  |  |  |  |
| Age | 0.009 | [0.004,0.015] | 0.001 | -0.001 | [-0.003,0.001] | 0.34 |
| Age^2^ | 0.000 | [0.000,0.000] | <0.001 | 0.000 | [0.000,0.000] | <0.001 |
| Ref: Highest wealth |  |  |  |  |  |  |
| Higher wealth | 0.048 | [0.025,0.071] | <0.001 | 0.030 | [0.019,0.041] | <0.001 |
| Lower wealth | 0.114 | [0.084,0.143] | <0.001 | 0.047 | [0.034,0.061] | <0.001 |
| Lowest wealth | 0.160 | [0.128,0.191] | <0.001 | 0.086 | [0.073,0.100] | <0.001 |
| Ref: Highest wealth x Age |  |  |  |  |  |  |
| Higher wealth x Age | -0.001 | [-0.004,0.002] | 0.559 | -0.002 | [-0.004,-0.001] | 0.005 |
| Lower wealth x Age | -0.001 | [-0.005,0.003] | 0.563 | -0.003 | [-0.005,-0.002] | <0.001 |
| Lowest wealth x Age | -0.004 | [-0.008,-0.000] | 0.046 | -0.003 | [-0.005,-0.001] | 0.001 |
| **FEV, litres^1^** |  |  |  |  |  |  |
| Age | 0.033 | [0.023,0.042] | <0.001 | 0.036 | [0.033,0.039] | <0.001 |
| Age^2^ | 0.000 | [0.000,0.001] | <0.001 | 0.000 | [0.000,0.000] | <0.001 |
| Ref: Highest wealth |  |  |  |  |  |  |
| Higher wealth | 0.153 | [0.103,0.203] | <0.001 | 0.059 | [0.032,0.086] | <0.001 |
| Lower wealth | 0.296 | [0.230,0.362] | <0.001 | 0.145 | [0.112,0.177] | <0.001 |
| Lowest wealth | 0.376 | [0.311,0.442] | <0.001 | 0.224 | [0.192,0.255] | <0.001 |
| Ref: Highest wealth x Age |  |  |  |  |  |  |
| Higher wealth x Age | 0.001 | [-0.005,0.007] | 0.746 | -0.001 | [-0.003,0.002] | 0.551 |
| Lower wealth x Age | -0.002 | [-0.010,0.005] | 0.545 | -0.001 | [-0.004,0.002] | 0.410 |
| Lowest wealth x Age | -0.003 | [-0.010,0.004] | 0.414 | 0.000 | [-0.003,0.003] | 0.995 |
| **Self-rated eyesight** |  |  |  |  |  |  |
| Age | 0.008 | [-0.003,0.018] | 0.143 | 0.020 | [0.013,0.026] | <0.001 |
| Age^2^ | 0.001 | [0.001,0.001] | <0.001 | 0.000 | [-0.000,0.000] | 0.156 |
| Ref: Highest wealth |  |  |  |  |  |  |
| Higher wealth | 0.074 | [0.012,0.136] | 0.020 | 0.065 | [0.031,0.099] | <0.001 |
| Lower wealth | 0.319 | [0.241,0.397] | <0.001 | 0.124 | [0.082,0.166] | <0.001 |
| Lowest wealth | 0.440 | [0.364,0.517] | <0.001 | 0.259 | [0.218,0.301] | <0.001 |
| Ref: Highest wealth x Age |  |  |  |  |  |  |
| Higher wealth x Age | 0.002 | [-0.005,0.009] | 0.544 | -0.001 | [-0.005,0.003] | 0.618 |
| Lower wealth x Age | 0.002 | [-0.006,0.010] | 0.619 | -0.004 | [-0.009,0.001] | 0.115 |
| Lowest wealth x Age | 0.002 | [-0.007,0.010] | 0.702 | -0.006 | [-0.011,-0.001] | 0.016 |
| **Self-rated hearing** |  |  |  |  |  |  |
| Age | 0.023 | [0.012,0.034] | <0.001 | 0.039 | [0.032,0.046] | <0.001 |
| Age^2^ | 0.001 | [0.000,0.001] | <0.001 | 0.000 | [0.000,0.000] | 0.727 |
| Ref: Highest wealth |  |  |  |  |  |  |
| Higher wealth | 0.065 | [-0.009,0.139] | 0.086 | 0.047 | [0.007,0.087] | 0.022 |
| Lower wealth | 0.240 | [0.146,0.334] | <0.001 | 0.052 | [0.003,0.101] | 0.038 |
| Lowest wealth | 0.207 | [0.117,0.297] | <0.001 | 0.102 | [0.054,0.150] | <0.001 |
| Ref: Highest wealth x Age |  |  |  |  |  |  |
| Higher wealth x Age | -0.008 | [-0.015,-0.001] | 0.029 | -0.001 | [-0.005,0.004] | 0.824 |
| Lower wealth x Age | -0.008 | [-0.017,0.001] | 0.077 | -0.003 | [-0.009,0.002] | 0.202 |
| Lowest wealth x Age | -0.003 | [-0.011,0.006] | 0.574 | -0.003 | [-0.008,0.003] | 0.359 |
| **ADL difficulties, number square root** |  |  |  |  |  |  |
| Age | 0.005 | [-0.002,0.012] | 0.154 | 0.012 | [0.009,0.015] | <0.001 |
| Age^2^ | 0.001 | [0.001,0.001] | <0.001 | 0.000 | [0.000,0.000] | <0.001 |
| Ref: Highest wealth |  |  |  |  |  |  |
| Higher wealth | 0.048 | [0.022,0.075] | <0.001 | 0.015 | [0.003,0.027] | 0.012 |
| Lower wealth | 0.136 | [0.094,0.177] | <0.001 | 0.040 | [0.025,0.056] | <0.001 |
| Lowest wealth | 0.259 | [0.216,0.301] | <0.001 | 0.093 | [0.075,0.110] | <0.001 |
| Ref: Highest wealth x Age |  |  |  |  |  |  |
| Higher wealth x Age | 0.003 | [-0.001,0.007] | 0.125 | 0.000 | [-0.002,0.002] | 0.947 |
| Lower wealth x Age | 0.010 | [0.005,0.015] | <0.001 | 0.002 | [0.000,0.004] | 0.068 |
| Lowest wealth x Age | 0.009 | [0.004,0.014] | <0.001 | 0.002 | [0.000,0.005] | 0.027 |
| **IADL difficulties, number square root** |  |  |  |  |  |  |
| Age | 0.006 | [-0.001,0.013] | 0.099 | 0.012 | [0.008,0.015] | <0.001 |
| Age^2^ | 0.002 | [0.002,0.002] | <0.001 | 0.001 | [0.001,0.001] | <0.001 |
| Ref: Highest wealth |  |  |  |  |  |  |
| Higher wealth | 0.036 | [0.006,0.067] | 0.018 | -0.003 | [-0.011,0.006] | 0.541 |
| Lower wealth | 0.117 | [0.072,0.163] | <0.001 | 0.000 | [-0.013,0.012] | 0.949 |
| Lowest wealth | 0.261 | [0.214,0.309] | <0.001 | 0.043 | [0.029,0.057] | <0.001 |
| Ref: Highest wealth x Age |  |  |  |  |  |  |
| Higher wealth x Age | 0.005 | [0.001,0.009] | 0.027 | 0.001 | [-0.001,0.002] | 0.265 |
| Lower wealth x Age | 0.013 | [0.008,0.018] | <0.001 | 0.002 | [0.001,0.004] | 0.004 |
| Lowest wealth x Age | 0.015 | [0.010,0.021] | <0.001 | 0.003 | [0.001,0.005] | 0.001 |
| **Mobility difficulties, number** |  |  |  |  |  |  |
| Age | 0.008 | [-0.016,0.032] | 0.512 | - | - | - |
| Age^2^ | 0.004 | [0.004,0.005] | <0.001 | - | - | - |
| Ref: Highest wealth |  |  |  |  |  |  |
| Higher wealth | 0.292 | [0.155,0.429] | <0.001 | - | - | - |
| Lower wealth | 0.956 | [0.746,1.167] | <0.001 | - | - | - |
| Lowest wealth | 1.827 | [1.620,2.033] | <0.001 | - | - | - |
| Ref: Highest wealth x Age |  |  |  |  |  |  |
| Higher wealth x Age | 0.018 | [0.003,0.033] | 0.019 |  |  |  |
| Lower wealth x Age | 0.029 | [0.009,0.049] | 0.004 | - | - | - |
| Lowest wealth x Age | 0.032 | [0.012,0.053] | 0.002 | - | - | - |
| **Up and go, log s** |  |  |  |  |  |  |
| Age | - | - | - | 0.015 | [0.014,0.017] | <0.001 |
| Age^2^ | - | - | - | 0.000 | [0.000,0.000] | <0.001 |
| Ref: Highest wealth |  |  |  |  |  |  |
| Higher wealth | - | - | - | 0.007 | [-0.002,0.015] | 0.129 |
| Lower wealth | - | - | - | 0.027 | [0.016,0.039] | <0.001 |
| Lowest wealth | - | - | - | 0.066 | [0.055,0.077] | <0.001 |
| Ref: Highest wealth x Age |  |  |  |  |  |  |
| Higher wealth x Age |  |  |  | 0.001 | [0.000,0.002] | 0.033 |
| Lower wealth x Age | - | - | - | 0.001 | [-0.001,0.002] | 0.250 |
| Lowest wealth x Age | - | - | - | 0.002 | [0.001,0.003] | 0.003 |

ELSA=English Longitudinal Study of Ageing; CLSA=Canadian Longitudinal Study on Aging; FEV=Forced Expiratory Volume; ADL=basic activities of daily living; IADL=instrumental activities of daily living. ^1^For measures where higher values originally indicated better function (gait speed, grip strength, and lung function), we reversed the direction by multiplying by −1. This transformation does not affect model fit or statistical inference but ensures consistent interpretation across outcomes. Models included age^2^, birth year, sex, race, height (gait speed and grip strength analyses only), smoking, alcohol consumption, weight status, and chronic conditions (respiratory, muscoskeletal, neurological, mental, cardiovascular, diabetes, and cancer) as covariates. For wealth associations, the highest category was the reference group.
