# Supplementary material for: Socioeconomic inequalities in functional ageing trajectories in England and Canada: A comparative longitudinal cohort study
Source: PLoS Med. 2026 Aug 13;23(8):e1004833. doi: 10.1371/journal.pmed.1004833 (PMC13472442; doi:10.1371/journal.pmed.1004833)
Supplement: Supplementary file 10 [file pmed.1004833.s010.docx]

**S9 Table. Associations between wealth and the level of and change in functional health outcomes in the pooled analysis**

|  | **b** | **95% CI** | **p** |
| --- | --- | --- | --- |
| **Gait speed, m/s** |  |  |  |
| Age | -0.006 | [-0.021,0.009] | 0.444 |
| Age^2^ | -0.000 | [-0.000,-0.000] | <0.001 |
| Ref: Highest wealth |  |  |  |
| Higher wealth | -0.077 | [-0.096,-0.059] | <0.001 |
| Lower wealth | -0.156 | [-0.179,-0.133] | <0.001 |
| Lowest wealth | -0.266 | [-0.288,-0.244] | <0.001 |
| Ref: Highest wealth x cohort=ELSA |  |  |  |
| Ref: Highest wealth x cohort=CLSA |  |  |  |
| Higher wealth x cohort=ELSA |  |  |  |
| Higher wealth x cohort=CLSA | 0.060 | [0.038,0.081] | <0.001 |
| Lower wealth x cohort=ELSA |  |  |  |
| Lower wealth x cohort=CLSA | 0.110 | [0.083,0.137] | <0.001 |
| Lowest wealth x cohort=ELSA |  |  |  |
| Lowest wealth x cohort=CLSA | 0.191 | [0.166,0.217] | <0.001 |
| Ref: Highest wealth x Age |  |  |  |
| Higher wealth x Age | 0.001 | [-0.001,0.003] | 0.351 |
| Lower wealth x Age | 0.002 | [0.000,0.004] | 0.040 |
| Lowest wealth x Age | 0.005 | [0.003,0.008] | <0.001 |
| Ref: Highest wealth x cohort=ELSA x Age |  |  |  |
| Ref: Highest wealth x cohort=CLSA x Age |  |  |  |
| Higher wealth x cohort=ELSA x Age |  |  |  |
| Higher wealth x cohort=CLSA x Age | -0.000 | [-0.002,0.002] | 0.872 |
| Lower wealth x cohort=ELSA x Age |  |  |  |
| Lower wealth x cohort=CLSA x Age | -0.001 | [-0.004,0.001] | 0.340 |
| Lowest wealth x cohort=ELSA x Age |  |  |  |
| Lowest wealth x cohort=CLSA x Age | -0.004 | [-0.007,-0.002] | 0.001 |
| Observations | 48,613 |  |  |
| **Grip strength, kg** |  |  |  |
| Age | -0.088 | [-0.490,0.314] | 0.669 |
| Age^2^ | -0.005 | [-0.006,-0.005] | <0.001 |
| Ref: Highest wealth |  |  |  |
| Higher wealth | -0.643 | [-1.115,-0.170] | 0.008 |
| Lower wealth | -1.814 | [-2.392,-1.237] | <0.001 |
| Lowest wealth | -2.838 | [-3.388,-2.288] | <0.001 |
| Ref: Highest wealth x cohort=ELSA |  |  |  |
| Ref: Highest wealth x cohort=CLSA |  |  |  |
| Higher wealth x cohort=ELSA |  |  |  |
| Higher wealth x cohort=CLSA | 0.230 | [-0.311,0.771] | 0.404 |
| Lower wealth x cohort=ELSA |  |  |  |
| Lower wealth x cohort=CLSA | 0.971 | [0.312,1.630] | 0.004 |
| Lowest wealth x cohort=ELSA |  |  |  |
| Lowest wealth x cohort=CLSA | 1.310 | [0.683,1.936] | <0.001 |
| Ref: Highest wealth x Age |  |  |  |
| Higher wealth x Age | 0.020 | [-0.031,0.072] | 0.439 |
| Lower wealth x Age | 0.049 | [-0.012,0.109] | 0.116 |
| Lowest wealth x Age | 0.069 | [0.012,0.126] | 0.017 |
| Ref: Highest wealth x cohort=ELSA x Age |  |  |  |
| Ref: Highest wealth x cohort=CLSA x Age |  |  |  |
| Higher wealth x cohort=ELSA x Age |  |  |  |
| Higher wealth x cohort=CLSA x Age | -0.005 | [-0.062,0.052] | 0.866 |
| Lower wealth x cohort=ELSA x Age |  |  |  |
| Lower wealth x cohort=CLSA x Age | -0.043 | [-0.111,0.024] | 0.209 |
| Lowest wealth x cohort=ELSA x Age |  |  |  |
| Lowest wealth x cohort=CLSA x Age | -0.049 | [-0.112,0.015] | 0.130 |
| Observations | 63,722 |  |  |
| **Chair rise time, log s** |  |  |  |
| Age | 0.012 | [0.010,0.014] | <0.001 |
| Age^2^ | 0.000 | [0.000,0.000] | <0.001 |
| Ref: Highest wealth |  |  |  |
| Higher wealth | 0.065 | [0.047,0.082] | <0.001 |
| Lower wealth | 0.135 | [0.113,0.157] | <0.001 |
| Lowest wealth | 0.187 | [0.165,0.208] | <0.001 |
| Ref: Highest wealth x cohort=ELSA |  |  |  |
| Ref: Highest wealth x cohort=CLSA |  |  |  |
| Higher wealth x cohort=ELSA |  |  |  |
| Higher wealth x cohort=CLSA | -0.027 | [-0.047,-0.007] | 0.008 |
| Lower wealth x cohort=ELSA |  |  |  |
| Lower wealth x cohort=CLSA | -0.073 | [-0.098,-0.048] | <0.001 |
| Lowest wealth x cohort=ELSA |  |  |  |
| Lowest wealth x cohort=CLSA | -0.077 | [-0.101,-0.053] | <0.001 |
| Ref: Highest wealth x Age |  |  |  |
| Higher wealth x Age | -0.001 | [-0.003,0.001] | 0.488 |
| Lower wealth x Age | -0.001 | [-0.003,0.002] | 0.637 |
| Lowest wealth x Age | -0.003 | [-0.006,-0.001] | 0.006 |
| Ref: Highest wealth x cohort=ELSA x Age |  |  |  |
| Ref: Highest wealth x cohort=CLSA x Age |  |  |  |
| Higher wealth x cohort=ELSA x Age |  |  |  |
| Higher wealth x cohort=CLSA x Age | -0.001 | [-0.003,0.001] | 0.493 |
| Lower wealth x cohort=ELSA x Age |  |  |  |
| Lower wealth x cohort=CLSA x Age | -0.002 | [-0.004,0.001] | 0.231 |
| Lowest wealth x cohort=ELSA x Age |  |  |  |
| Lowest wealth x cohort=CLSA x Age | 0.001 | [-0.001,0.004] | 0.328 |
| Observations | 65,319 |  |  |
| **FEV, l** |  |  |  |
| Age | -0.037 | [-0.040,-0.034] | <0.001 |
| Age^2^ | -0.000 | [-0.000,-0.000] | <0.001 |
| Ref: Highest wealth |  |  |  |
| Higher wealth | -0.150 | [-0.188,-0.111] | <0.001 |
| Lower wealth | -0.311 | [-0.358,-0.263] | <0.001 |
| Lowest wealth | -0.445 | [-0.490,-0.399] | <0.001 |
| Ref: Highest wealth x cohort=ELSA |  |  |  |
| Ref: Highest wealth x cohort=CLSA |  |  |  |
| Higher wealth x cohort=ELSA |  |  |  |
| Higher wealth x cohort=CLSA | 0.074 | [0.029,0.119] | 0.001 |
| Lower wealth x cohort=ELSA |  |  |  |
| Lower wealth x cohort=CLSA | 0.138 | [0.083,0.193] | <0.001 |
| Lowest wealth x cohort=ELSA |  |  |  |
| Lowest wealth x cohort=CLSA | 0.182 | [0.130,0.235] | <0.001 |
| Ref: Highest wealth x Age |  |  |  |
| Higher wealth x Age | -0.003 | [-0.006,0.001] | 0.119 |
| Lower wealth x Age | -0.001 | [-0.005,0.003] | 0.650 |
| Lowest wealth x Age | 0.000 | [-0.004,0.004] | 0.916 |
| Ref: Highest wealth x cohort=ELSA x Age |  |  |  |
| Ref: Highest wealth x cohort=CLSA x Age |  |  |  |
| Higher wealth x cohort=ELSA x Age |  |  |  |
| Higher wealth x cohort=CLSA x Age | 0.004 | [0.000,0.008] | 0.034 |
| Lower wealth x cohort=ELSA x Age |  |  |  |
| Lower wealth x cohort=CLSA x Age | 0.004 | [-0.001,0.009] | 0.115 |
| Lowest wealth x cohort=ELSA x Age |  |  |  |
| Lowest wealth x cohort=CLSA x Age | 0.003 | [-0.002,0.007] | 0.265 |
| Observations | 57,586 |  |  |
| **Self-rated eyesight** |  |  |  |
| Age | 0.017 | [0.012,0.023] | <0.001 |
| Age^2^ | 0.000 | [0.000,0.000] | <0.001 |
| Ref: Highest wealth |  |  |  |
| Higher wealth | 0.100 | [0.046,0.154] | <0.001 |
| Lower wealth | 0.341 | [0.276,0.406] | <0.001 |
| Lowest wealth | 0.534 | [0.474,0.594] | <0.001 |
| Ref: Highest wealth x cohort=ELSA |  |  |  |
| Ref: Highest wealth x cohort=CLSA |  |  |  |
| Higher wealth x cohort=ELSA |  |  |  |
| Higher wealth x cohort=CLSA | -0.023 | [-0.085,0.039] | 0.460 |
| Lower wealth x cohort=ELSA |  |  |  |
| Lower wealth x cohort=CLSA | -0.191 | [-0.265,-0.116] | <0.001 |
| Lowest wealth x cohort=ELSA |  |  |  |
| Lowest wealth x cohort=CLSA | -0.242 | [-0.311,-0.174] | <0.001 |
| Ref: Highest wealth x Age |  |  |  |
| Higher wealth x Age | 0.001 | [-0.004,0.007] | 0.646 |
| Lower wealth x Age | 0.002 | [-0.004,0.009] | 0.519 |
| Lowest wealth x Age | -0.005 | [-0.012,0.001] | 0.078 |
| Ref: Highest wealth x cohort=ELSA x Age |  |  |  |
| Ref: Highest wealth x cohort=CLSA x Age |  |  |  |
| Higher wealth x cohort=ELSA x Age |  |  |  |
| Higher wealth x cohort=CLSA x Age | -0.004 | [-0.011,0.002] | 0.172 |
| Lower wealth x cohort=ELSA x Age |  |  |  |
| Lower wealth x cohort=CLSA x Age | -0.007 | [-0.014,0.001] | 0.080 |
| Lowest wealth x cohort=ELSA x Age |  |  |  |
| Lowest wealth x cohort=CLSA x Age | -0.002 | [-0.009,0.005] | 0.574 |
| Observations | 88,178 |  |  |
| **Self-rated hearing** |  |  |  |
| Age | 0.032 | [0.027,0.038] | <0.001 |
| Age^2^ | 0.000 | [0.000,0.000] | 0.003 |
| Ref: Highest wealth |  |  |  |
| Higher wealth | 0.066 | [0.005,0.128] | 0.035 |
| Lower wealth | 0.243 | [0.169,0.317] | <0.001 |
| Lowest wealth | 0.274 | [0.205,0.342] | <0.001 |
| Ref: Highest wealth x cohort=ELSA |  |  |  |
| Ref: Highest wealth x cohort=CLSA |  |  |  |
| Higher wealth x cohort=ELSA |  |  |  |
| Higher wealth x cohort=CLSA | -0.018 | [-0.089,0.053] | 0.620 |
| Lower wealth x cohort=ELSA |  |  |  |
| Lower wealth x cohort=CLSA | -0.166 | [-0.251,-0.080] | <0.001 |
| Lowest wealth x cohort=ELSA |  |  |  |
| Lowest wealth x cohort=CLSA | -0.134 | [-0.213,-0.055] | 0.001 |
| Ref: Highest wealth x Age |  |  |  |
| Higher wealth x Age | -0.007 | [-0.012,-0.001] | 0.021 |
| Lower wealth x Age | -0.006 | [-0.013,0.000] | 0.062 |
| Lowest wealth x Age | -0.006 | [-0.013,0.000] | 0.055 |
| Ref: Highest wealth x cohort=ELSA x Age |  |  |  |
| Ref: Highest wealth x cohort=CLSA x Age |  |  |  |
| Higher wealth x cohort=ELSA x Age |  |  |  |
| Higher wealth x cohort=CLSA x Age | 0.006 | [-0.001,0.013] | 0.087 |
| Lower wealth x cohort=ELSA x Age |  |  |  |
| Lower wealth x cohort=CLSA x Age | 0.002 | [-0.006,0.010] | 0.607 |
| Lowest wealth x cohort=ELSA x Age |  |  |  |
| Lowest wealth x cohort=CLSA x Age | 0.001 | [-0.006,0.009] | 0.746 |
| Observations | 88,173 |  |  |
| **ADL difficulties, number square root** |  |  |  |
| Age | 0.008 | [0.005,0.010] | <0.001 |
| Age^2^ | 0.000 | [0.000,0.000] | <0.001 |
| Ref: Highest wealth |  |  |  |
| Higher wealth | 0.057 | [0.032,0.082] | <0.001 |
| Lower wealth | 0.178 | [0.148,0.208] | <0.001 |
| Lowest wealth | 0.333 | [0.305,0.361] | <0.001 |
| Ref: Highest wealth x cohort=ELSA |  |  |  |
| Ref: Highest wealth x cohort=CLSA |  |  |  |
| Higher wealth x cohort=ELSA |  |  |  |
| Higher wealth x cohort=CLSA | -0.033 | [-0.061,-0.004] | 0.027 |
| Lower wealth x cohort=ELSA |  |  |  |
| Lower wealth x cohort=CLSA | -0.122 | [-0.156,-0.087] | <0.001 |
| Lowest wealth x cohort=ELSA |  |  |  |
| Lowest wealth x cohort=CLSA | -0.213 | [-0.245,-0.180] | <0.001 |
| Ref: Highest wealth x Age |  |  |  |
| Higher wealth x Age | 0.003 | [0.000,0.006] | 0.033 |
| Lower wealth x Age | 0.007 | [0.004,0.010] | <0.001 |
| Lowest wealth x Age | 0.004 | [0.001,0.007] | 0.011 |
| Ref: Highest wealth x cohort=ELSA x Age |  |  |  |
| Ref: Highest wealth x cohort=CLSA x Age |  |  |  |
| Higher wealth x cohort=ELSA x Age |  |  |  |
| Higher wealth x cohort=CLSA x Age | -0.004 | [-0.007,-0.001] | 0.021 |
| Lower wealth x cohort=ELSA x Age |  |  |  |
| Lower wealth x cohort=CLSA x Age | -0.007 | [-0.011,-0.003] | <0.001 |
| Lowest wealth x cohort=ELSA x Age |  |  |  |
| Lowest wealth x cohort=CLSA x Age | -0.004 | [-0.007,-0.000] | 0.045 |
| Observations | 89,010 |  |  |
| **IADL difficulties, number square root** |  |  |  |
| Age | 0.007 | [0.005,0.010] | <0.001 |
| Age^2^ | 0.001 | [0.001,0.001] | <0.001 |
| Ref: Highest wealth |  |  |  |
| Higher wealth | 0.053 | [0.032,0.074] | <0.001 |
| Lower wealth | 0.169 | [0.144,0.194] | <0.001 |
| Lowest wealth | 0.363 | [0.339,0.387] | <0.001 |
| Ref: Highest wealth x cohort=ELSA |  |  |  |
| Ref: Highest wealth x cohort=CLSA |  |  |  |
| Higher wealth x cohort=ELSA |  |  |  |
| Higher wealth x cohort=CLSA | -0.048 | [-0.072,-0.023] | <0.001 |
| Lower wealth x cohort=ELSA |  |  |  |
| Lower wealth x cohort=CLSA | -0.152 | [-0.182,-0.123] | <0.001 |
| Lowest wealth x cohort=ELSA |  |  |  |
| Lowest wealth x cohort=CLSA | -0.299 | [-0.326,-0.271] | <0.001 |
| Ref: Highest wealth x Age |  |  |  |
| Higher wealth x Age | 0.004 | [0.001,0.006] | 0.002 |
| Lower wealth x Age | 0.009 | [0.006,0.012] | <0.001 |
| Lowest wealth x Age | 0.007 | [0.004,0.009] | <0.001 |
| Ref: Highest wealth x cohort=ELSA x Age |  |  |  |
| Ref: Highest wealth x cohort=CLSA x Age |  |  |  |
| Higher wealth x cohort=ELSA x Age |  |  |  |
| Higher wealth x cohort=CLSA x Age | -0.003 | [-0.006,-0.001] | 0.019 |
| Lower wealth x cohort=ELSA x Age |  |  |  |
| Lower wealth x cohort=CLSA x Age | -0.007 | [-0.011,-0.004] | <0.001 |
| Lowest wealth x cohort=ELSA x Age |  |  |  |
| Lowest wealth x cohort=CLSA x Age | -0.004 | [-0.007,-0.001] | 0.016 |
| Observations | 89,010 |  |  |

ELSA=English Longitudinal Study of Ageing; CLSA=Canadian Longitudinal Study on Aging; FEV=Forced Expiratory Volume; ADL=basic activities of daily living; IADL=instrumental activities of daily living. Model included age^2^, birth year, sex, race, and height (gait speed and grip strength analyses only). For wealth associations, the highest category was the reference group.
