# Supplementary material for: Socioeconomic inequalities in functional ageing trajectories in England and Canada: A comparative longitudinal cohort study
Source: PLoS Med. 2026 Aug 13;23(8):e1004833. doi: 10.1371/journal.pmed.1004833 (PMC13472442; doi:10.1371/journal.pmed.1004833)
Supplement: Supplementary file 11 [file pmed.1004833.s011.docx]

**S10 Table. Effects of sex and race on the association between wealth and functional health outcomes in the two cohorts**

|  | **ELSA** | | | **CLSA** | | |
| --- | --- | --- | --- | --- | --- | --- |
|  | b | 95% CI | p | b | 95% CI | p |
| **Gait speed, m/s^1^** |  |  |  |  |  |  |
| Age | 0.012 | [-0.011,0.034] | 0.311 | -0.005 | [-0.019,0.009] | 0.494 |
| Age^2^ | 0.001 | [0.000,0.001] | <0.001 | 0.000 | [0.000,0.000] | <0.001 |
| Ref: Highest wealth |  |  |  |  |  |  |
| Higher wealth | 0.087 | [0.049,0.126] | <0.001 | 0.012 | [-0.006,0.029] | 0.197 |
| Lower wealth | 0.204 | [0.154,0.254] | <0.001 | 0.041 | [0.016,0.066] | 0.001 |
| Lowest wealth | 0.279 | [0.230,0.329] | <0.001 | 0.068 | [0.046,0.090] | <0.001 |
| Ref: White |  |  |  |  |  |  |
| Non-white | 0.273 | [0.168,0.378] | <0.001 | 0.014 | [-0.061,0.089] | 0.711 |
| Highest wealth x White |  |  |  |  |  |  |
| Highest wealth x Non-white |  |  |  |  |  |  |
| Higher wealth x White |  |  |  |  |  |  |
| Higher wealth x Non-white | -0.064 | [-0.193,0.066] | 0.335 | 0.019 | [-0.070,0.108] | 0.676 |
| Lower wealth x White |  |  |  |  |  |  |
| Lower wealth x Non-white | -0.304 | [-0.487,-0.121] | 0.001 | 0.044 | [-0.066,0.154] | 0.434 |
| Lowest wealth x White |  |  |  |  |  |  |
| Lowest wealth x Non-white | -0.067 | [-0.212,0.078] | 0.366 | 0.033 | [-0.059,0.124] | 0.484 |
| Ref: Highest wealth x Age |  |  |  |  |  |  |
| Higher wealth x Age | -0.002 | [-0.006,0.002] | 0.441 | -0.000 | [-0.002,0.002] | 0.780 |
| Lower wealth x Age | -0.004 | [-0.009,0.001] | 0.095 | -0.002 | [-0.005,0.001] | 0.126 |
| Lowest wealth x Age | -0.005 | [-0.010,-0.000] | 0.044 | -0.001 | [-0.003,0.002] | 0.662 |
| Ref: White x Age |  |  |  |  |  |  |
| Non-white x Age | -0.014 | [-0.032,0.004] | 0.122 | -0.002 | [-0.008,0.005] | 0.643 |
| Highest wealth x White x Age |  |  |  |  |  |  |
| Highest wealth x Non-white x Age |  |  |  |  |  |  |
| Higher wealth x White x Age |  |  |  |  |  |  |
| Higher wealth x Non-white x Age | 0.004 | [-0.015,0.023] | 0.680 | 0.006 | [-0.002,0.015] | 0.131 |
| Lower wealth x White x Age |  |  |  |  |  |  |
| Lower wealth x Non-white x Age | 0.017 | [-0.006,0.040] | 0.144 | 0.002 | [-0.007,0.011] | 0.647 |
| Lowest wealth x White x Age |  |  |  |  |  |  |
| Lowest wealth x Non-white x Age | 0.011 | [-0.009,0.031] | 0.276 | -0.000 | [-0.010,0.009] | 0.919 |
| Ref: Male |  |  |  |  |  |  |
| Female | 0.003 | [-0.044,0.050] | 0.900 | -0.017 | [-0.043,0.009] | 0.199 |
| Highest wealth x Male |  |  |  |  |  |  |
| Highest wealth x Female |  |  |  |  |  |  |
| Higher wealth x Male |  |  |  |  |  |  |
| Higher wealth x Female | 0.001 | [-0.048,0.051] | 0.962 | 0.013 | [-0.014,0.039] | 0.359 |
| Lower wealth x Male |  |  |  |  |  |  |
| Lower wealth x Female | -0.052 | [-0.116,0.011] | 0.105 | 0.014 | [-0.021,0.049] | 0.437 |
| Lowest wealth x Male |  |  |  |  |  |  |
| Lowest wealth x Female | -0.026 | [-0.088,0.037] | 0.423 | 0.024 | [-0.008,0.055] | 0.140 |
| Ref: Male x Age |  |  |  |  |  |  |
| Female x Age | -0.001 | [-0.006,0.004] | 0.791 | 0.002 | [-0.002,0.005] | 0.311 |
| Highest wealth x Male x Age |  |  |  |  |  |  |
| Highest wealth x Female x Age |  |  |  |  |  |  |
| Higher wealth x Male x Age |  |  |  |  |  |  |
| Higher wealth x Female x Age | 0.001 | [-0.004,0.006] | 0.713 | -0.001 | [-0.004,0.002] | 0.527 |
| Lower wealth x Male x Age |  |  |  |  |  |  |
| Lower wealth x Female x Age | 0.002 | [-0.004,0.009] | 0.436 | 0.001 | [-0.003,0.005] | 0.526 |
| Lowest wealth x Male x Age |  |  |  |  |  |  |
| Lowest wealth x Female x Age | 0.002 | [-0.004,0.009] | 0.474 | -0.001 | [-0.005,0.003] | 0.525 |
| **Grip strength, kg^1^** |  |  |  |  |  |  |
| Age | -0.171 | [-0.672,0.329] | 0.502 | -0.447 | [-0.785,-0.109] | 0.009 |
| Age^2^ | 0.008 | [0.007,0.010] | <0.001 | 0.008 | [0.007,0.009] | <0.001 |
| Ref: Highest wealth |  |  |  |  |  |  |
| Higher wealth | 0.141 | [-0.766,1.047] | 0.761 | 0.623 | [0.115,1.131] | 0.016 |
| Lower wealth | 2.078 | [0.894,3.263] | 0.001 | 1.474 | [0.809,2.139] | <0.001 |
| Lowest wealth | 2.853 | [1.722,3.985] | <0.001 | 2.582 | [1.938,3.225] | <0.001 |
| Ref: White |  |  |  |  |  |  |
| Non-white | 0.257 | [-3.210,3.723] | 0.885 | 3.630 | [1.774,5.487] | <0.001 |
| Highest wealth x White |  |  |  |  |  |  |
| Highest wealth x Non-white |  |  |  |  |  |  |
| Higher wealth x White |  |  |  |  |  |  |
| Higher wealth x Non-white | 2.706 | [-1.726,7.139] | 0.231 | -0.697 | [-2.906,1.511] | 0.536 |
| Lower wealth x White |  |  |  |  |  |  |
| Lower wealth x Non-white | 1.501 | [-3.039,6.040] | 0.517 | -0.922 | [-3.407,1.564] | 0.467 |
| Lowest wealth x White |  |  |  |  |  |  |
| Lowest wealth x Non-white | -0.729 | [-4.833,3.375] | 0.728 | -2.894 | [-5.125,-0.663] | 0.011 |
| Ref: Highest wealth x Age |  |  |  |  |  |  |
| Higher wealth x Age | 0.064 | [-0.026,0.154] | 0.161 | -0.005 | [-0.063,0.053] | 0.867 |
| Lower wealth x Age | 0.069 | [-0.043,0.180] | 0.230 | 0.044 | [-0.026,0.114] | 0.219 |
| Lowest wealth x Age | 0.026 | [-0.086,0.138] | 0.652 | 0.028 | [-0.043,0.099] | 0.440 |
| Ref: White x Age |  |  |  |  |  |  |
| Non-white x Age | 0.062 | [-0.247,0.371] | 0.693 | -0.083 | [-0.297,0.132] | 0.451 |
| Highest wealth x White x Age |  |  |  |  |  |  |
| Highest wealth x Non-white x Age |  |  |  |  |  |  |
| Higher wealth x White x Age |  |  |  |  |  |  |
| Higher wealth x Non-white x Age | -0.235 | [-0.614,0.144] | 0.224 | 0.124 | [-0.127,0.376] | 0.333 |
| Lower wealth x White x Age |  |  |  |  |  |  |
| Lower wealth x Non-white x Age | 0.209 | [-0.185,0.602] | 0.298 | 0.035 | [-0.235,0.304] | 0.800 |
| Lowest wealth x White x Age |  |  |  |  |  |  |
| Lowest wealth x Non-white x Age | -0.205 | [-0.575,0.165] | 0.277 | -0.009 | [-0.259,0.241] | 0.942 |
| Ref: Male |  |  |  |  |  |  |
| Female | 12.673 | [11.628,13.719] | <0.001 | 13.445 | [12.830,14.060] | <0.001 |
| Highest wealth x Male |  |  |  |  |  |  |
| Highest wealth x Female |  |  |  |  |  |  |
| Higher wealth x Male |  |  |  |  |  |  |
| Higher wealth x Female | 0.580 | [-0.486,1.647] | 0.286 | -0.636 | [-1.278,0.006] | 0.052 |
| Lower wealth x Male |  |  |  |  |  |  |
| Lower wealth x Female | -0.404 | [-1.780,0.972] | 0.565 | -1.461 | [-2.268,-0.653] | <0.001 |
| Lowest wealth x Male |  |  |  |  |  |  |
| Lowest wealth x Female | -0.587 | [-1.907,0.734] | 0.384 | -2.053 | [-2.828,-1.277] | <0.001 |
| Ref: Male x Age |  |  |  |  |  |  |
| Female x Age | -0.196 | [-0.298,-0.093] | <0.001 | -0.150 | [-0.224,-0.076] | <0.001 |
| Highest wealth x Male x Age |  |  |  |  |  |  |
| Highest wealth x Female x Age |  |  |  |  |  |  |
| Higher wealth x Male x Age |  |  |  |  |  |  |
| Higher wealth x Female x Age | -0.015 | [-0.121,0.091] | 0.783 | -0.005 | [-0.081,0.072] | 0.907 |
| Lower wealth x Male x Age |  |  |  |  |  |  |
| Lower wealth x Female x Age | -0.064 | [-0.195,0.066] | 0.335 | -0.030 | [-0.120,0.059] | 0.510 |
| Lowest wealth x Male x Age |  |  |  |  |  |  |
| Lowest wealth x Female x Age | -0.025 | [-0.154,0.103] | 0.700 | 0.006 | [-0.083,0.095] | 0.898 |
| **Chair rise test, log s** |  |  |  |  |  |  |
| Age | 0.007 | [0.003,0.011] | <0.001 | -0.002 | [-0.004,0.000] | 0.101 |
| Age^2^ | 0.000 | [0.000,0.000] | <0.001 | 0.000 | [0.000,0.000] | <0.001 |
| Ref: Highest wealth |  |  |  |  |  |  |
| Higher wealth | 0.051 | [0.018,0.084] | 0.002 | 0.033 | [0.019,0.048] | <0.001 |
| Lower wealth | 0.111 | [0.068,0.154] | <0.001 | 0.049 | [0.030,0.068] | <0.001 |
| Lowest wealth | 0.199 | [0.155,0.243] | <0.001 | 0.102 | [0.084,0.121] | <0.001 |
| Ref: White |  |  |  |  |  |  |
| Non-white | 0.035 | [-0.179,0.250] | 0.747 | 0.021 | [-0.040,0.082] | 0.505 |
| Highest wealth x White |  |  |  |  |  |  |
| Highest wealth x Non-white |  |  |  |  |  |  |
| Higher wealth x White |  |  |  |  |  |  |
| Higher wealth x Non-white | 0.043 | [-0.189,0.276] | 0.716 | 0.009 | [-0.063,0.081] | 0.801 |
| Lower wealth x White |  |  |  |  |  |  |
| Lower wealth x Non-white | 0.094 | [-0.173,0.361] | 0.490 | 0.030 | [-0.056,0.115] | 0.493 |
| Lowest wealth x White |  |  |  |  |  |  |
| Lowest wealth x Non-white | -0.020 | [-0.297,0.257] | 0.890 | -0.008 | [-0.086,0.069] | 0.834 |
| Ref: Highest wealth x Age |  |  |  |  |  |  |
| Higher wealth x Age | -0.001 | [-0.005,0.003] | 0.623 | -0.001 | [-0.003,0.001] | 0.420 |
| Lower wealth x Age | -0.000 | [-0.005,0.005] | 0.938 | -0.003 | [-0.005,-0.001] | 0.013 |
| Lowest wealth x Age | -0.004 | [-0.009,0.002] | 0.161 | -0.002 | [-0.004,0.000] | 0.104 |
| Ref: White x Age |  |  |  |  |  |  |
| Non-white x Age | 0.015 | [-0.009,0.039] | 0.212 | -0.003 | [-0.011,0.005] | 0.431 |
| Highest wealth x White x Age |  |  |  |  |  |  |
| Highest wealth x Non-white x Age |  |  |  |  |  |  |
| Higher wealth x White x Age |  |  |  |  |  |  |
| Higher wealth x Non-white x Age | -0.021 | [-0.049,0.006] | 0.125 | 0.001 | [-0.007,0.010] | 0.770 |
| Lower wealth x White x Age |  |  |  |  |  |  |
| Lower wealth x Non-white x Age | -0.007 | [-0.041,0.027] | 0.691 | 0.003 | [-0.007,0.012] | 0.579 |
| Lowest wealth x White x Age |  |  |  |  |  |  |
| Lowest wealth x Non-white x Age | -0.023 | [-0.053,0.007] | 0.134 | 0.002 | [-0.008,0.011] | 0.754 |
| Ref: Male |  |  |  |  |  |  |
| Female | 0.030 | [-0.010,0.070] | 0.145 | -0.003 | [-0.024,0.017] | 0.746 |
| Highest wealth x Male |  |  |  |  |  |  |
| Highest wealth x Female |  |  |  |  |  |  |
| Higher wealth x Male |  |  |  |  |  |  |
| Higher wealth x Female | 0.010 | [-0.035,0.056] | 0.656 | 0.006 | [-0.017,0.029] | 0.605 |
| Lower wealth x Male |  |  |  |  |  |  |
| Lower wealth x Female | 0.037 | [-0.021,0.096] | 0.213 | 0.019 | [-0.009,0.047] | 0.175 |
| Lowest wealth x Male |  |  |  |  |  |  |
| Lowest wealth x Female | -0.023 | [-0.083,0.037] | 0.449 | 0.012 | [-0.016,0.039] | 0.406 |
| Ref: Male x Age |  |  |  |  |  |  |
| Female x Age | 0.001 | [-0.004,0.007] | 0.686 | 0.003 | [0.000,0.006] | 0.033 |
| Highest wealth x Male x Age |  |  |  |  |  |  |
| Highest wealth x Female x Age |  |  |  |  |  |  |
| Higher wealth x Male x Age |  |  |  |  |  |  |
| Higher wealth x Female x Age | 0.001 | [-0.005,0.007] | 0.715 | -0.004 | [-0.007,-0.001] | 0.017 |
| Lower wealth x Male x Age |  |  |  |  |  |  |
| Lower wealth x Female x Age | -0.001 | [-0.008,0.006] | 0.776 | -0.002 | [-0.006,0.001] | 0.209 |
| Lowest wealth x Male x Age |  |  |  |  |  |  |
| Lowest wealth x Female x Age | 0.001 | [-0.006,0.009] | 0.739 | -0.004 | [-0.007,-0.000] | 0.038 |
| **FEV, litres^1^** |  |  |  |  |  |  |
| Age | 0.033 | [0.024,0.042] | <0.001 | 0.037 | [0.033,0.040] | <0.001 |
| Age^2^ | 0.000 | [0.000,0.001] | <0.001 | 0.000 | [0.000,0.000] | <0.001 |
| Ref: Highest wealth |  |  | <0.001 |  |  | <0.001 |
| Higher wealth | 0.218 | [0.131,0.304] | <0.001 | 0.086 | [0.046,0.127] | <0.001 |
| Lower wealth | 0.453 | [0.339,0.568] | <0.001 | 0.222 | [0.169,0.275] | <0.001 |
| Lowest wealth | 0.559 | [0.452,0.666] | <0.001 | 0.308 | [0.257,0.359] | <0.001 |
| Ref: White |  |  |  |  |  |  |
| Non-white | 0.488 | [0.152,0.825] | 0.004 | 0.407 | [0.273,0.540] | <0.001 |
| Highest wealth x White |  |  |  |  |  |  |
| Highest wealth x Non-white |  |  |  |  |  |  |
| Higher wealth x White |  |  |  |  |  |  |
| Higher wealth x Non-white | -0.098 | [-0.500,0.303] | 0.632 | 0.119 | [-0.032,0.270] | 0.122 |
| Lower wealth x White |  |  |  |  |  |  |
| Lower wealth x Non-white | 0.029 | [-0.347,0.406] | 0.879 | 0.053 | [-0.117,0.223] | 0.541 |
| Lowest wealth x White |  |  |  |  |  |  |
| Lowest wealth x Non-white | -0.069 | [-0.483,0.344] | 0.742 | -0.061 | [-0.229,0.107] | 0.475 |
| Ref: Highest wealth x Age |  |  |  |  |  |  |
| Higher wealth x Age | 0.001 | [-0.009,0.011] | 0.881 | -0.002 | [-0.005,0.002] | 0.389 |
| Lower wealth x Age | -0.001 | [-0.014,0.012] | 0.885 | -0.001 | [-0.006,0.003] | 0.540 |
| Lowest wealth x Age | -0.002 | [-0.013,0.009] | 0.722 | -0.001 | [-0.006,0.003] | 0.619 |
| Ref: White x Age |  |  |  |  |  |  |
| Non-white x Age | -0.012 | [-0.055,0.031] | 0.576 | -0.011 | [-0.021,-0.001] | 0.030 |
| Highest wealth x White x Age |  |  |  |  |  |  |
| Highest wealth x Non-white x Age |  |  |  |  |  |  |
| Higher wealth x White x Age |  |  |  |  |  |  |
| Higher wealth x Non-white x Age | 0.036 | [-0.018,0.090] | 0.192 | 0.006 | [-0.005,0.018] | 0.289 |
| Lower wealth x White x Age |  |  |  |  |  |  |
| Lower wealth x Non-white x Age | -0.007 | [-0.057,0.043] | 0.782 | -0.001 | [-0.015,0.012] | 0.850 |
| Lowest wealth x White x Age |  |  |  |  |  |  |
| Lowest wealth x Non-white x Age | 0.018 | [-0.031,0.068] | 0.466 | 0.003 | [-0.011,0.017] | 0.652 |
| Ref: Male |  |  |  |  |  |  |
| Female | 0.898 | [0.811,0.986] | <0.001 | 0.951 | [0.905,0.997] | <0.001 |
| Highest wealth x Male |  |  |  |  |  |  |
| Highest wealth x Female |  |  |  |  |  |  |
| Higher wealth x Male |  |  |  |  |  |  |
| Higher wealth x Female | -0.074 | [-0.175,0.027] | 0.149 | -0.048 | [-0.100,0.003] | 0.067 |
| Lower wealth x Male |  |  |  |  |  |  |
| Lower wealth x Female | -0.206 | [-0.339,-0.074] | 0.002 | -0.119 | [-0.184,-0.053] | <0.001 |
| Lowest wealth x Male |  |  |  |  |  |  |
| Lowest wealth x Female | -0.210 | [-0.334,-0.086] | 0.001 | -0.100 | [-0.163,-0.038] | 0.002 |
| Ref: Male x Age |  |  |  |  |  |  |
| Female x Age | -0.007 | [-0.018,0.003] | 0.173 | -0.010 | [-0.014,-0.006] | <0.001 |
| Highest wealth x Male x Age |  |  |  |  |  |  |
| Highest wealth x Female x Age |  |  |  |  |  |  |
| Higher wealth x Male x Age |  |  |  |  |  |  |
| Higher wealth x Female x Age | -0.001 | [-0.012,0.011] | 0.918 | 0.001 | [-0.003,0.006] | 0.625 |
| Lower wealth x Male x Age |  |  |  |  |  |  |
| Lower wealth x Female x Age | -0.001 | [-0.016,0.014] | 0.875 | 0.000 | [-0.005,0.006] | 0.991 |
| Lowest wealth x Male x Age |  |  |  |  |  |  |
| Lowest wealth x Female x Age | -0.000 | [-0.014,0.013] | 0.946 | 0.001 | [-0.005,0.006] | 0.739 |
| **Self-rated eyesight** |  |  |  |  |  |  |
| Age | 0.018 | [0.009,0.028] | <0.001 | 0.019 | [0.014,0.025] | <0.001 |
| Age^2^ | 0.001 | [0.001,0.001] | <0.001 | 0.000 | [0.000,0.000] | 0.047 |
| Ref: Highest wealth |  |  |  |  |  |  |
| Higher wealth | 0.105 | [0.012,0.198] | 0.027 | 0.062 | [0.017,0.107] | 0.007 |
| Lower wealth | 0.393 | [0.276,0.510] | <0.001 | 0.133 | [0.076,0.190] | <0.001 |
| Lowest wealth | 0.575 | [0.465,0.685] | <0.001 | 0.263 | [0.207,0.319] | <0.001 |
| Ref: White |  |  |  |  |  |  |
| Non-white | 0.182 | [-0.209,0.572] | 0.362 | 0.382 | [0.194,0.569] | <0.001 |
| Highest wealth x White |  |  |  |  |  |  |
| Highest wealth x Non-white |  |  |  |  |  |  |
| Higher wealth x White |  |  |  |  |  |  |
| Higher wealth x Non-white | 0.140 | [-0.276,0.555] | 0.510 | -0.157 | [-0.365,0.051] | 0.138 |
| Lower wealth x White |  |  |  |  |  |  |
| Lower wealth x Non-white | 0.103 | [-0.369,0.575] | 0.668 | -0.180 | [-0.424,0.065] | 0.149 |
| Lowest wealth x White |  |  |  |  |  |  |
| Lowest wealth x Non-white | 0.093 | [-0.344,0.529] | 0.677 | -0.069 | [-0.288,0.149] | 0.533 |
| Ref: Highest wealth x Age |  |  |  |  |  |  |
| Higher wealth x Age | -0.002 | [-0.012,0.008] | 0.681 | -0.002 | [-0.007,0.004] | 0.546 |
| Lower wealth x Age | -0.004 | [-0.017,0.008] | 0.499 | -0.006 | [-0.012,0.001] | 0.087 |
| Lowest wealth x Age | -0.004 | [-0.016,0.007] | 0.477 | -0.011 | [-0.018,-0.004] | 0.001 |
| Ref: White x Age |  |  |  |  |  |  |
| Non-white x Age | 0.005 | [-0.045,0.055] | 0.856 | -0.001 | [-0.024,0.022] | 0.931 |
| Highest wealth x White x Age |  |  |  |  |  |  |
| Highest wealth x Non-white x Age |  |  |  |  |  |  |
| Higher wealth x White x Age |  |  |  |  |  |  |
| Higher wealth x Non-white x Age | -0.008 | [-0.061,0.045] | 0.764 | -0.005 | [-0.031,0.020] | 0.676 |
| Lower wealth x White x Age |  |  |  |  |  |  |
| Lower wealth x Non-white x Age | -0.022 | [-0.078,0.035] | 0.452 | 0.003 | [-0.025,0.031] | 0.836 |
| Lowest wealth x White x Age |  |  |  |  |  |  |
| Lowest wealth x Non-white x Age | -0.006 | [-0.061,0.049] | 0.835 | 0.021 | [-0.006,0.049] | 0.133 |
| Ref: Male |  |  |  |  |  |  |
| Female | 0.113 | [0.001,0.224] | 0.047 | -0.031 | [-0.094,0.033] | 0.343 |
| Highest wealth x Male |  |  |  |  |  |  |
| Highest wealth x Female |  |  |  |  |  |  |
| Higher wealth x Male |  |  |  |  |  |  |
| Higher wealth x Female | -0.015 | [-0.140,0.110] | 0.812 | 0.047 | [-0.023,0.116] | 0.191 |
| Lower wealth x Male |  |  |  |  |  |  |
| Lower wealth x Female | -0.030 | [-0.184,0.125] | 0.705 | 0.046 | [-0.038,0.130] | 0.287 |
| Lowest wealth x Male |  |  |  |  |  |  |
| Lowest wealth x Female | -0.084 | [-0.229,0.060] | 0.253 | 0.086 | [0.006,0.167] | 0.036 |
| Ref: Male x Age |  |  |  |  |  |  |
| Female x Age | -0.005 | [-0.018,0.007] | 0.403 | -0.002 | [-0.010,0.006] | 0.678 |
| Highest wealth x Male x Age |  |  |  |  |  |  |
| Highest wealth x Female x Age |  |  |  |  |  |  |
| Higher wealth x Male x Age |  |  |  |  |  |  |
| Higher wealth x Female x Age | 0.007 | [-0.007,0.020] | 0.322 | 0.002 | [-0.007,0.011] | 0.636 |
| Lower wealth x Male x Age |  |  |  |  |  |  |
| Lower wealth x Female x Age | 0.010 | [-0.007,0.026] | 0.244 | 0.003 | [-0.007,0.014] | 0.520 |
| Lowest wealth x Male x Age |  |  |  |  |  |  |
| Lowest wealth x Female x Age | 0.007 | [-0.009,0.022] | 0.387 | 0.007 | [-0.004,0.017] | 0.205 |
| **Self-rated hearing** |  |  |  |  |  |  |
| Age | 0.024 | [0.014,0.033] | <0.001 | 0.038 | [0.032,0.043] | <0.001 |
| Age^2^ | 0.001 | [0.000,0.001] | <0.001 | -0.000 | [-0.000,0.000] | 0.947 |
| Ref: Highest wealth |  |  |  |  |  |  |
| Higher wealth | 0.016 | [-0.091,0.123] | 0.767 | 0.051 | [-0.003,0.104] | 0.065 |
| Lower wealth | 0.281 | [0.141,0.420] | <0.001 | 0.055 | [-0.014,0.125] | 0.118 |
| Lowest wealth | 0.051 | [-0.075,0.178] | 0.425 | 0.094 | [0.028,0.160] | 0.005 |
| Ref: White |  |  |  |  |  |  |
| Non-white | 0.059 | [-0.311,0.428] | 0.756 | 0.100 | [-0.108,0.309] | 0.346 |
| Highest wealth x White |  |  |  |  |  |  |
| Highest wealth x Non-white |  |  |  |  |  |  |
| Higher wealth x White |  |  |  |  |  |  |
| Higher wealth x Non-white | 0.012 | [-0.389,0.414] | 0.952 | -0.110 | [-0.350,0.130] | 0.368 |
| Lower wealth x White |  |  |  |  |  |  |
| Lower wealth x Non-white | -0.053 | [-0.481,0.375] | 0.808 | 0.012 | [-0.274,0.298] | 0.935 |
| Lowest wealth x White |  |  |  |  |  |  |
| Lowest wealth x Non-white | 0.028 | [-0.387,0.443] | 0.895 | -0.026 | [-0.285,0.233] | 0.844 |
| Ref: Highest wealth x Age |  |  |  |  |  |  |
| Higher wealth x Age | -0.004 | [-0.015,0.006] | 0.408 | -0.001 | [-0.007,0.005] | 0.837 |
| Lower wealth x Age | -0.012 | [-0.025,0.001] | 0.066 | -0.008 | [-0.015,-0.001] | 0.035 |
| Lowest wealth x Age | 0.007 | [-0.006,0.020] | 0.274 | -0.006 | [-0.013,0.002] | 0.165 |
| Ref: White x Age |  |  |  |  |  |  |
| Non-white x Age | -0.046 | [-0.091,-0.000] | 0.048 | 0.005 | [-0.018,0.027] | 0.695 |
| Highest wealth x White x Age |  |  |  |  |  |  |
| Highest wealth x Non-white x Age |  |  |  |  |  |  |
| Higher wealth x White x Age |  |  |  |  |  |  |
| Higher wealth x Non-white x Age | 0.060 | [0.010,0.109] | 0.018 | -0.009 | [-0.035,0.016] | 0.483 |
| Lower wealth x White x Age |  |  |  |  |  |  |
| Lower wealth x Non-white x Age | 0.054 | [-0.001,0.109] | 0.053 | 0.004 | [-0.025,0.032] | 0.809 |
| Lowest wealth x White x Age |  |  |  |  |  |  |
| Lowest wealth x Non-white x Age | 0.069 | [0.017,0.121] | 0.010 | 0.018 | [-0.010,0.045] | 0.209 |
| Ref: Male |  |  |  |  |  |  |
| Female | -0.524 | [-0.655,-0.394] | <0.001 | -0.303 | [-0.376,-0.230] | <0.001 |
| Highest wealth x Male |  |  |  |  |  |  |
| Highest wealth x Female |  |  |  |  |  |  |
| Higher wealth x Male |  |  |  |  |  |  |
| Higher wealth x Female | 0.107 | [-0.040,0.255] | 0.154 | 0.030 | [-0.050,0.111] | 0.461 |
| Lower wealth x Male |  |  |  |  |  |  |
| Lower wealth x Female | -0.016 | [-0.201,0.170] | 0.869 | 0.042 | [-0.056,0.140] | 0.403 |
| Lowest wealth x Male |  |  |  |  |  |  |
| Lowest wealth x Female | 0.342 | [0.173,0.512] | <0.001 | 0.086 | [-0.007,0.180] | 0.071 |
| Ref: Male x Age |  |  |  |  |  |  |
| Female x Age | 0.016 | [0.003,0.029] | 0.013 | 0.003 | [-0.006,0.011] | 0.500 |
| Highest wealth x Male x Age |  |  |  |  |  |  |
| Highest wealth x Female x Age |  |  |  |  |  |  |
| Higher wealth x Male x Age |  |  |  |  |  |  |
| Higher wealth x Female x Age | -0.011 | [-0.025,0.003] | 0.125 | 0.000 | [-0.009,0.010] | 0.922 |
| Lower wealth x Male x Age |  |  |  |  |  |  |
| Lower wealth x Female x Age | 0.001 | [-0.017,0.018] | 0.937 | 0.008 | [-0.003,0.018] | 0.170 |
| Lowest wealth x Male x Age |  |  |  |  |  |  |
| Lowest wealth x Female x Age | -0.025 | [-0.041,-0.008] | 0.004 | 0.002 | [-0.009,0.013] | 0.685 |
| **ADL difficulties, number square root** |  |  |  |  |  |  |
| Age | 0.007 | [0.002,0.011] | 0.003 | 0.011 | [0.009,0.013] | <0.001 |
| Age^2^ | 0.001 | [0.001,0.001] | <0.001 | 0.000 | [0.000,0.000] | <0.001 |
| Ref: Highest wealth |  |  |  |  |  |  |
| Higher wealth | 0.046 | [0.007,0.085] | 0.020 | 0.013 | [-0.000,0.026] | 0.054 |
| Lower wealth | 0.188 | [0.123,0.253] | <0.001 | 0.036 | [0.017,0.056] | <0.001 |
| Lowest wealth | 0.356 | [0.294,0.418] | <0.001 | 0.091 | [0.070,0.112] | <0.001 |
| Ref: White |  |  |  |  |  |  |
| Non-white | -0.065 | [-0.292,0.162] | 0.576 | -0.039 | [-0.092,0.014] | 0.152 |
| Highest wealth x White |  |  |  |  |  |  |
| Highest wealth x Non-white |  |  |  |  |  |  |
| Higher wealth x White |  |  |  |  |  |  |
| Higher wealth x Non-white | 0.212 | [-0.038,0.462] | 0.097 | -0.021 | [-0.082,0.041] | 0.512 |
| Lower wealth x White |  |  |  |  |  |  |
| Lower wealth x Non-white | 0.007 | [-0.289,0.302] | 0.965 | -0.060 | [-0.128,0.008] | 0.083 |
| Lowest wealth x White |  |  |  |  |  |  |
| Lowest wealth x Non-white | -0.057 | [-0.324,0.209] | 0.675 | -0.079 | [-0.146,-0.013] | 0.019 |
| Ref: Highest wealth x Age |  |  |  |  |  |  |
| Higher wealth x Age | 0.005 | [-0.000,0.010] | 0.070 | 0.000 | [-0.002,0.002] | 0.836 |
| Lower wealth x Age | 0.006 | [-0.001,0.013] | 0.099 | 0.001 | [-0.002,0.004] | 0.565 |
| Lowest wealth x Age | 0.011 | [0.004,0.019] | 0.001 | 0.002 | [-0.001,0.005] | 0.133 |
| Ref: White x Age |  |  |  |  |  |  |
| Non-white x Age | 0.008 | [-0.025,0.040] | 0.651 | -0.001 | [-0.007,0.005] | 0.659 |
| Highest wealth x White x Age |  |  |  |  |  |  |
| Highest wealth x Non-white x Age |  |  |  |  |  |  |
| Higher wealth x White x Age |  |  |  |  |  |  |
| Higher wealth x Non-white x Age | -0.007 | [-0.041,0.028] | 0.699 | -0.001 | [-0.007,0.006] | 0.885 |
| Lower wealth x White x Age |  |  |  |  |  |  |
| Lower wealth x Non-white x Age | -0.004 | [-0.042,0.034] | 0.836 | -0.000 | [-0.009,0.009] | 0.969 |
| Lowest wealth x White x Age |  |  |  |  |  |  |
| Lowest wealth x Non-white x Age | 0.000 | [-0.036,0.037] | 0.979 | 0.001 | [-0.007,0.009] | 0.773 |
| Ref: Male |  |  |  |  |  |  |
| Female | 0.015 | [-0.031,0.060] | 0.525 | 0.071 | [0.048,0.094] | <0.001 |
| Highest wealth x Male |  |  |  |  |  |  |
| Highest wealth x Female |  |  |  |  |  |  |
| Higher wealth x Male |  |  |  |  |  |  |
| Higher wealth x Female | 0.031 | [-0.024,0.086] | 0.274 | 0.039 | [0.013,0.065] | 0.004 |
| Lower wealth x Male |  |  |  |  |  |  |
| Lower wealth x Female | -0.003 | [-0.090,0.084] | 0.943 | 0.061 | [0.028,0.095] | <0.001 |
| Lowest wealth x Male |  |  |  |  |  |  |
| Lowest wealth x Female | -0.023 | [-0.106,0.060] | 0.582 | 0.089 | [0.055,0.124] | 0.000 |
| Ref: Male x Age |  |  |  |  |  |  |
| Female x Age | -0.001 | [-0.007,0.006] | 0.875 | 0.003 | [-0.000,0.006] | 0.052 |
| Highest wealth x Male x Age |  |  |  |  |  |  |
| Highest wealth x Female x Age |  |  |  |  |  |  |
| Higher wealth x Male x Age |  |  |  |  |  |  |
| Higher wealth x Female x Age | -0.004 | [-0.011,0.004] | 0.315 | <0.001 | [-0.003,0.004] | 0.815 |
| Lower wealth x Male x Age |  |  |  |  |  |  |
| Lower wealth x Female x Age | 0.004 | [-0.005,0.014] | 0.380 | 0.003 | [-0.001,0.008] | 0.129 |
| Lowest wealth x Male x Age |  |  |  |  |  |  |
| Lowest wealth x Female x Age | -0.007 | [-0.017,0.002] | 0.127 | 0.002 | [-0.002,0.006] | 0.342 |
| **IADL difficulties, number square root** |  |  |  |  |  |  |
| Age | 0.006 | [0.001,0.011] | 0.019 | 0.010 | [0.009,0.012] | <0.001 |
| Age^2^ | 0.002 | [0.001,0.002] | <0.001 | 0.001 | [0.001,0.001] | <0.001 |
| Ref: Highest wealth |  |  |  |  |  |  |
| Higher wealth | 0.035 | [-0.008,0.078] | 0.108 | -0.001 | [-0.010,0.008] | 0.830 |
| Lower wealth | 0.137 | [0.070,0.205] | <0.001 | 0.003 | [-0.010,0.016] | 0.652 |
| Lowest wealth | 0.368 | [0.301,0.435] | <0.001 | 0.047 | [0.031,0.064] | <0.001 |
| Ref: White |  |  |  |  |  |  |
| Non-white | -0.075 | [-0.357,0.207] | 0.601 | 0.011 | [-0.031,0.052] | 0.615 |
| Highest wealth x White |  |  |  |  |  |  |
| Highest wealth x Non-white |  |  |  |  |  |  |
| Higher wealth x White |  |  |  |  |  |  |
| Higher wealth x Non-white | 0.186 | [-0.116,0.488] | 0.227 | -0.009 | [-0.057,0.039] | 0.715 |
| Lower wealth x White |  |  |  |  |  |  |
| Lower wealth x Non-white | 0.172 | [-0.189,0.533] | 0.351 | -0.024 | [-0.086,0.037] | 0.439 |
| Lowest wealth x White |  |  |  |  |  |  |
| Lowest wealth x Non-white | 0.048 | [-0.283,0.379] | 0.776 | -0.012 | [-0.077,0.053] | 0.723 |
| Ref: Highest wealth x Age |  |  |  |  |  |  |
| Higher wealth x Age | 0.004 | [-0.001,0.010] | 0.129 | 0.002 | [0.000,0.003] | 0.029 |
| Lower wealth x Age | 0.011 | [0.004,0.019] | 0.004 | 0.003 | [0.001,0.006] | 0.001 |
| Lowest wealth x Age | 0.015 | [0.007,0.022] | <0.001 | 0.004 | [0.001,0.006] | 0.002 |
| Ref: White x Age |  |  |  |  |  |  |
| Non-white x Age | 0.025 | [-0.016,0.066] | 0.234 | 0.001 | [-0.005,0.006] | 0.864 |
| Highest wealth x White x Age |  |  |  |  |  |  |
| Highest wealth x Non-white x Age |  |  |  |  |  |  |
| Higher wealth x White x Age |  |  |  |  |  |  |
| Higher wealth x Non-white x Age | -0.016 | [-0.058,0.027] | 0.468 | 0.002 | [-0.005,0.009] | 0.572 |
| Lower wealth x White x Age |  |  |  |  |  |  |
| Lower wealth x Non-white x Age | -0.008 | [-0.057,0.041] | 0.744 | -0.002 | [-0.011,0.006] | 0.587 |
| Lowest wealth x White x Age |  |  |  |  |  |  |
| Lowest wealth x Non-white x Age | -0.008 | [-0.052,0.037] | 0.731 | 0.002 | [-0.006,0.010] | 0.635 |
| Ref: Male |  |  |  |  |  |  |
| Female | 0.062 | [0.009,0.115] | 0.021 | 0.019 | [0.003,0.034] | 0.018 |
| Highest wealth x Male |  |  |  |  |  |  |
| Highest wealth x Female |  |  |  |  |  |  |
| Higher wealth x Male |  |  |  |  |  |  |
| Higher wealth x Female | 0.018 | [-0.045,0.081] | 0.577 | 0.017 | [-0.001,0.034] | 0.066 |
| Lower wealth x Male |  |  |  |  |  |  |
| Lower wealth x Female | 0.023 | [-0.069,0.116] | 0.624 | 0.028 | [0.004,0.051] | 0.022 |
| Lowest wealth x Male |  |  |  |  |  |  |
| Lowest wealth x Female | -0.086 | [-0.177,0.005] | 0.065 | 0.050 | [0.024,0.076] | <0.001 |
| Ref: Male x Age |  |  |  |  |  |  |
| Female x Age | -0.002 | [-0.009,0.006] | 0.639 | 0.003 | [0.001,0.006] | 0.017 |
| Highest wealth x Male x Age |  |  |  |  |  |  |
| Highest wealth x Female x Age |  |  |  |  |  |  |
| Higher wealth x Male x Age |  |  |  |  |  |  |
| Higher wealth x Female x Age | 0.001 | [-0.008,0.009] | 0.900 | -0.002 | [-0.004,0.001] | 0.250 |
| Lower wealth x Male x Age |  |  |  |  |  |  |
| Lower wealth x Female x Age | 0.002 | [-0.009,0.012] | 0.779 | -0.000 | [-0.004,0.003] | 0.865 |
| Lowest wealth x Male x Age |  |  |  |  |  |  |
| Lowest wealth x Female x Age | -0.001 | [-0.011,0.009] | 0.871 | 0.001 | [-0.002,0.005] | 0.450 |
| Observations |  |  |  |  |  |  |
| **Mobility difficulties, number (ELSA) / Up and go time, s (CLSA)** |  |  |  |  |  |  |
| Age | 0.034 | [0.016,0.051] | <0.001 | 0.016 | [0.014,0.017] | <0.001 |
| Age^2^ | 0.004 | [0.003,0.004] | <0.001 | 0.000 | [0.000,0.000] | <0.001 |
| Ref: Highest wealth |  |  |  |  |  |  |
| Higher wealth | 0.212 | [0.034,0.390] | 0.020 | 0.007 | [-0.005,0.019] | 0.281 |
| Lower wealth | 1.003 | [0.697,1.309] | <0.001 | 0.024 | [0.006,0.041] | 0.007 |
| Lowest wealth | 2.194 | [1.904,2.484] | <0.001 | 0.061 | [0.045,0.076] | <0.001 |
| Ref: White |  |  |  |  |  |  |
| Non-white | 0.696 | [-0.426,1.819] | 0.224 | 0.054 | [0.013,0.095] | 0.009 |
| Highest wealth x White |  |  |  |  |  |  |
| Highest wealth x Non-white |  |  |  |  |  |  |
| Higher wealth x White |  |  |  |  |  |  |
| Higher wealth x Non-white | -0.188 | [-1.434,1.058] | 0.767 | -0.001 | [-0.056,0.053] | 0.965 |
| Lower wealth x White |  |  |  |  |  |  |
| Lower wealth x Non-white | -0.555 | [-2.000,0.890] | 0.452 | -0.022 | [-0.085,0.041] | 0.491 |
| Lowest wealth x White |  |  |  |  |  |  |
| Lowest wealth x Non-white | -0.885 | [-2.218,0.448] | 0.193 | 0.002 | [-0.051,0.056] | 0.929 |
| Ref: Highest wealth x Age |  |  |  |  |  |  |
| Higher wealth x Age | 0.017 | [-0.003,0.037] | 0.094 | 0.002 | [0.000,0.003] | 0.022 |
| Lower wealth x Age | 0.027 | [-0.001,0.055] | 0.062 | 0.002 | [-0.000,0.004] | 0.050 |
| Lowest wealth x Age | 0.021 | [-0.008,0.051] | 0.152 | 0.003 | [0.001,0.005] | 0.003 |
| Ref: White x Age |  |  |  |  |  |  |
| Non-white x Age | 0.001 | [-0.128,0.130] | 0.982 | -0.000 | [-0.006,0.005] | 0.879 |
| Highest wealth x White x Age |  |  |  |  |  |  |
| Highest wealth x Non-white x Age |  |  |  |  |  |  |
| Higher wealth x White x Age |  |  |  |  |  |  |
| Higher wealth x Non-white x Age | 0.031 | [-0.108,0.170] | 0.658 | 0.002 | [-0.005,0.009] | 0.534 |
| Lower wealth x White x Age |  |  |  |  |  |  |
| Lower wealth x Non-white x Age | -0.015 | [-0.180,0.150] | 0.863 | 0.003 | [-0.005,0.010] | 0.504 |
| Lowest wealth x White x Age |  |  |  |  |  |  |
| Lowest wealth x Non-white x Age | 0.054 | [-0.092,0.199] | 0.470 | -0.001 | [-0.008,0.006] | 0.828 |
| Ref: Male |  |  |  |  |  |  |
| Female | 0.467 | [0.228,0.706] | <0.001 | -0.030 | [-0.046,-0.014] | <0.001 |
| Highest wealth x Male |  |  |  |  |  |  |
| Highest wealth x Female |  |  |  |  |  |  |
| Higher wealth x Male |  |  |  |  |  |  |
| Higher wealth x Female | 0.337 | [0.051,0.623] | 0.021 | 0.018 | [-0.000,0.036] | 0.055 |
| Lower wealth x Male |  |  |  |  |  |  |
| Lower wealth x Female | 0.352 | [-0.083,0.788] | 0.113 | 0.035 | [0.011,0.059] | 0.004 |
| Lowest wealth x Male |  |  |  |  |  |  |
| Lowest wealth x Female | 0.018 | [-0.390,0.426] | 0.930 | 0.051 | [0.028,0.073] | 0.000 |
| Ref: Male x Age |  |  |  |  |  |  |
| Female x Age | 0.001 | [-0.025,0.027] | 0.930 | 0.002 | [-0.000,0.004] | 0.069 |
| Highest wealth x Male x Age |  |  |  |  |  |  |
| Highest wealth x Female x Age |  |  |  |  |  |  |
| Higher wealth x Male x Age |  |  |  |  |  |  |
| Higher wealth x Female x Age | -0.006 | [-0.035,0.024] | 0.714 | -0.001 | [-0.004,0.001] | 0.254 |
| Lower wealth x Male x Age |  |  |  |  |  |  |
| Lower wealth x Female x Age | -0.006 | [-0.046,0.033] | 0.763 | -0.003 | [-0.005,0.000] | 0.076 |
| Lowest wealth x Male x Age |  |  |  |  |  |  |
| Lowest wealth x Female x Age | 0.003 | [-0.036,0.042] | 0.876 | -0.001 | [-0.004,0.001] | 0.290 |

ELSA=English Longitudinal Study of Ageing; CLSA=Canadian Longitudinal Study on Aging; FEV=Forced Expiratory Volume; ADL=basic activities of daily living; IADL=instrumental activities of daily living. ^1^For measures where higher values originally indicated better function (gait speed, grip strength, and lung function), we reversed the direction by multiplying by −1. This transformation does not affect model fit or statistical inference but ensures consistent interpretation across outcomes. Models included sex, race (and their interaction with age), age^2^, birth year, and height (gait speed and grip strength analyses only) as covariates. For wealth associations, the highest category was the reference group.
